# Confounding Underlies the Apparent Month of Birth Effect in Multiple Sclerosis

**DOI:** 10.1002/ana.23925

**Published:** 2013-07-02

**Authors:** Barnaby Fiddes, James Wason, Anu Kemppinen, Maria Ban, Alastair Compston, Stephen Sawcer

**Affiliations:** 1 University of Cambridge, Department of Clinical Neurosciences, Addenbrooke's HospitalCambridge, United Kingdom; 2 Medical Research Council Biostatistics UnitCambridge, United Kingdom

## Abstract

**Objective:**

Several groups have reported apparent association between month of birth and multiple sclerosis. We sought to test the extent to which such studies might be confounded by extraneous variables such as year and place of birth.

**Methods:**

Using national birth statistics from 2 continents, we assessed the evidence for seasonal variations in birth rate and tested the extent to which these are subject to regional and temporal variation. We then established the age and regional origin distribution for a typical multiple sclerosis case collection and determined the false-positive rate expected when comparing such a collection with birth rates estimated by averaging population-specific national statistics.

**Results:**

We confirm that seasonality in birth rate is ubiquitous and subject to highly significant regional and temporal variations. In the context of this variation we show that birth rates observed in typical case collections are highly likely to deviate significantly from those obtained by the simple unweighted averaging of national statistics. The significant correlations between birth rates and both place (latitude) and time (year of birth) that characterize the general population indicate that the apparent seasonal patterns for month of birth suggested to be specific for multiple sclerosis (increased in the spring and reduced in the winter) are expected by chance alone.

**Interpretation:**

In the absence of adequate control for confounding factors, such as year and place of birth, our analyses indicate that the previous claims for association of multiple sclerosis with month of birth are probably false positives. ANN NEUROL 2013;73:714–720

Case–control studies are frequently employed as a means of testing for association between a disease of interest and candidate etiological risk factors. However, despite the apparent simplicity of this approach, spurious results are not uncommon^1^ and may easily arise if study design fails to avoid bias in the selection of study subjects.^2^ Because practical considerations mean that it is rarely possible to recruit cases and controls in an identical manner, it is usual to select study subjects from within a well-defined population, where exposure to the risk factor of interest is assumed to be distributed homogenously. However, bias may arise if the assumption of homogeneity is invalid, and subgroups that differ in the frequency of the candidate risk factor are concealed within the population from which cases and controls are drawn. In this situation, differences in ascertainment may result in biased representation of the relevant subgroups among the samples of cases and controls, thereby creating an apparent difference in exposure and a false-positive association. Furthermore, even if cases and controls are randomly selected from the whole population, false-positive associations may still occur if the frequency of the disease and of the candidate risk factor correlate across these subgroups (see the example from genetics below).^3^,^4^ Counterintuitively, such false-positive associations become increasingly more likely as sample size increases^5^; but do not occur (irrespective of sample size) if the study population is homogeneous with respect either to the frequency of the disease or to the candidate risk factor.^4^

In the analysis of month of birth, researchers have invariably assumed homogeneity within individual countries and have therefore felt justified in using averaged population statistics as controls. Here, we review the evidence that month of birth varies significantly with geographical location^6^–^8^ and over time^8^–^11^ in the normal population and consider the implication of this variation for case–control studies considering month of birth as a risk factor for the development of multiple sclerosis.

## Subjects and Methods

Using publically available national statistics, we established year-specific month of birth data sets (the number of births per month over a calendar year) for multiple years from 17 countries (Austria, Belgium, Canada, Denmark, Finland, France, Germany, Greece, Ireland, Italy, the Netherlands, Norway, Portugal, Spain, Sweden, Switzerland, and the United Kingdom), 51 North American regions (50 states and the District of Columbia), and 10 UK Government Office Regions (Supplementary Information). Data sets were tested for evidence of seasonality by comparison with the expected distribution of births in each month under the assumption of a uniform birth rate using a chi-square test (11 *df*). The chi-square test was also used to compare data sets within and between countries. In comparisons between consecutive years from the same country, the number of births in February was reduced by 1/29 for leap years, so that all such comparisons were based on data for 365 days. In these chi-square tests, we took *p* < 0.05 as evidence for a statistically significant difference, unless otherwise stated. To explore these complex data more formally, we used a multinomial logit model,^12^ which is a generalization of a logistic regression model that allows for >2 discrete outcomes (see Supplementary Information). We use this method to model how the probability of an individual being born in each month depends on different covariates, including latitude and the year of birth.

We generated a multiple sclerosis month of birth data set using records from the database we previously established through our UK nationwide effort to recruit patients for genetic studies. This database includes 15,765 affected index cases, of whom information on year and month of birth are available for 12,198 (see Supplementary Information).

To investigate the type I error rate of a typical month of birth analysis, we estimated the expected month of birth distribution in cases and controls using the UK Government Office Region data. For the cases, the distribution was weighted according to the regional prevalence of multiple sclerosis (see Structure and Risk Factors) and the observed year of birth, whereas the simple unweighted average was used for the controls. Because this analysis assumes homogeneity within each region, it is expected to underestimate the type I error rate. Two types of analyses were considered. First, we mirrored a discovery study, consisting of twelve 2 × 2 contingency tables, each comparing the number of case and control births in 1 month with the number in the other 11, and with significance declared if the Pearson chi-square test was significant after Bonferroni correction (*p* < 0.05/12 = 0.0042). Using the expected month of birth distribution in cases and controls, we determined the noncentrality parameter for each of these tests,^12^ and thereby calculated the probability of declaring evidence for a significant effect by chance alone—the type I error rate. The second analysis mirrored attempts to replicate the pattern previously suggested for multiple sclerosis,^13^–^16^ that is, finding a nominally significant (*p* < 0.05) excess of births in March, April, or May and/or a nominally significant (*p* < 0.05) deficit in November, December, or January. In all analyses, the numbers of cases and controls were equal.

## Results

**FIGURE 1:**
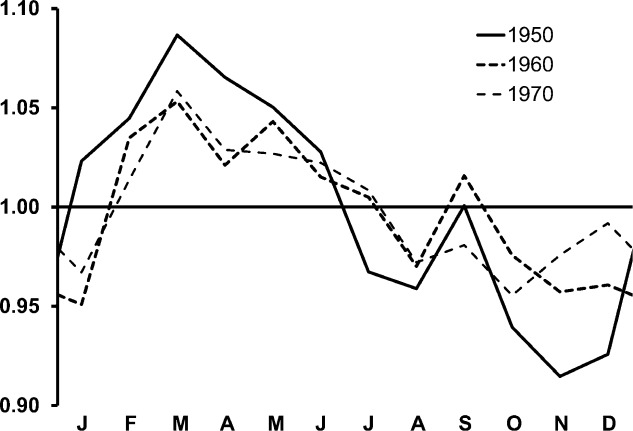
Seasonality seen in 3 typical month of birth data sets. The y-axis shows the average normalized daily birth rate, and the x-axis shows the months of the year (coded by their first letter). UK data for 3 years are shown; 1950 *(solid line)*, 1960 *(thick dashed line)*, and 1970 *(thin dashed line)*. The rates were calculated allowing for the length of each month and for leap years, but for simplicity are plotted assuming the length of each month is equal (1960 was a leap year, whereas 1950 and 1970 were not). Although our analysis does not depend upon identifying the systematic effects underlying the observed seasonality, one might speculate that, for example, the tendency for a higher than expected birth rate in September might be related in some way to the Christmas and New Year holiday season, that is, it might be primarily a cultural effect.

**FIGURE 2:**
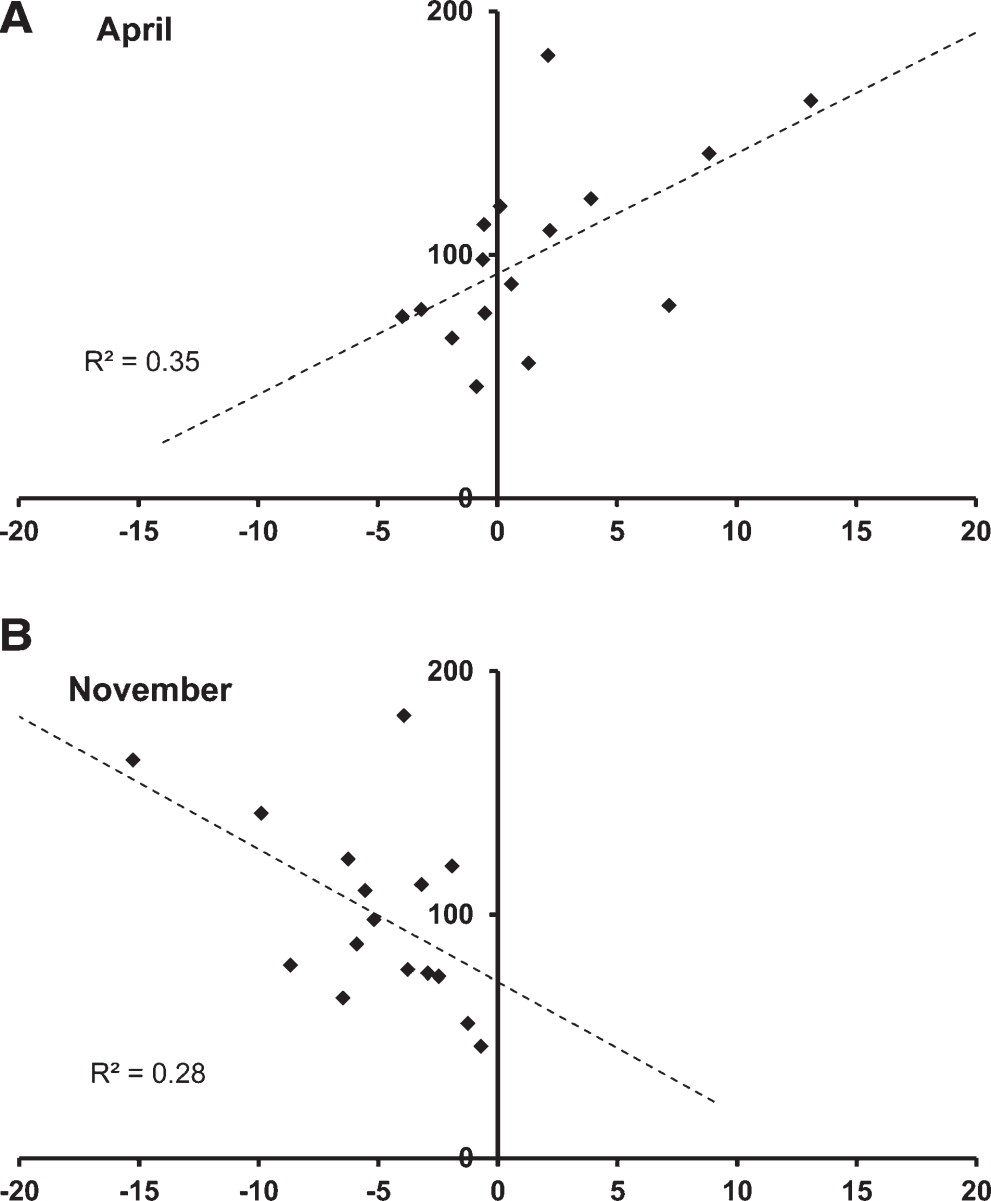
Correlation between the month-specific percentage excess in the average normalized daily birth rate (x-axis) and the prevalence of multiple sclerosis per 100,000 (y-axis) in 16 European countries (Austria, Belgium, Denmark, Finland, France, Germany, Greece, Ireland, Italy, the Netherlands, Norway, Portugal, Spain, Sweden, Switzerland, and the United Kingdom). (A) Data for April; (B) data for November. The birth rate estimates were calculated using the month of birth data sets for the decade 1991–2000. See the Supplementary Information for more details and for corresponding figures for other months.

Given the large number of cultural and biological factors that influence the timing of birth,^6^,^7^ it is perhaps not surprising that 98% of these 1,344 year- and population-specific month of birth data sets (824 from Europe and 520 from North America) show statistically significant evidence for seasonality (variation in birth rate through the year), the majority (>90%) at extreme levels of significance (*p* < 10^−6^). Considering the variation in birth rates seen for individual months and applying a Bonferroni correction (so that *p* < 0.0042 is considered significant), we found statistically significant evidence for seasonality, with excess births in either March, April, or May and/or reduced births in November, December, or January, mirroring the pattern described for multiple sclerosis,^13^–^15^ in 97% of European and 48% of North American data sets (see Supplementary Information). This apparent difference between continents primarily results from lower annual birth rates in many of the US states, which therefore lack the power to demonstrate observed differences as significant. In continent-specific pairwise comparisons, month of birth data sets showed significant differences both within and between populations (see Supplementary Information and Fig 1); again, the greatest differences were observed in the spring (March, April, and May) and winter (November, December, and January). As anticipated, our data confirm the expected correlations between seasonal birth rates and latitude^6^,^7^ and show that these correlations have diminished over time (see Supplementary Information).^8^–^11^ Given the known latitudinal gradient in the prevalence of multiple sclerosis, the positive correlation between prevalence and the probability of being born in the spring and the negative correlation with the probability of being born in winter are not unexpected (Fig 2).

To assess correlations within the month of birth data sets more rigorously, we used a multinomial logit model, including country as a factor, together with year of birth and whether that year was a leap year as covariates. Although this model explained only a small proportion of the variance in month of birth, the parameters representing the effect for each country were all highly significant, confirming that the distribution of month of birth differs significantly between countries. Fitting a second model that included latitude as an additional factor confirmed that the relationship between month of birth and latitude is statistically highly significant.

To test for the presence of regional heterogeneity in birth rates within individual populations, we considered 440 UK Government Office Region month of birth data sets (see Supplementary Information). More than 99% showed statistically significant evidence for seasonality, with 66% reproducing the pattern described for multiple sclerosis (statistically significant after Bonferroni correction); the most marked differences were seen in March and October. Comparing each Government Office Region month of birth data set with the set of UK controls employed in 1 influential study^13^ (n = 11,502) confirmed that 61% of these records include at least 1 month in which there is statistically significant evidence for a difference mimicking the pattern considered characteristic of multiple sclerosis. Even after scaling the Government Office Regions month of birth data sets to an equivalent size (n = 11,502), 33% continued to show at least 1 month with nominally significant evidence. Conversely, stratifying month of birth data sets into gender-specific components showed no difference in birth rates between males and females. This indicates that mismatching of gender between case collections and controls is unlikely to confound results, no matter how extreme the mismatch and irrespective of sample size.

**FIGURE 3:**
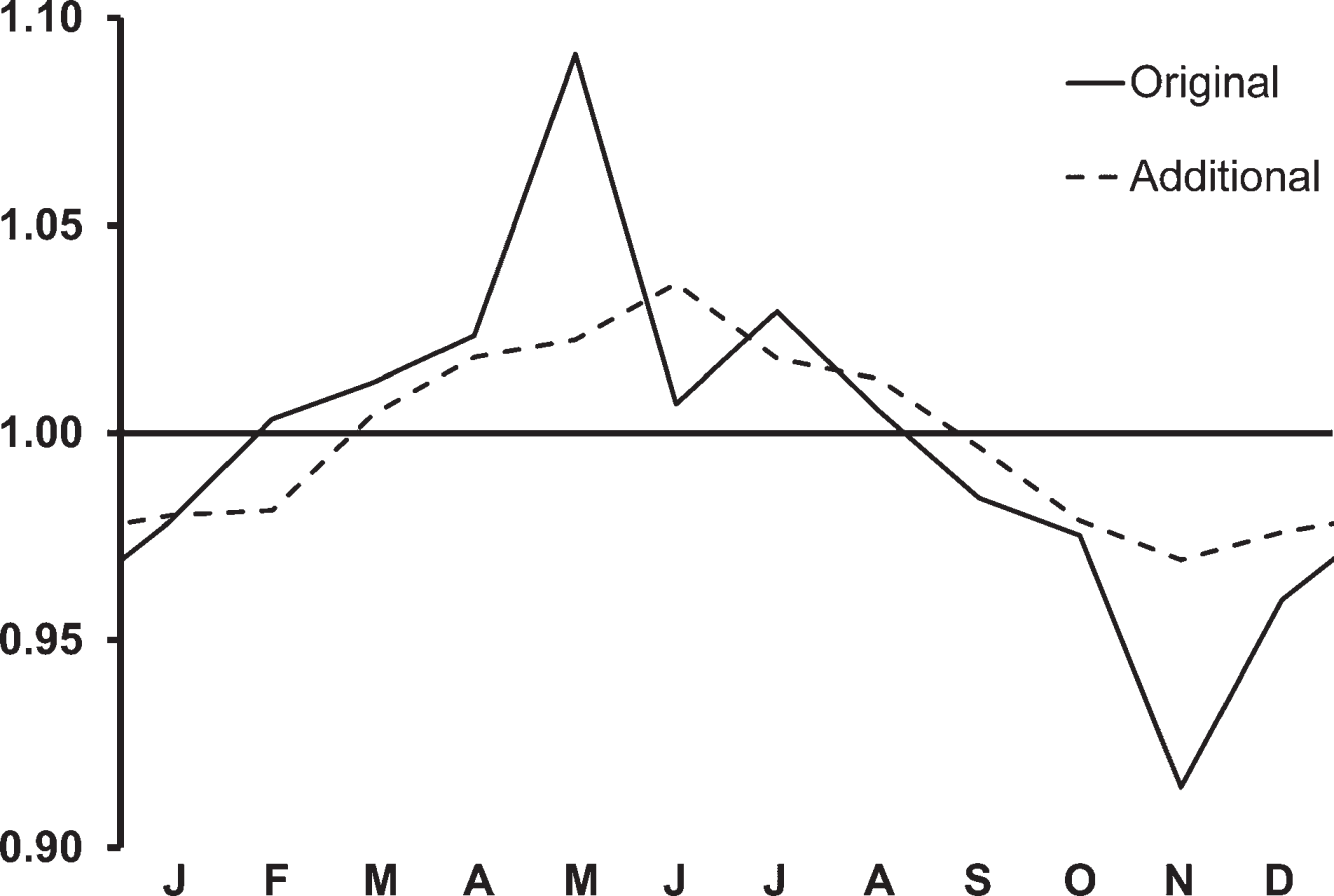
Odds ratio for each month as seen in the original report from Willer et al^13^ and in the combined analysis of published data,^13^–^15^,^17^ together with our own previously unpublished data (132,241 cases and controls). The expected (control) counts were as originally reported for the published data sets and unweighted (ie, crude) for the new UK data. These odds ratio estimates make no correction for the temporal and geographical structure in month of birth that is present in the general population. Months are coded by their first letters.

**FIGURE 4:**
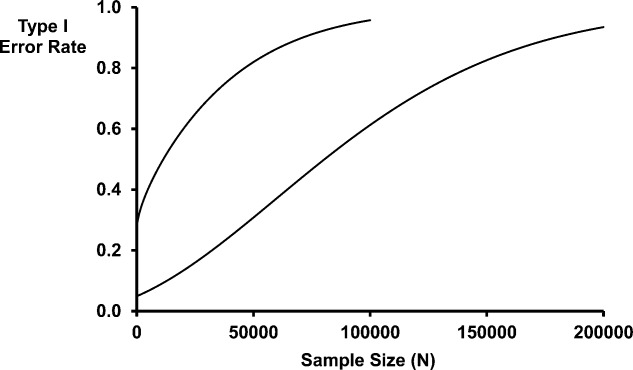
Lower limit of type I error rates (α) for typical population-specific multiple sclerosis month of birth studies of varying size (number of cases = number of controls = N). The lower curve indicates α for a discovery study, that is, the probability of seeing at least 1 month showing a significant association after Bonferroni correction (*p* < 0.05/12 = 0.0042), whereas the upper curve indicates α for a replication study, that is, the probability of at least 1 spring month showing a nominally significant (*p* < 0.05) excess in cases or at least 1 early winter month showing a nominally significant (*p* < 0.05) deficit in cases. These error rates are likely to be even greater in studies employing cases from >1 country, especially when these are high-risk (northern) countries such as Canada, Denmark, and Sweden, but would be reduced in countries where prevalence is more uniform. See the Supplementary Information for methodological details.

Comparing our sample of 12,198 UK multiple sclerosis cases with the expected numbers of births per month (calculated on the basis of a simple unweighted average across the 67 year-specific UK month of birth data sets obtained from the UK Office of National Statistics) showed that the greatest excess in births was seen in March (*p* = 0.06) and the greatest deficit in December (*p* = 0.03; see Supplementary Fig S13). Combining these data with those from northern hemisphere-based studies purporting to show a month of birth effect in multiple sclerosis^13^–^15^,^17^ generated a data set comprising 132,241 cases (after excluding duplicates). Comparing these cases with averaged population-specific month of birth data reproduces the putative multiple sclerosis pattern if an excess in June (*p* = 0.006) and a less significant deficit in November (*p* = 0.02: Fig 3) are taken as evidence for consistency. As would be anticipated, these results are similar to those reported in the recent meta-analysis of multiple sclerosis month of birth studies, which in total considered birth data from >150,000 cases.^16^ However, based on the type I error rates (Fig 4) we calculated using the UK Government Office Region data (see Subjects and Methods), there is a high probability that each of these modest residual signals of apparent association are false positive. Essentially, because all of the published studies employed simple unweighted averages of national statistics, none has adequately matched cases and controls for regional origin or year of birth, and thus all are likely to demonstrate an apparent association in keeping with the putative multiple sclerosis pattern by chance alone. The apparent latitudinal gradient in this effect noted in the meta-analysis^16^ is entirely expected as a reflection of the well-established, but under-recognized, gradient in seasonality of birth rates present in the general population.^6^–^8^

## Discussion

The analyses of genome-wide association studies (GWASs) have reminded researchers that seemingly homogeneous populations often turn out to be structured, and have also shown just how easily such effects are able to generate false-positive associations even in population-specific case–control studies (see the example from genetics below). Testing and making compensation for structure has become an indispensable part of complex genetics but seems to have been largely ignored in efforts to explore the possible role of environmental factors in complex traits. In our analysis of publicly available birth records, we confirm that birth rate is heterogeneous in the general population, where it is characterized by substantial and significant differences both between and within populations. It follows that birth rates calculated by the unweighted averaging of available population statistics are unlikely to provide appropriate controls for studies of specific diseases, where case collections are invariably heterogeneous with respect to year of birth and regional origin. Although it seems logical to average over many observations to generate more reliable control estimates, the mean birth rates obtained by such a process are a weighted average of the seasonality present in the population and not an estimate of some fundamental underlying rate. This weighted mean can only safely be compared with cases that have the same structure. Self-evidently, spurious differences will arise if birth rates are calculated for cases having a different distribution across subgroups that make up the normal population. Three groups^13^,^17^,^18^ have considered unaffected siblings as controls in an attempt to avoid such problems. However, although these related controls are inevitably much better matched for regional influences, they are necessarily unmatched for year of birth and are invariably limited in size and therefore underpowered (see Supplementary Information and Fig 4).

The confounding effects described here are a consequence of the substantial geographical and temporal variation in birth rate that is present in the general population; they are not specific to multiple sclerosis and have the potential to generate false-positive association with month of birth in any study where cases are inadequately matched, regardless of the phenotype. To date there are >500 reports relating month of birth to the etiology of a complex trait. The list includes various autoimmune diseases (celiac disease,^19^ diabetes,^20^ Graves disease,^21^ Hashimoto disease,^21^ inflammatory bowel disease,^22^ rheumatoid arthritis,^22^ and systemic lupus erythematous^22^), mental health disorders (schizophrenia^23^ and suicide^24^), and health-related traits (birth weight^25^). Because almost all of these studies are based on traits that are known to vary in frequency geographically and they have invariably used averaged national statistics as their source of controls, it is likely that many of these apparent associations resulted from confounding rather than any true biological effect.

The extent of variation in the seasonality of birth rates and correlation of this phenomenon with latitude and year of birth are surprising, underappreciated, and difficult to compensate for fully in the design and analysis of individual studies. Our observations serve as a reminder that risk factors that are easy to determine and seemingly homogenous, such as date of birth, may yet be heterogeneous within the general population and therefore generate false-positive signals if cases and controls are not adequately matched for the relevant extraneous variables. Efforts to identify and correct for confounding should be no less rigorous in the study of environmental risk factors than are now routine in the field of complex genetics.

### Key Points

National birth statistics show that within the general population: (1) birth rate is subject to highly significant variation with respect to place and time; and (2) the probability of being born in the spring (March, April, and May) is positively correlated with latitude, whereas the probability of being born in the winter (November, December, and January) is negatively correlated.

Because typical multiple sclerosis case collections are unlikely to be uniform with respect to region of origin or year of birth: (1) studies using national statistics as controls are predisposed to generate false-positive associations with month of birth; and (2) the associations generated by such studies are inherently biased in favor of showing a false-positive apparent excess of births in spring and/or reduced births in winter.

### The effects of structure: An Example from Genetics

Confounding due to structure is a well-recognized problem in complex genetics. Consider the data shown in the Table, which indicate the total population in each of the 11 Government Office Regions of the United Kingdom in mid-2010 (Office of national Statistics: http://www.ons.gov.uk), together with corresponding multiple sclerosis prevalence estimates and the approximate region-specific frequency of the C allele of the rs1042712 single nucleotide polymorphism (SNP) from the lactase gene (taken from Fig 7 of the supplementary file of the Wellcome Trust Case Control Consortium Genome Wide Association Study).^26^ Inspection of the Table shows that both the prevalence of multiple sclerosis and frequency of the C_rs1042712 allele vary geographically; prevalence correlates positively with latitude, whereas allele frequency is negatively correlated. Despite absolute differences in allele frequency only amounting to a few percentage points, the resulting structure is sufficient to confound association studies if ignored, and the UK population is wrongly assumed to be homogenous with respect to the frequency of this allele.

**TABLE:** Regional Variation in the Prevalence of Multiple Sclerosis (per 100,000) and the Frequency (%) of the Lactase Single Nucleotide Polymorphism Allele (C_rs1042712) in the United Kingdom

GOR	Population	Prevalence	C_rs1042712, %
Scotland	5,222,100	229	9.5

Northeast	2,606,625	*193*^a^	9.8

Northwest	6,935,736	*191*^a^	9.4

Yorkshire and Humber	5,301,252	109	10.9

Wales	3,006,430	146	9.5

East Midlands	4,481,431	*160*^a^	12.1

West Midlands	5,455,179	*152*^a^	11.1

East	5,831,845	152	13.3

London	7,825,177	115	14.5

Southeast	8,523,074	111	12.5

Southwest	5,273,726	118	11.6

Overall	60,462,575	147.88	11.58

^a^These prevalence figures were estimated assuming a linear relationship between latitude and prevalence and using the estimates from Aberdeen and Southwest England as reference values; otherwise figures are based on published studies representative of the region (see the Supplementary Information for details of prevalence studies used).

GOR = Government Office Region.

If controls are randomly selected from across the United Kingdom (as, for example, they would tend to be in a birth cohort), the expected allele frequency is 11.6%, less than is seen in London (14.5%) and more than in Wales (9.5%). If an association study were performed in London using 10,000 such controls and 2,000 local patients, it would find nominally significant evidence (*p* < 0.05) that C_rs1042712 is a risk factor for multiple sclerosis, with >99.9% certainty, and would generate a genome-wide significant result (*p* < 5 × 10^−8^) >40% of the time. If the study were then repeated using a similar design but with 2,000 patients from the East of England Government Office Region, the apparent association would be replicated with 1-tailed nominal significance on the majority of occasions (>90%). Conversely, if replication were attempted in Wales, researchers would most likely (>95%) find nominally significant evidence that C_rs1042712 appears protective. The ease with which apparently significant results can be generated despite the relatively modest absolute difference in allele frequency illustrates how sensitive case–control analysis is to unrecognized, and therefore uncompensated, population structure.

Even if everyone in the UK population were genotyped for the rs1042712 SNP, enabling all cases to be compared with all controls, nominally significant evidence that C_rs1042712 is associated (protective) would be observed on >95% of occasions. This bias arises because a disproportionate number of the cases will come from northern (high prevalence) parts of the country, which generally have a lower than average allele frequency for this particular SNP; as a result the estimated allele frequency in cases will tend to be lower (11.3%) than in the general population (11.6%). This illustrates why, even if all cases and all controls from a country are included in an analysis, structure can still generate false-positive associations unless there is compensation for the confounding effects of their unequal distribution. Whether a study that only employs cases and controls from a single Government Office Region would be free from bias cannot be judged from the figures provided in the Table. Given the highly significant difference in both disease and allele frequency between regions, it seems probable that structure will also occur within, although to a lesser extent than between, regions.

Genetic analyses are uniquely well placed with respect to the assessment and compensation of structure. First, although the above analysis has considered matters from a geographical perspective, the primary confounding variable in genetic studies is ancestry, not geography. The probability that individuals carry a particular allele of interest primarily depends upon who their ancestors were and not where they were born or domiciled when recruited to a study. Because ancestry does not change over time, and can be accurately inferred using genetic markers, in principle little if any geographical precision is necessary to assess and correct for structure in allele frequency distribution within a population. Conversely, for social and mobility reasons, there is an inevitable correlation between geography and ancestry,^27^ and this relationship means that geography often provides a reasonable, and useful, surrogate for ancestry as illustrated above. Second, because GWASs typically include many thousands of markers that are not associated with the particular disease of interest, these additional neutral data allow researchers both to measure^28^ and to compensate for differences in the ancestry of cases and controls.^29^ By comparing allele frequency distributions across regions of the United Kingdom, the Wellcome Trust Case Control Consortium established that most common variants show little variation in allele frequency within populations, the rs1042712 SNP from the lactase gene being 1 of only a limited number of exceptions to this rule.^26^ Available evidence suggests that this SNP is part of a haplotype that has been subject to considerable selection in the recent past and hence is now highly structured in the population.^30^ However, for the vast majority of common variants studied in GWASs, there is only limited structure within and between populations, and this can usually be compensated for using ancestral information from the thousands of unassociated markers that are inevitably typed as part of a GWAS.
