# Supplementary material for: Confounding Underlies the Apparent Month of Birth Effect in Multiple Sclerosis
Source: Ann Neurol. 2013 Jul 2;73(6):714–20. doi: 10.1002/ana.23925 (PMC3748787; doi:10.1002/ana.23925)
Supplement: Supplementary file 1 [file ana0073-0714-SD1.docx]

**Confounding underlies the apparent ‘month of birth’ effect in multiple sclerosis**

**Authors:** Barnaby Fiddes, MRCP,^1^ James Wason, PhD^2^, Anu Kemppinen, PhD,^1^ Maria Ban PhD^1^, Alastair Compston, PhD^1^ and Stephen Sawcer, PhD^1^

**Affiliations:**

^1^University of Cambridge, Department of Clinical Neuroscience, Addenbrooke’s, Hospital, Hills Road, Cambridge, CB2 2QQ, UK

^2^Medical Research Council Biostatistics Unit, Cambridge CB2 0SR, UK

Table of Contents

Month of birth (MOB) data sets from national statistics 3

Average normalised daily birth rate per month 4

Variation in and between population level MOB data sets 4

Multiple sclerosis 21

Multinomial modelling 31

UK Government Office Region level MOB data sets 32

MOB data from our own multiple sclerosis database 33

Testing for the effect of MOB on Mortality 36

Using unaffected siblings as controls 36

Heat map (choropleth) representations of seasonal excess birth rate data 38

References 40

# Month of birth (MOB) data sets from national statistics

Using the Eurostat portal (http://epp.eurostat.ec.europa.eu/) supplemented by population specific resources we established a total of 824 year specific MOB data sets (numbers of births per month) from across 16 European countries, see table S1. As anticipated MOB data sets were more readily available for recent years and scarce for those from earlier in the century; and are thus unlikely to fully overlap with the year of birth (YOB) spectrum expected in a typical set of prevalent cases of an adult onset disease such as multiple sclerosis (see below).

**Table S1** Years for which we were able to obtain the number of births per month in each of 16 European countries.

| **Country** | **Years with available data (inclusive)** | **Additional source** |
| --- | --- | --- |
| Austria | 1971-1980,1991-2000 |  |
| Belgium | 1900, 1901, 1905, 1910, 1913, 1920, 1925, 1930, 1935, 1940, 1945, 1950-2007 | FPS Economie Belgium http://economie.fgov.be/ |
| Denmark | 1950-2007 |  |
| Finland | 1900-2008 | Statistics Finland (STAT) http://www.stat.fi/ |
| France | 1946-2011 | Institut national de la statistique et des études économiques (INSEE) www.insee.fr/ |
| Germany | 1946-1989, 1991-2000 | Statistisches Bundesamt Deutchland (DESTATIS) www.destatis.de/ |
| Greece | 1971-1980, 1991-2000 |  |
| Ireland | 1955-1985, 1991-2000 | Northern Ireland Statistics and Research Agency (NISRA) www.nisra.gov.uk/ |
| Italy | 1940, 1950-1954, 1956, 1957, 1960, 1965, 1969, 1971-2007 | Istituto Nazionale di Statistica (ISTAT) www.istat.it/ |
| Netherlands | 1971-1980, 1991-2000 |  |
| Norway | 1921-2007 | Statistics Norway (SSB) www.ssb.no/ |
| Portugal | 1971-1980, 1991-2000 |  |
| Spain | 1941-2007 | Instituto Nacional de Estadística www.ine.es/ |
| Sweden | 1950-2007 |  |
| Switzerland | 1971-1980, 1991-2000 |  |
| UK (England and Wales) | 1938-2004 | UK National Statistics Office www.statistics.gov.uk |

We also established the number of births per month for 51 US regions (50 states and the district of Columbia) and in Canada for the years 1991-2000 (inclusive) using data from the US National Center for Health Statistics (http://www.cdc.gov/nchs/) and Statistics Canada (http://www.statcan.gc.ca/); 520 data sets in total.

In our analysis of finer level geographical variation in birth rates we used Government Office Region (GOR) level data from the years 1965-2008, as obtained from the UK Office of national statistics (www.statistics.gov.uk, for England and Wales) and the General Registry Office for Scotland (http://www.gro-scotland.gov.uk/). N.B. in Scotland only month of birth registration rather than actual month of birth was available for the years we considered. It should be noted that the vast majority of births are registered within a few weeks of the actual birth so this is unlikely to have any substantial effect. For each country, American state and UK GOR we determined the latitude and longitude of the central position of the main land mass using an internet based geocoding tool (http://global.mapit.mysociety.org/).

# Average normalised daily birth rate per month

For each MOB data set we calculated the average normalised daily birth rate in each month ($r_{j}$) as

$$r_{j}=\frac{\left( {n_{j}}/{d_{j}} \right)}{\left( N/D \right)}$$

where

$n_{j}$ = the number of births in the month j (1=January to 12=December)

$d_{j}$ = the number of days in the month j (28, 29, 30 or 31 accordingly)

$N$ = the number of births in the year $=\sum_{j=1}^{12} n_{j}$

$D$ = the number of days in the year $=\sum_{j=1}^{12} d_{j}$ = 365 for ordinary years and 366 for leap years.

The % excess in the average normalised daily birth rate in month j is then given by

$$100*\left( r_{j}-1 \right)$$

# Variation in and between population level MOB data sets

The proportion of MOB data sets showing an excess or a deficit in the average normalised daily birth rate in each month is shown in figures S1 (Europe) and S2 (North America). The proportion of data sets showing a statistically significant excess/deficit is smaller in North America because of the large number of states with modest annual birth rates.

Within the 16 European countries 88% of year specific data sets were statistically significantly different from the MOB data seen in the same country in the preceding year. In North America (51 states and Canada) 49% of such comparisons were statistically significantly different; on both continents most (approximately two thirds) of the MOB data sets not showing statistically significant differences were from countries/states with an annual birth rate of <60000. We next made all possible pairwise comparisons between the MOB data sets from a single year (1991) across the 16 European countries and found only one (Netherlands cf. Austria) that failed to show a statistically significant difference. We repeated this in North America for the 51 regions and Canada and found a statistically significant difference in 65% of pairs; again most (>90%) of those pairwise comparisons failing to reach significance involved a state with an annual birth rate of <60000. The mean and standard deviation for the average normalised daily birth rate in each month in each country/state is shown in tables S2 and S3.


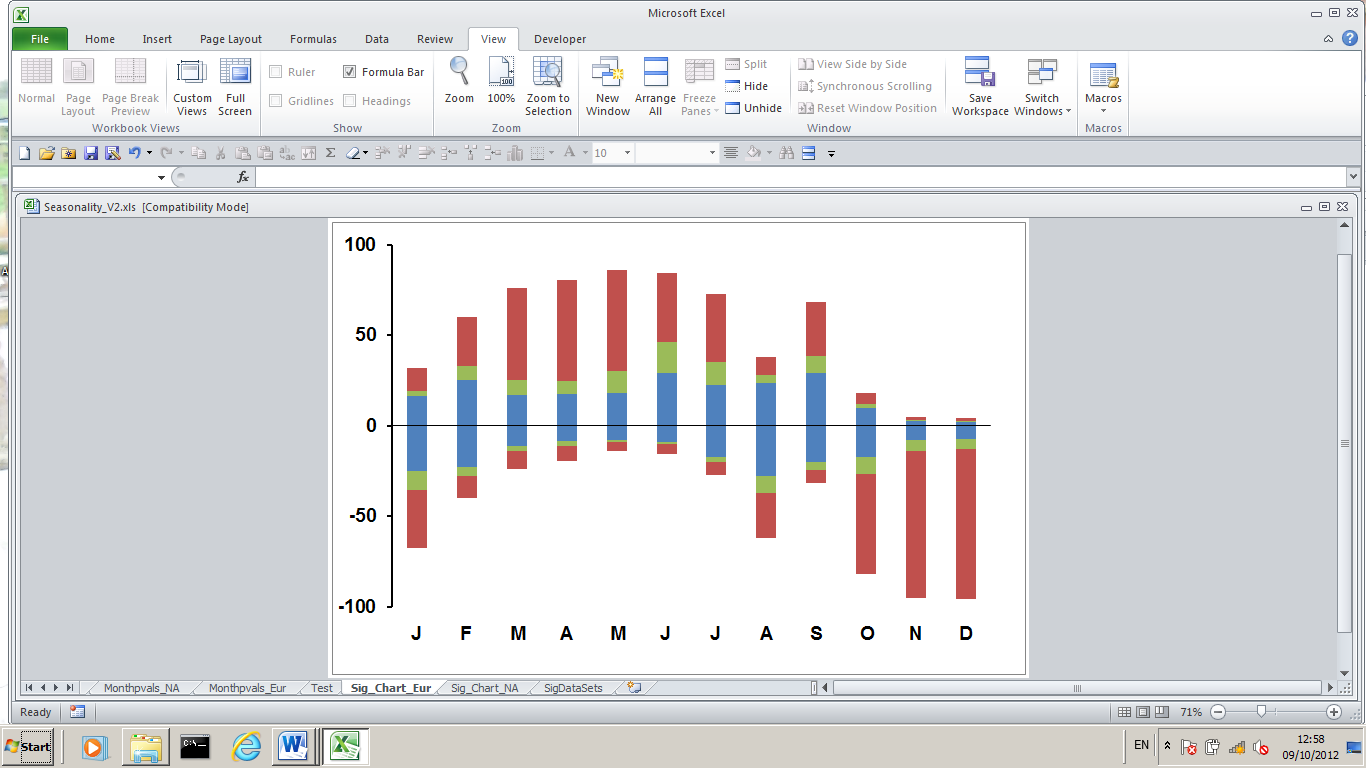


**Figure S1.** The proportion (%) of European MOB data sets (n=824) showing excess (above) or deficit (below) in the average normalised daily birth rate in each month. The proportion (%) of data sets showing significant difference with p<0.0045 (=0.05/11) is shown in red while the proportion with p<0.05 is shown in green, the proportion that is not statistically significantly different is shown in blue.


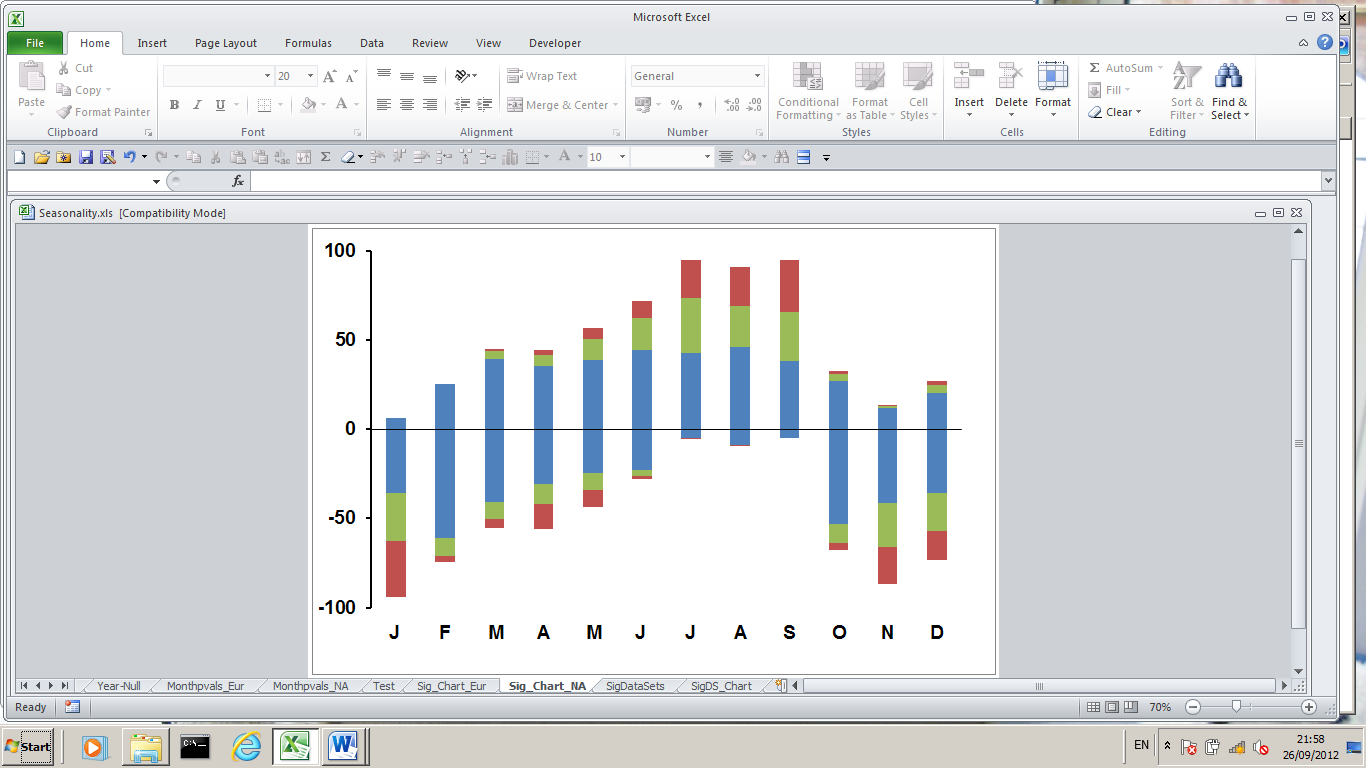


**Figure S2.** The proportion (%) of North American MOB data sets (n=824) showing excess (above) or deficit (below) in the average normalised daily birth rate in each month.

**Table S2.**The mean and standard deviation of the average normalised daily birth rate in 17 countries

| **Country** | **Y** | **N** | **January** | **February** | **March** | **April** | **May** | **June** |
| --- | --- | --- | --- | --- | --- | --- | --- | --- |
| Austria | 20 | 91 | 1.014 (0.029) | 1.037 (0.027) | 1.024 (0.035) | 1.010 (0.027) | 1.004 (0.023) | 1.006 (0.018) |
| Belgium | 69 | 136 | 0.991 (0.032) | 1.024 (0.041) | 1.029 (0.034) | 1.033 (0.033) | 1.030 (0.031) | 1.025 (0.038) |
| Canada | 10 | 368 | 0.945 (0.015) | 0.995 (0.009) | 1.031 (0.012) | 1.057 (0.019) | 1.064 (0.009) | 1.051 (0.012) |
| Denmark | 58 | 69 | 0.949 (0.032) | 1.009 (0.034) | 1.055 (0.045) | 1.074 (0.043) | 1.041 (0.033) | 1.033 (0.028) |
| Finland | 109 | 75 | 1.001 (0.066) | 1.010 (0.052) | 1.054 (0.057) | 1.049 (0.078) | 1.035 (0.068) | 1.047 (0.053) |
| France | 66 | 797 | 0.980 (0.029) | 0.999 (0.029) | 1.007 (0.035) | 1.028 (0.038) | 1.047 (0.038) | 1.028 (0.024) |
| Germany | 54 | 988 | 0.997 (0.058) | 1.035 (0.048) | 1.039 (0.042) | 1.021 (0.034) | 1.021 (0.029) | 1.014 (0.022) |
| Greece | 20 | 123 | 1.014 (0.045) | 0.996 (0.042) | 0.989 (0.049) | 0.976 (0.034) | 1.017 (0.039) | 1.043 (0.028) |
| Ireland | 41 | 45 | 1.008 (0.047) | 0.978 (0.029) | 1.012 (0.043) | 1.039 (0.042) | 1.048 (0.042) | 1.043 (0.041) |
| Italy | 48 | 688 | 1.013 (0.080) | 0.988 (0.061) | 0.991 (0.042) | 0.977 (0.039) | 1.019 (0.042) | 1.020 (0.050) |
| Netherlands | 20 | 192 | 0.975 (0.025) | 1.005 (0.028) | 1.020 (0.044) | 1.034 (0.048) | 1.030 (0.043) | 1.030 (0.031) |
| Norway | 87 | 57 | 0.968 (0.040) | 1.024 (0.034) | 1.076 (0.033) | 1.121 (0.040) | 1.069 (0.031) | 1.030 (0.020) |
| Portugal | 20 | 143 | 0.997 (0.028) | 0.976 (0.026) | 0.992 (0.021) | 1.003 (0.018) | 1.041 (0.028) | 1.009 (0.027) |
| Spain | 67 | 547 | 0.997 (0.046) | 1.013 (0.048) | 1.020 (0.041) | 1.023 (0.028) | 1.022 (0.029) | 0.995 (0.028) |
| Sweden | 58 | 106 | 0.972 (0.027) | 1.044 (0.029) | 1.110 (0.035) | 1.143 (0.037) | 1.080 (0.020) | 1.033 (0.023) |
| Switzerland | 20 | 81 | 0.994 (0.020) | 1.034 (0.024) | 1.047 (0.043) | 1.054 (0.039) | 1.041 (0.039) | 1.037 (0.024) |
| UK | 67 | 719 | 0.983 (0.035) | 1.011 (0.033) | 1.038 (0.042) | 1.024 (0.032) | 1.035 (0.025) | 1.024 (0.019) |
|  |  |  |  |  |  |  |  |  |
| **Country** | **Y** | **N** | **July** | **August** | **September** | **October** | **November** | **December** |
| Austria | 20 | 91 | 1.026 (0.023) | 1.000 (0.022) | 1.025 (0.034) | 0.960 (0.032) | 0.947 (0.027) | 0.950 (0.026) |
| Belgium | 69 | 136 | 1.020 (0.032) | 0.993 (0.029) | 1.006 (0.031) | 0.970 (0.031) | 0.936 (0.023) | 0.946 (0.032) |
| Canada | 10 | 368 | 1.039 (0.009) | 0.998 (0.013) | 1.034 (0.016) | 0.952 (0.010) | 0.922 (0.011) | 0.914 (0.020) |
| Denmark | 58 | 69 | 1.023 (0.040) | 1.005 (0.044) | 1.021 (0.032) | 0.956 (0.031) | 0.929 (0.032) | 0.906 (0.035) |
| Finland | 109 | 75 | 1.039 (0.048) | 0.994 (0.056) | 1.008 (0.075) | 0.917 (0.065) | 0.915 (0.055) | 0.931 (0.058) |
| France | 66 | 797 | 1.035 (0.022) | 0.990 (0.020) | 1.000 (0.026) | 0.971 (0.036) | 0.953 (0.034) | 0.963 (0.027) |
| Germany | 54 | 988 | 1.019 (0.035) | 0.994 (0.037) | 1.033 (0.038) | 0.952 (0.035) | 0.938 (0.027) | 0.941 (0.032) |
| Greece | 20 | 123 | 1.071 (0.041) | 1.023 (0.027) | 1.031 (0.040) | 1.002 (0.031) | 0.957 (0.027) | 0.880 (0.045) |
| Ireland | 41 | 45 | 1.029 (0.028) | 0.982 (0.039) | 1.020 (0.039) | 0.990 (0.031) | 0.953 (0.048) | 0.897 (0.066) |
| Italy | 48 | 688 | 1.050 (0.055) | 1.012 (0.033) | 1.051 (0.037) | 1.005 (0.038) | 0.953 (0.033) | 0.920 (0.043) |
| Netherlands | 20 | 192 | 1.028 (0.018) | 1.012 (0.027) | 1.025 (0.045) | 0.969 (0.041) | 0.947 (0.035) | 0.928 (0.024) |
| Norway | 87 | 57 | 1.003 (0.030) | 0.967 (0.031) | 1.021 (0.028) | 0.931 (0.032) | 0.890 (0.034) | 0.904 (0.035) |
| Portugal | 20 | 143 | 1.012 (0.031) | 0.987 (0.023) | 1.046 (0.034) | 1.002 (0.030) | 0.978 (0.033) | 0.956 (0.027) |
| Spain | 67 | 547 | 1.001 (0.034) | 0.978 (0.027) | 1.026 (0.031) | 0.995 (0.031) | 0.965 (0.038) | 0.966 (0.039) |
| Sweden | 58 | 106 | 1.003 (0.035) | 0.969 (0.038) | 0.991 (0.023) | 0.923 (0.027) | 0.875 (0.029) | 0.862 (0.036) |
| Switzerland | 20 | 81 | 1.028 (0.022) | 0.977 (0.030) | 1.010 (0.042) | 0.939 (0.032) | 0.920 (0.030) | 0.923 (0.030) |
| UK | 67 | 719 | 1.015 (0.021) | 0.988 (0.024) | 1.018 (0.030) | 0.970 (0.032) | 0.946 (0.036) | 0.949 (0.035) |

Y indicates the number of years of data considered in that country

N indicates the mean number of births per year in thousands

For each month the mean and (standard deviation) are shown

**Table S3.**The mean and standard deviation of the average normalised daily birth rate in 50 states and the District of Columbia (1991-2000).

| State | N | January | February | March | April | May | June |
| --- | --- | --- | --- | --- | --- | --- | --- |
| Alabama | 62 | 0.985 (0.015) | 0.991 (0.021) | 0.977 (0.022) | 0.945 (0.018) | 0.960 (0.017) | 0.987 (0.021) |
| Alaska | 11 | 0.944 (0.032) | 0.985 (0.036) | 1.023 (0.022) | 1.030 (0.040) | 1.019 (0.028) | 1.016 (0.045) |
| Arizona | 74 | 0.965 (0.015) | 0.978 (0.014) | 0.975 (0.019) | 0.952 (0.025) | 0.945 (0.019) | 0.971 (0.020) |
| Arkansas | 36 | 0.976 (0.016) | 0.989 (0.027) | 0.960 (0.022) | 0.947 (0.021) | 0.953 (0.019) | 0.990 (0.027) |
| California | 555 | 0.965 (0.010) | 0.980 (0.012) | 0.991 (0.013) | 0.976 (0.017) | 0.981 (0.015) | 1.000 (0.011) |
| Colorado | 57 | 0.922 (0.014) | 0.977 (0.012) | 1.002 (0.018) | 1.022 (0.029) | 1.032 (0.013) | 1.035 (0.014) |
| Connecticut | 45 | 0.942 (0.021) | 0.993 (0.013) | 1.002 (0.023) | 1.019 (0.024) | 1.034 (0.011) | 1.043 (0.017) |
| Delaware | 11 | 0.953 (0.029) | 0.995 (0.031) | 1.011 (0.046) | 1.003 (0.049) | 1.014 (0.029) | 1.014 (0.036) |
| District of Columbia | 9 | 1.034 (0.027) | 1.020 (0.032) | 0.997 (0.026) | 0.945 (0.032) | 0.981 (0.031) | 1.001 (0.042) |
| Florida | 194 | 0.980 (0.019) | 0.987 (0.014) | 0.969 (0.017) | 0.950 (0.011) | 0.955 (0.015) | 0.970 (0.019) |
| Georgia | 117 | 0.971 (0.011) | 0.988 (0.015) | 0.980 (0.020) | 0.954 (0.018) | 0.962 (0.013) | 0.983 (0.018) |
| Hawaii | 19 | 0.993 (0.039) | 0.997 (0.043) | 0.987 (0.030) | 0.995 (0.024) | 0.987 (0.022) | 0.976 (0.020) |
| Idaho | 18 | 0.907 (0.018) | 0.965 (0.031) | 0.997 (0.025) | 1.027 (0.019) | 1.050 (0.041) | 1.060 (0.030) |
| Illinois | 187 | 0.949 (0.013) | 0.986 (0.015) | 0.996 (0.018) | 0.979 (0.024) | 1.004 (0.018) | 1.026 (0.018) |
| Indiana | 85 | 0.949 (0.015) | 0.987 (0.015) | 0.994 (0.017) | 0.981 (0.021) | 1.000 (0.020) | 1.022 (0.022) |
| Iowa | 38 | 0.930 (0.025) | 0.987 (0.020) | 1.001 (0.024) | 1.009 (0.028) | 1.013 (0.031) | 1.017 (0.028) |
| Kansas | 38 | 0.947 (0.017) | 1.000 (0.017) | 0.991 (0.019) | 0.989 (0.031) | 0.995 (0.027) | 1.015 (0.022) |
| Kentucky | 54 | 0.957 (0.018) | 0.990 (0.011) | 0.983 (0.012) | 0.961 (0.027) | 0.986 (0.024) | 1.007 (0.028) |
| Louisiana | 68 | 0.987 (0.015) | 0.980 (0.027) | 0.942 (0.016) | 0.920 (0.016) | 0.929 (0.015) | 0.975 (0.026) |
| Maine | 14 | 0.936 (0.042) | 0.991 (0.019) | 1.013 (0.027) | 1.044 (0.031) | 1.049 (0.018) | 1.063 (0.038) |
| Maryland | 74 | 0.957 (0.018) | 0.991 (0.014) | 1.002 (0.019) | 0.994 (0.018) | 0.999 (0.022) | 1.021 (0.018) |
| Massachusetts | 83 | 0.938 (0.010) | 0.974 (0.012) | 1.002 (0.025) | 1.016 (0.017) | 1.046 (0.008) | 1.053 (0.021) |
| Michigan | 138 | 0.943 (0.010) | 1.000 (0.010) | 1.017 (0.016) | 1.016 (0.018) | 1.035 (0.018) | 1.041 (0.018) |
| Minnesota | 65 | 0.912 (0.019) | 0.971 (0.011) | 1.017 (0.018) | 1.029 (0.019) | 1.049 (0.024) | 1.046 (0.024) |
| Mississippi | 42 | 0.983 (0.023) | 0.980 (0.032) | 0.956 (0.030) | 0.907 (0.018) | 0.913 (0.022) | 0.980 (0.032) |
| Missouri | 75 | 0.957 (0.009) | 0.991 (0.018) | 0.985 (0.021) | 0.982 (0.021) | 0.991 (0.023) | 1.025 (0.019) |
| Montana | 11 | 0.921 (0.033) | 0.986 (0.031) | 1.039 (0.024) | 1.017 (0.042) | 1.062 (0.022) | 1.058 (0.034) |
| Nebraska | 24 | 0.940 (0.038) | 0.988 (0.035) | 0.998 (0.032) | 1.004 (0.037) | 1.012 (0.026) | 1.031 (0.016) |
| Nevada | 26 | 0.921 (0.019) | 0.988 (0.012) | 0.982 (0.023) | 0.970 (0.028) | 0.988 (0.029) | 1.024 (0.029) |
| New Hampshire | 15 | 0.908 (0.034) | 0.955 (0.019) | 1.017 (0.032) | 1.040 (0.029) | 1.055 (0.019) | 1.060 (0.038) |
| New Jersey | 116 | 0.957 (0.018) | 0.985 (0.017) | 1.001 (0.017) | 0.992 (0.024) | 1.021 (0.012) | 1.036 (0.019) |
| New Mexico | 27 | 0.958 (0.022) | 0.983 (0.028) | 0.983 (0.027) | 0.981 (0.027) | 1.002 (0.026) | 1.010 (0.024) |
| New York | 271 | 0.965 (0.009) | 0.989 (0.013) | 1.002 (0.014) | 0.994 (0.019) | 1.019 (0.011) | 1.032 (0.012) |
| North Carolina | 107 | 0.982 (0.017) | 0.990 (0.018) | 0.979 (0.015) | 0.958 (0.014) | 0.965 (0.017) | 1.000 (0.016) |
| North Dakota | 8 | 0.941 (0.031) | 0.999 (0.051) | 1.022 (0.050) | 1.036 (0.044) | 1.004 (0.049) | 1.026 (0.029) |
| Ohio | 156 | 0.942 (0.015) | 0.992 (0.009) | 1.009 (0.014) | 0.997 (0.020) | 1.010 (0.020) | 1.032 (0.021) |
| Oklahoma | 48 | 0.967 (0.013) | 0.990 (0.019) | 0.976 (0.022) | 0.972 (0.017) | 0.967 (0.022) | 1.011 (0.027) |
| Oregon | 43 | 0.938 (0.023) | 0.977 (0.013) | 1.002 (0.012) | 1.020 (0.029) | 1.049 (0.020) | 1.041 (0.014) |
| Pennsylvania | 153 | 0.952 (0.013) | 0.997 (0.018) | 1.003 (0.012) | 1.002 (0.020) | 1.020 (0.015) | 1.028 (0.014) |
| Rhode Island | 13 | 0.962 (0.030) | 0.985 (0.019) | 1.012 (0.020) | 1.030 (0.065) | 1.036 (0.031) | 1.036 (0.027) |
| South Carolina | 54 | 0.985 (0.020) | 0.997 (0.017) | 0.980 (0.020) | 0.949 (0.025) | 0.955 (0.011) | 0.989 (0.018) |
| South Dakota | 11 | 0.927 (0.033) | 0.978 (0.041) | 1.009 (0.045) | 1.017 (0.037) | 1.043 (0.037) | 1.034 (0.034) |
| Tennessee | 75 | 0.960 (0.024) | 0.989 (0.014) | 0.986 (0.023) | 0.962 (0.016) | 0.975 (0.020) | 1.010 (0.017) |
| Texas | 332 | 0.962 (0.012) | 0.966 (0.014) | 0.951 (0.016) | 0.943 (0.016) | 0.956 (0.017) | 0.989 (0.017) |
| Utah | 41 | 0.908 (0.024) | 0.977 (0.016) | 1.006 (0.016) | 1.037 (0.021) | 1.046 (0.012) | 1.066 (0.026) |
| Vermont | 7 | 0.915 (0.058) | 0.956 (0.070) | 1.014 (0.044) | 1.058 (0.062) | 1.070 (0.041) | 1.103 (0.038) |
| Virginia | 95 | 0.956 (0.016) | 0.989 (0.021) | 1.001 (0.020) | 0.986 (0.019) | 0.998 (0.019) | 1.014 (0.015) |
| Washington | 79 | 0.936 (0.011) | 0.975 (0.021) | 1.011 (0.015) | 1.023 (0.016) | 1.044 (0.022) | 1.049 (0.011) |
| West Virginia | 21 | 0.959 (0.020) | 0.988 (0.027) | 0.991 (0.028) | 0.976 (0.020) | 0.989 (0.018) | 1.012 (0.031) |
| Wisconsin | 69 | 0.929 (0.018) | 0.980 (0.013) | 1.019 (0.021) | 1.023 (0.026) | 1.039 (0.019) | 1.044 (0.022) |
| Wyoming | 6 | 0.926 (0.026) | 0.976 (0.038) | 1.040 (0.051) | 1.044 (0.068) | 1.036 (0.044) | 1.054 (0.051) |
|  |  |  |  |  |  |  |  |
| **State** | **N** | **July** | **August** | **September** | **October** | **November** | **December** |
| Alabama | 62 | 1.040 (0.017) | 1.054 (0.026) | 1.071 (0.018) | 1.000 (0.025) | 0.979 (0.020) | 1.010 (0.024) |
| Alaska | 11 | 1.032 (0.047) | 1.024 (0.034) | 1.053 (0.023) | 0.979 (0.022) | 0.923 (0.048) | 0.971 (0.033) |
| Arizona | 74 | 1.023 (0.014) | 1.063 (0.022) | 1.090 (0.018) | 1.016 (0.014) | 1.006 (0.022) | 1.014 (0.012) |
| Arkansas | 36 | 1.051 (0.029) | 1.061 (0.032) | 1.059 (0.024) | 0.996 (0.032) | 0.995 (0.031) | 1.022 (0.022) |
| California | 555 | 1.023 (0.016) | 1.041 (0.014) | 1.063 (0.016) | 1.009 (0.011) | 0.987 (0.019) | 0.981 (0.020) |
| Colorado | 57 | 1.040 (0.017) | 1.031 (0.024) | 1.038 (0.022) | 0.987 (0.018) | 0.956 (0.025) | 0.957 (0.021) |
| Connecticut | 45 | 1.050 (0.024) | 1.030 (0.030) | 1.043 (0.015) | 0.978 (0.023) | 0.935 (0.028) | 0.932 (0.016) |
| Delaware | 11 | 1.036 (0.040) | 1.045 (0.035) | 1.022 (0.035) | 0.983 (0.027) | 0.947 (0.031) | 0.976 (0.020) |
| District of Columbia | 9 | 1.035 (0.027) | 1.043 (0.043) | 1.010 (0.021) | 0.986 (0.041) | 0.967 (0.052) | 0.981 (0.056) |
| Florida | 194 | 1.003 (0.021) | 1.040 (0.016) | 1.074 (0.013) | 1.028 (0.015) | 1.013 (0.019) | 1.031 (0.022) |
| Georgia | 117 | 1.028 (0.009) | 1.049 (0.018) | 1.065 (0.014) | 1.003 (0.017) | 0.995 (0.014) | 1.020 (0.021) |
| Hawaii | 19 | 0.989 (0.025) | 0.999 (0.024) | 1.059 (0.021) | 1.014 (0.020) | 0.993 (0.025) | 1.013 (0.043) |
| Idaho | 18 | 1.049 (0.032) | 1.030 (0.040) | 1.051 (0.025) | 0.968 (0.033) | 0.948 (0.023) | 0.948 (0.033) |
| Illinois | 187 | 1.052 (0.013) | 1.046 (0.019) | 1.043 (0.016) | 0.993 (0.017) | 0.961 (0.020) | 0.965 (0.013) |
| Indiana | 85 | 1.049 (0.014) | 1.046 (0.018) | 1.058 (0.015) | 0.984 (0.013) | 0.955 (0.018) | 0.975 (0.019) |
| Iowa | 38 | 1.046 (0.021) | 1.027 (0.017) | 1.052 (0.030) | 0.985 (0.024) | 0.964 (0.021) | 0.969 (0.020) |
| Kansas | 38 | 1.043 (0.023) | 1.032 (0.016) | 1.054 (0.020) | 0.988 (0.021) | 0.960 (0.031) | 0.988 (0.022) |
| Kentucky | 54 | 1.048 (0.019) | 1.046 (0.022) | 1.063 (0.009) | 1.002 (0.012) | 0.966 (0.023) | 0.991 (0.025) |
| Louisiana | 68 | 1.033 (0.023) | 1.070 (0.029) | 1.095 (0.021) | 1.021 (0.018) | 1.018 (0.018) | 1.028 (0.028) |
| Maine | 14 | 1.050 (0.040) | 1.034 (0.019) | 1.032 (0.031) | 0.965 (0.023) | 0.911 (0.030) | 0.912 (0.024) |
| Maryland | 74 | 1.038 (0.017) | 1.036 (0.019) | 1.047 (0.022) | 0.988 (0.011) | 0.954 (0.018) | 0.971 (0.021) |
| Massachusetts | 83 | 1.052 (0.016) | 1.030 (0.016) | 1.033 (0.017) | 0.981 (0.018) | 0.939 (0.015) | 0.938 (0.020) |
| Michigan | 138 | 1.043 (0.017) | 1.032 (0.016) | 1.026 (0.013) | 0.969 (0.014) | 0.935 (0.017) | 0.942 (0.018) |
| Minnesota | 65 | 1.045 (0.012) | 1.033 (0.017) | 1.033 (0.009) | 0.983 (0.016) | 0.941 (0.022) | 0.940 (0.020) |
| Mississippi | 42 | 1.055 (0.029) | 1.078 (0.026) | 1.088 (0.021) | 1.012 (0.023) | 1.009 (0.017) | 1.038 (0.034) |
| Missouri | 75 | 1.044 (0.017) | 1.040 (0.020) | 1.059 (0.023) | 0.988 (0.020) | 0.965 (0.012) | 0.974 (0.017) |
| Montana | 11 | 1.045 (0.036) | 1.023 (0.036) | 1.020 (0.032) | 0.942 (0.050) | 0.942 (0.029) | 0.945 (0.039) |
| Nebraska | 24 | 1.041 (0.025) | 1.032 (0.023) | 1.030 (0.035) | 0.983 (0.034) | 0.970 (0.026) | 0.972 (0.035) |
| Nevada | 26 | 1.033 (0.023) | 1.045 (0.030) | 1.050 (0.031) | 1.009 (0.034) | 0.984 (0.024) | 1.006 (0.028) |
| New Hampshire | 15 | 1.075 (0.026) | 1.044 (0.033) | 1.029 (0.034) | 0.973 (0.022) | 0.927 (0.028) | 0.914 (0.038) |
| New Jersey | 116 | 1.054 (0.015) | 1.028 (0.016) | 1.038 (0.019) | 0.984 (0.022) | 0.954 (0.020) | 0.948 (0.020) |
| New Mexico | 27 | 1.042 (0.022) | 1.043 (0.035) | 1.050 (0.025) | 0.999 (0.022) | 0.973 (0.031) | 0.975 (0.026) |
| New York | 271 | 1.042 (0.010) | 1.025 (0.012) | 1.031 (0.008) | 0.984 (0.013) | 0.955 (0.014) | 0.963 (0.017) |
| North Carolina | 107 | 1.032 (0.017) | 1.047 (0.019) | 1.061 (0.020) | 1.001 (0.017) | 0.979 (0.015) | 1.004 (0.011) |
| North Dakota | 8 | 1.048 (0.035) | 1.038 (0.038) | 1.038 (0.031) | 0.955 (0.028) | 0.936 (0.039) | 0.959 (0.047) |
| Ohio | 156 | 1.049 (0.012) | 1.039 (0.020) | 1.042 (0.017) | 0.983 (0.017) | 0.950 (0.016) | 0.956 (0.012) |
| Oklahoma | 48 | 1.044 (0.027) | 1.046 (0.025) | 1.063 (0.026) | 1.001 (0.026) | 0.966 (0.022) | 0.996 (0.030) |
| Oregon | 43 | 1.036 (0.023) | 1.025 (0.018) | 1.030 (0.020) | 0.966 (0.015) | 0.954 (0.020) | 0.962 (0.019) |
| Pennsylvania | 153 | 1.045 (0.010) | 1.035 (0.015) | 1.041 (0.013) | 0.982 (0.014) | 0.951 (0.016) | 0.946 (0.016) |
| Rhode Island | 13 | 1.055 (0.031) | 1.022 (0.039) | 1.023 (0.033) | 0.984 (0.045) | 0.927 (0.032) | 0.927 (0.017) |
| South Carolina | 54 | 1.024 (0.026) | 1.048 (0.026) | 1.057 (0.011) | 1.003 (0.014) | 0.992 (0.024) | 1.022 (0.029) |
| South Dakota | 11 | 1.031 (0.025) | 1.032 (0.040) | 1.042 (0.043) | 0.977 (0.052) | 0.936 (0.034) | 0.973 (0.040) |
| Tennessee | 75 | 1.044 (0.017) | 1.044 (0.023) | 1.061 (0.018) | 0.987 (0.014) | 0.979 (0.021) | 1.002 (0.016) |
| Texas | 332 | 1.035 (0.013) | 1.062 (0.017) | 1.079 (0.011) | 1.027 (0.018) | 1.007 (0.014) | 1.021 (0.017) |
| Utah | 41 | 1.046 (0.026) | 1.016 (0.033) | 1.024 (0.019) | 0.975 (0.022) | 0.944 (0.028) | 0.956 (0.025) |
| Vermont | 7 | 1.061 (0.053) | 1.024 (0.044) | 1.036 (0.036) | 0.940 (0.064) | 0.920 (0.048) | 0.903 (0.046) |
| Virginia | 95 | 1.032 (0.013) | 1.034 (0.016) | 1.059 (0.019) | 0.986 (0.020) | 0.966 (0.016) | 0.979 (0.017) |
| Washington | 79 | 1.046 (0.028) | 1.028 (0.016) | 1.030 (0.017) | 0.969 (0.018) | 0.938 (0.023) | 0.950 (0.017) |
| West Virginia | 21 | 1.047 (0.024) | 1.053 (0.031) | 1.055 (0.032) | 0.992 (0.015) | 0.956 (0.023) | 0.981 (0.038) |
| Wisconsin | 69 | 1.046 (0.023) | 1.024 (0.021) | 1.035 (0.017) | 0.971 (0.013) | 0.939 (0.015) | 0.949 (0.022) |
| Wyoming | 6 | 1.052 (0.055) | 1.003 (0.041) | 1.025 (0.045) | 0.969 (0.060) | 0.929 (0.051) | 0.946 (0.053) |

N indicates the mean number of births per year in thousands

For each month the mean and (standard deviation) are shown

Figures S3 and S4 show the distribution obtained by combining the data from all 824 European MOB data sets (16 countries) and all 520 North American MOB data sets (51 American regions and Canada).

Both these combined data sets show highly significant evidence for seasonality in the timing of birth. Even if these data are scaled they remain nominally significant down to a sample size of around 41000 in the case of the European data and 48000 in the case of the North American data. Note that the September blip in frequency is evident in both these continental level data sets.

In comparing the MOB distribution between populations we noted that for some months there was remarkable similarity between populations differing in latitude while for other months there were marked differences. These details are shown in the 12 panels of figure S5 below. We also noticed that these latitudinal effects seem to have reduced over time, see the 12 panels of figure S6.


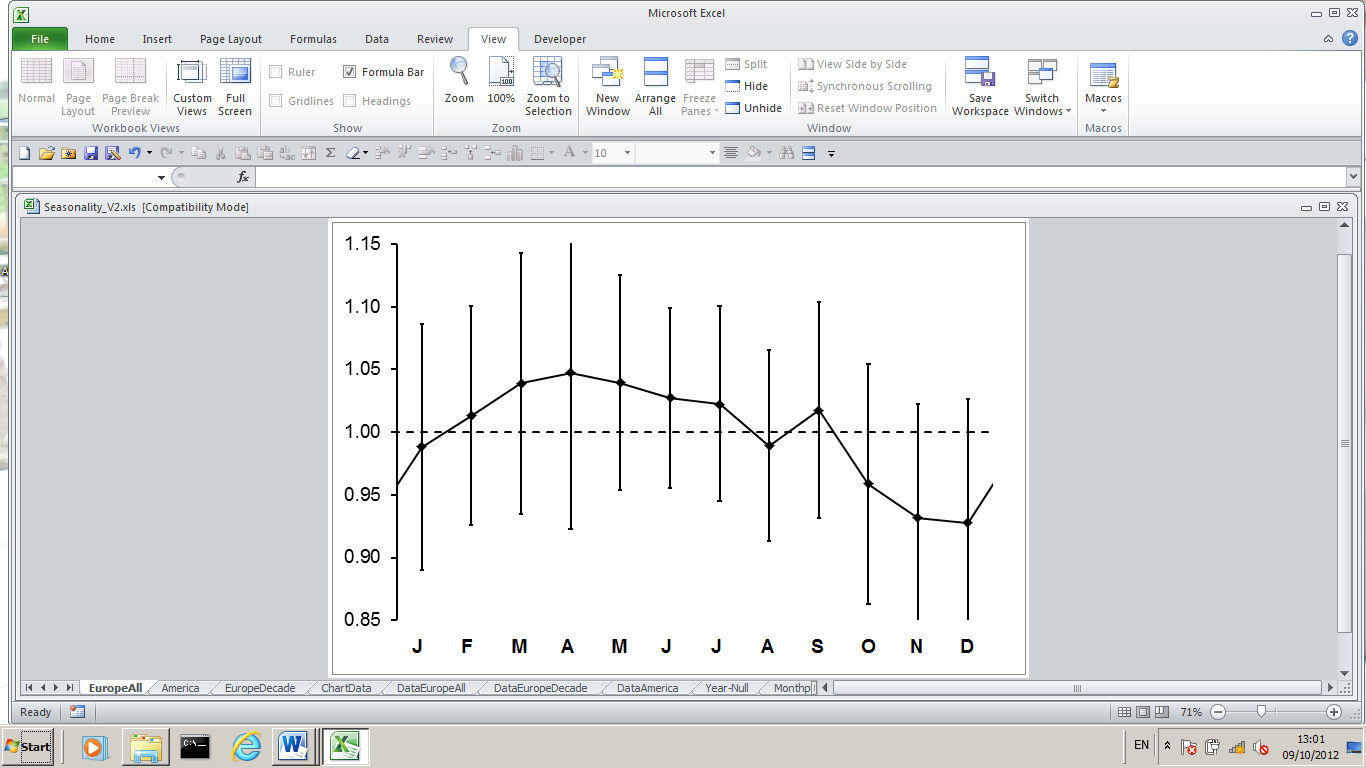


**Figure S3.** The mean (with 95% confidence intervals) for the average normalised daily birth rate in the 824 European MOB data sets (based on 271 million births).


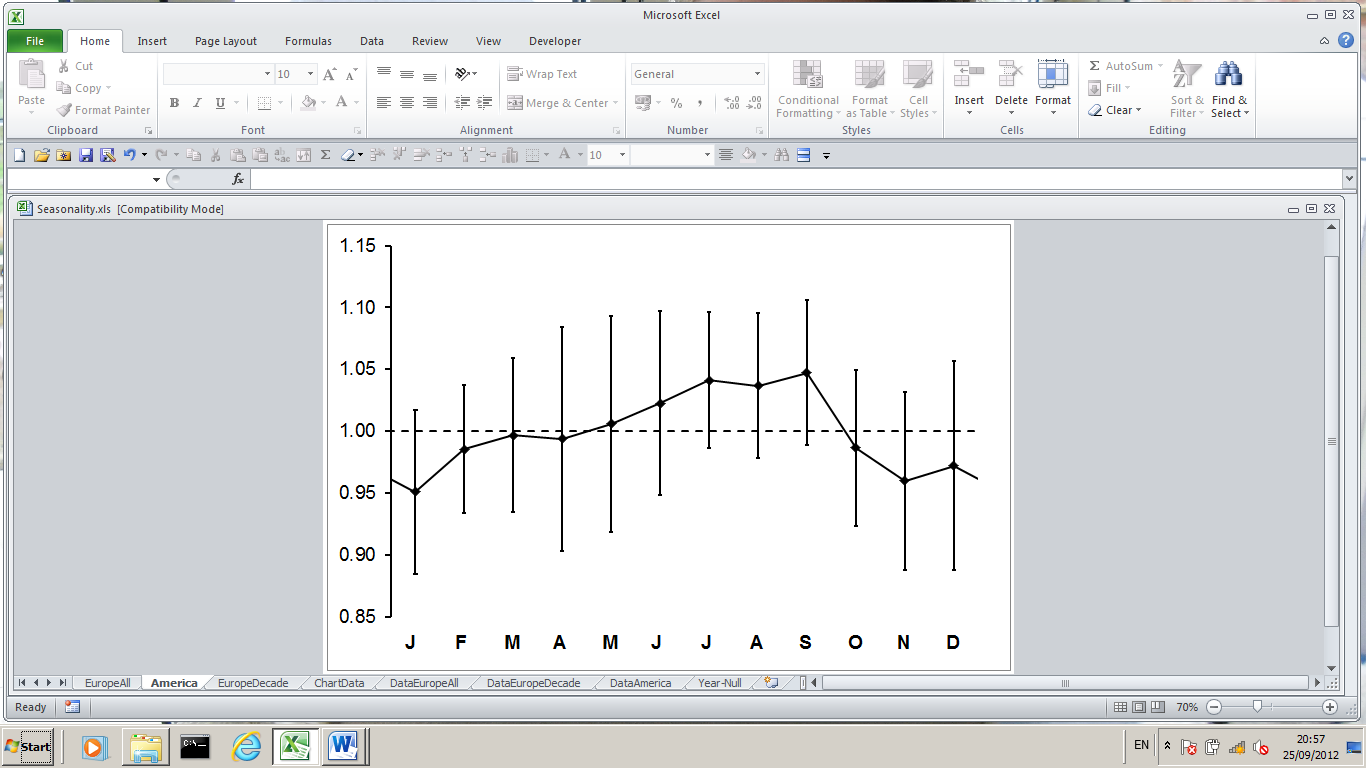


**Figure S4.** The mean (with 95% confidence intervals) for the average normalised daily birth rate in the 520 North American MOB data sets (based on 43 million births).


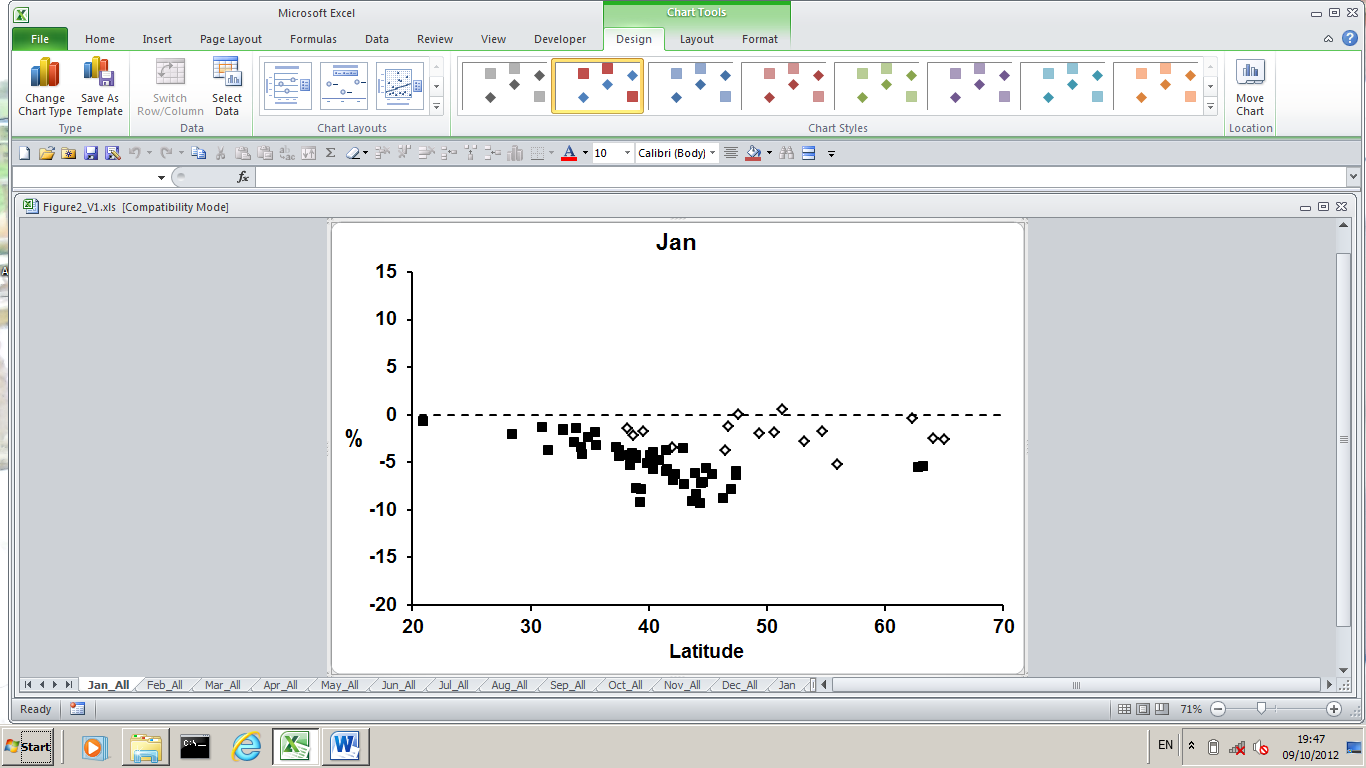


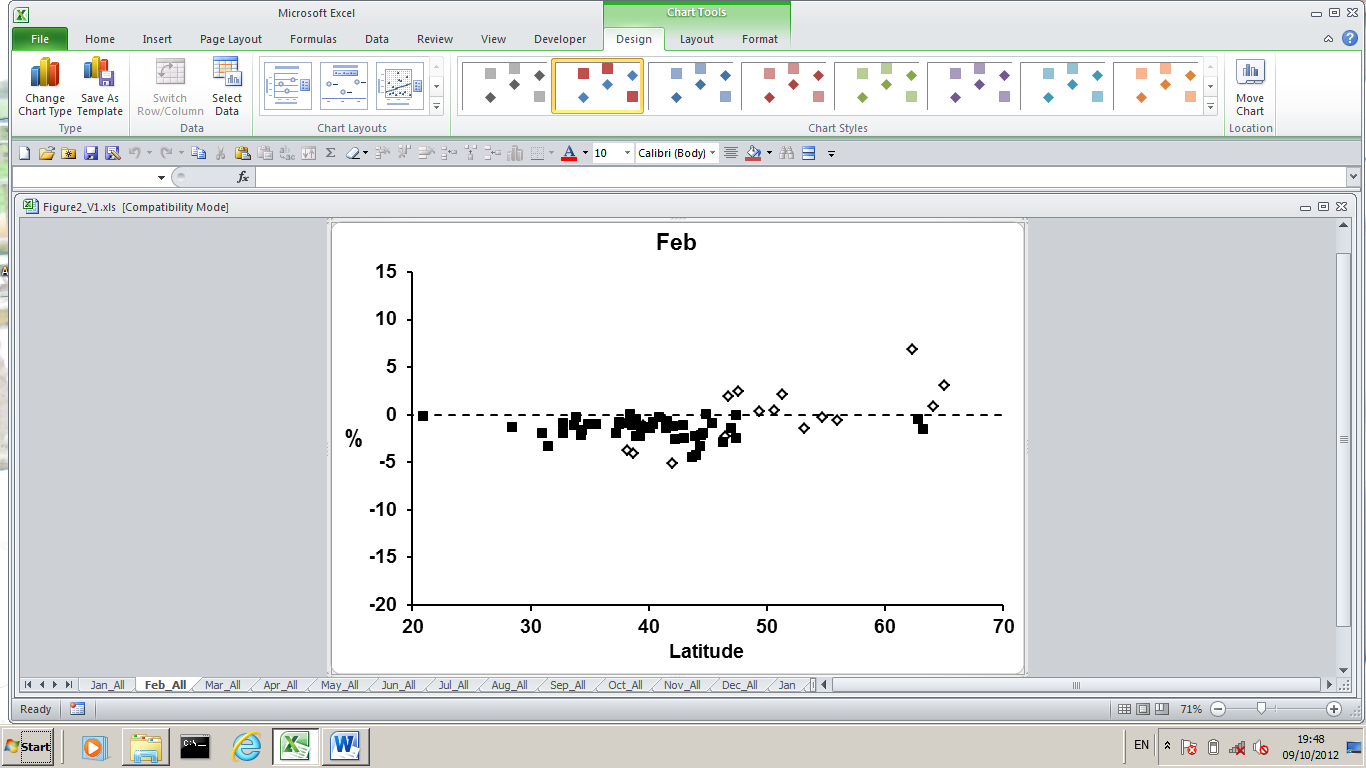


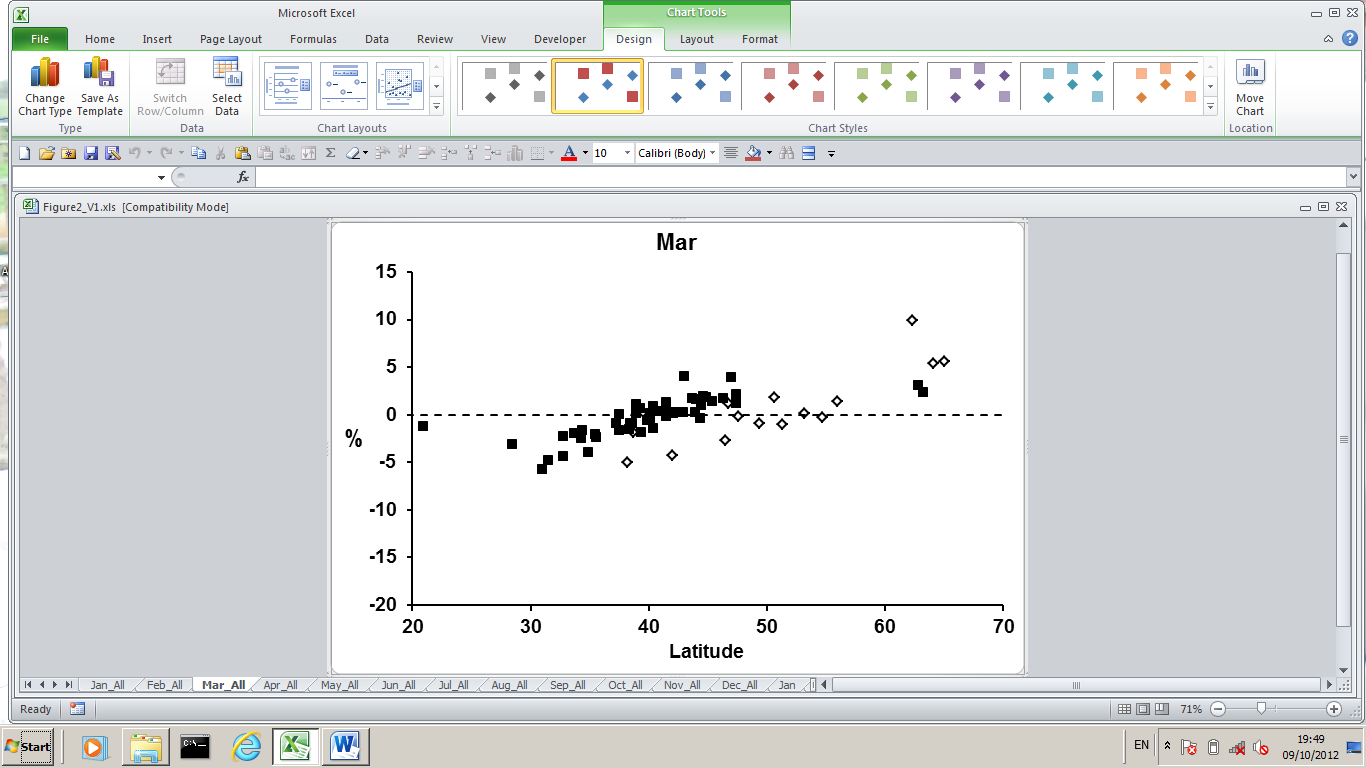


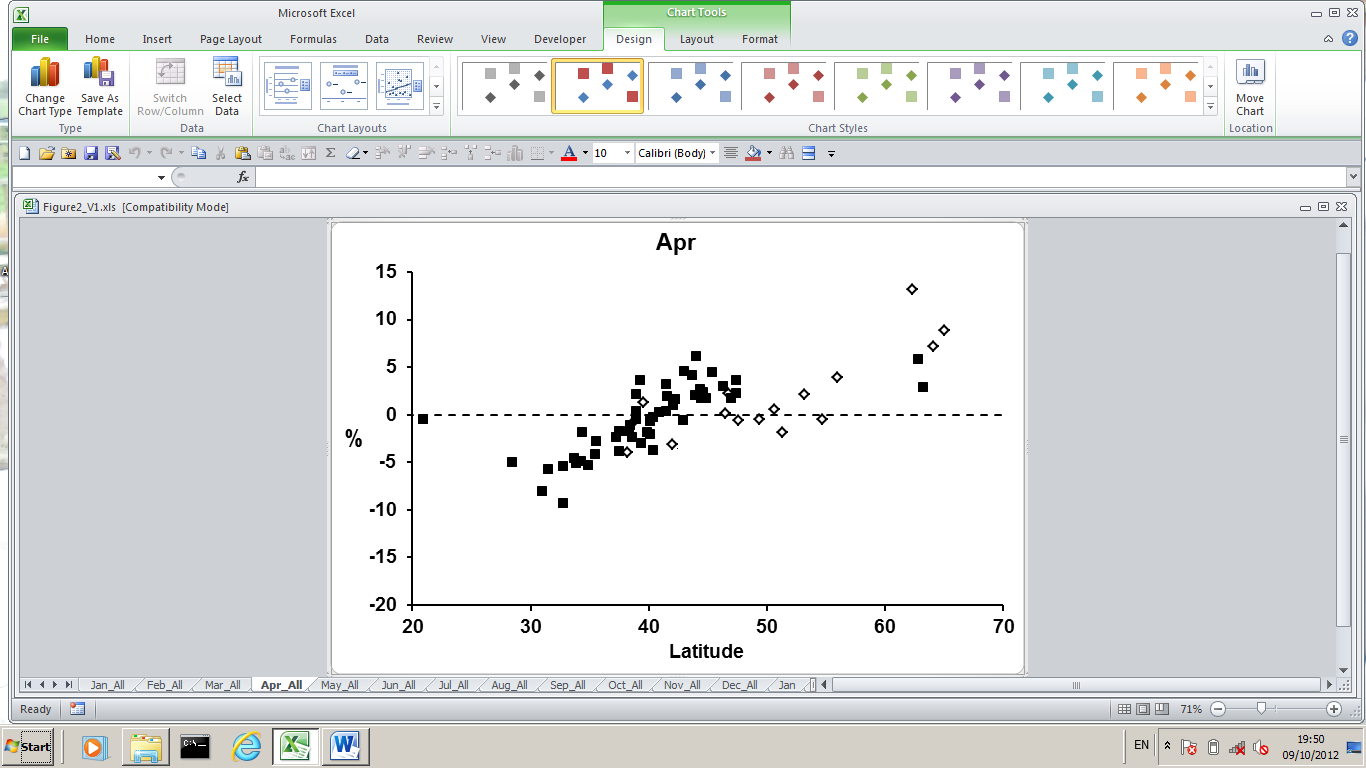


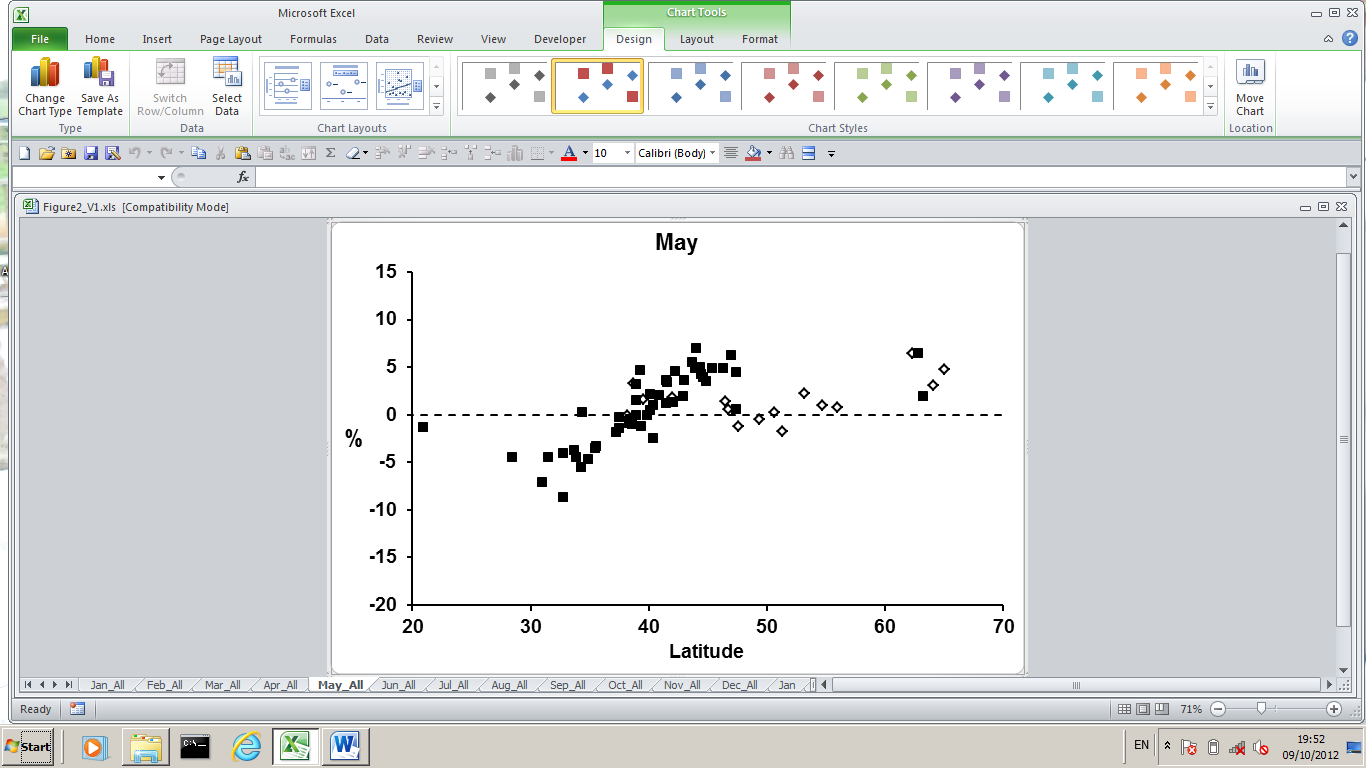


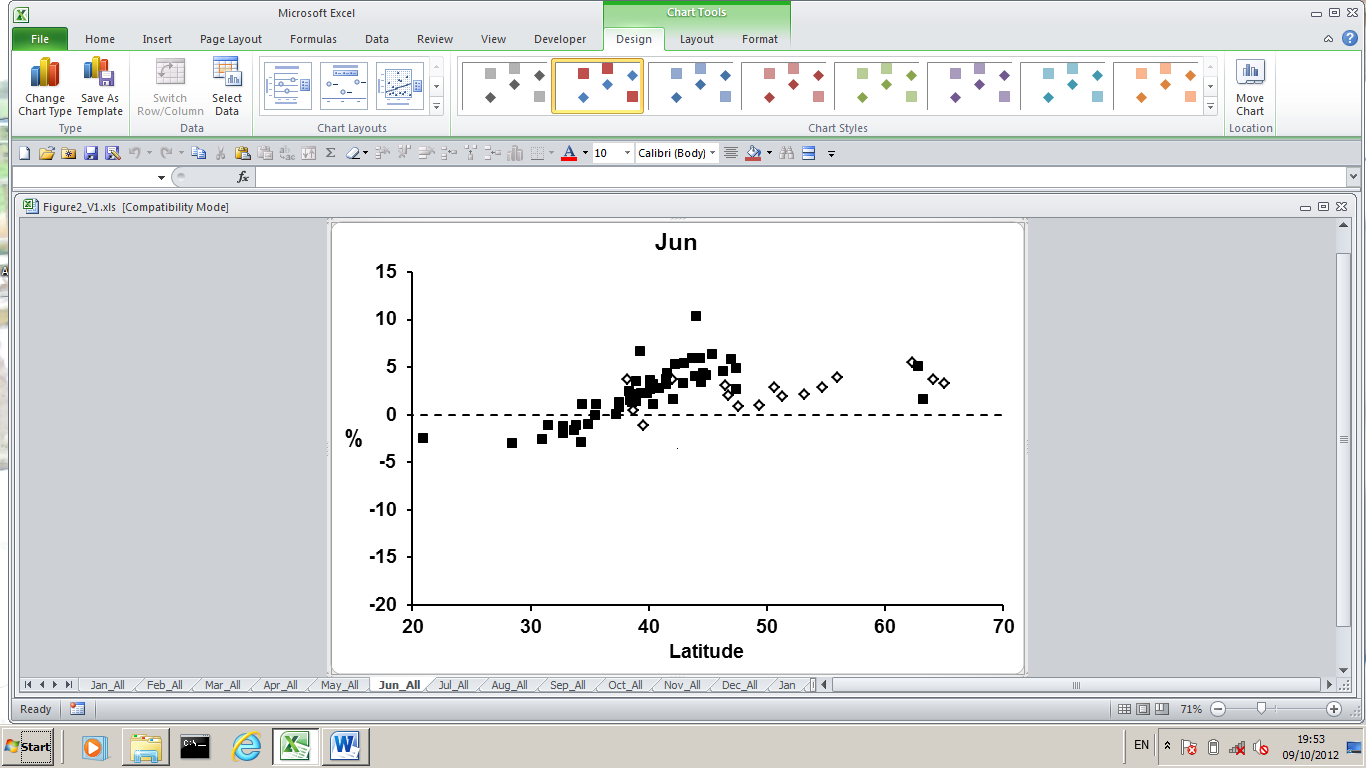


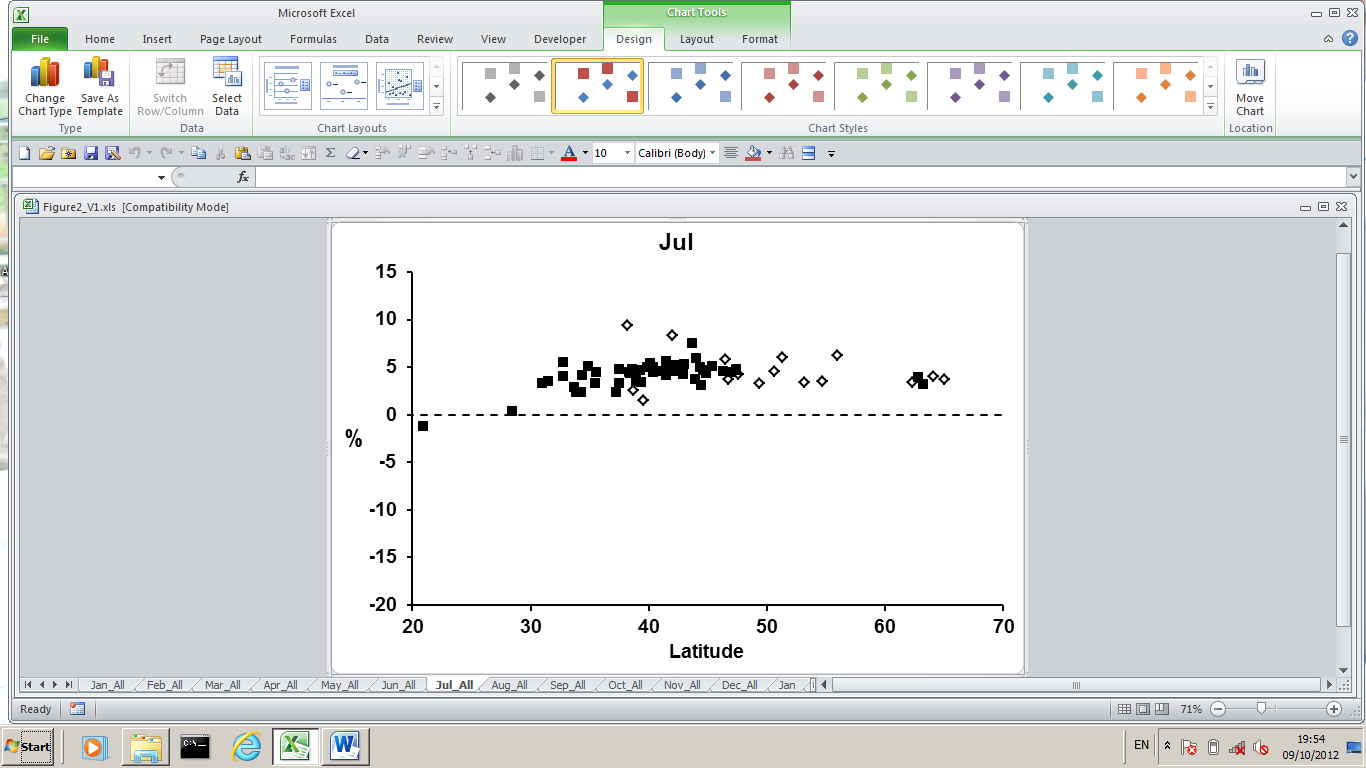


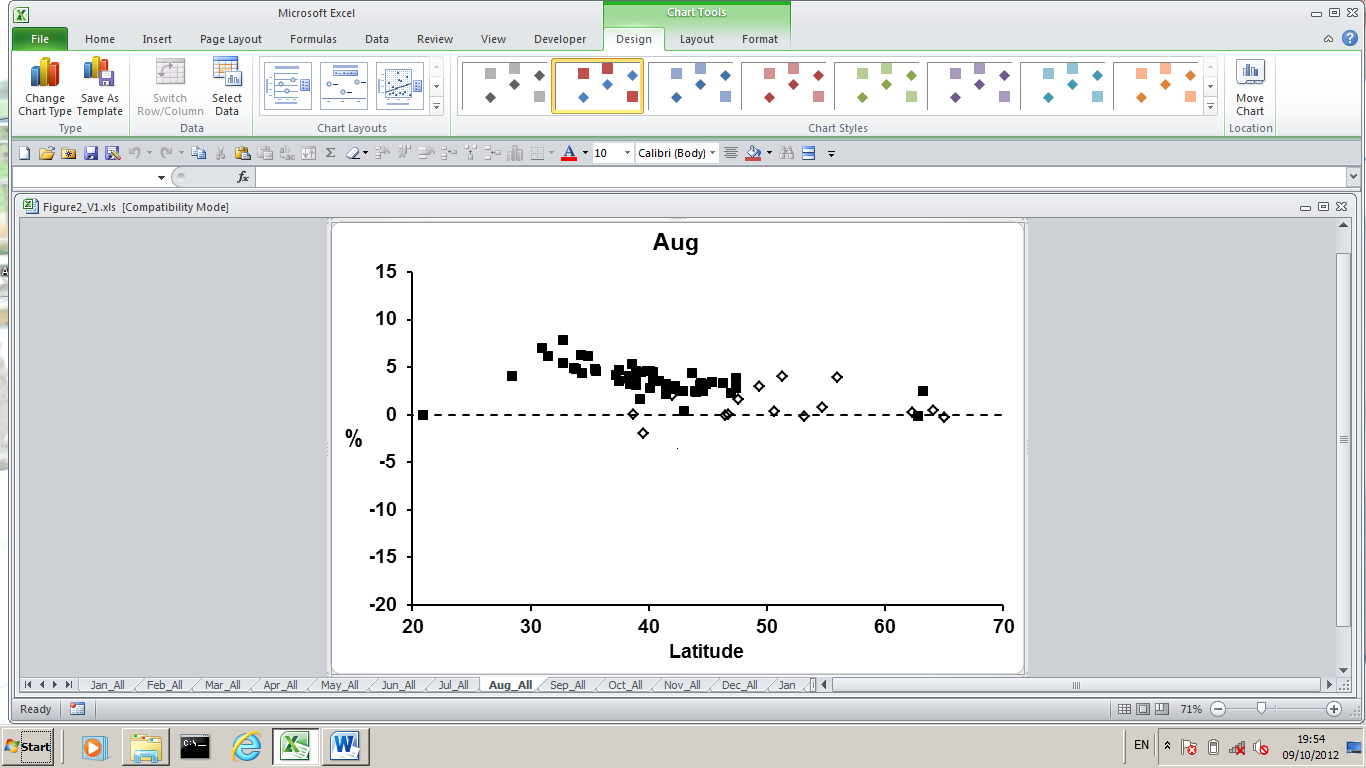


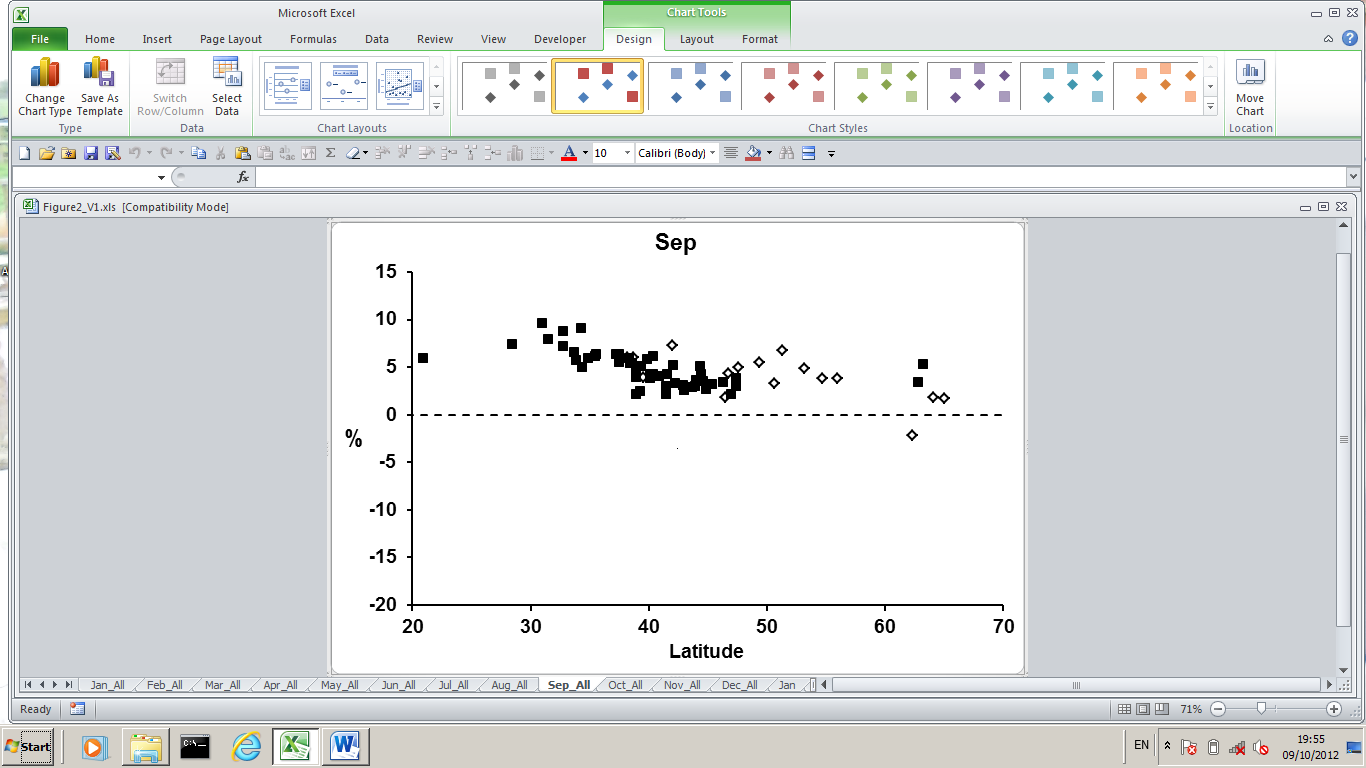


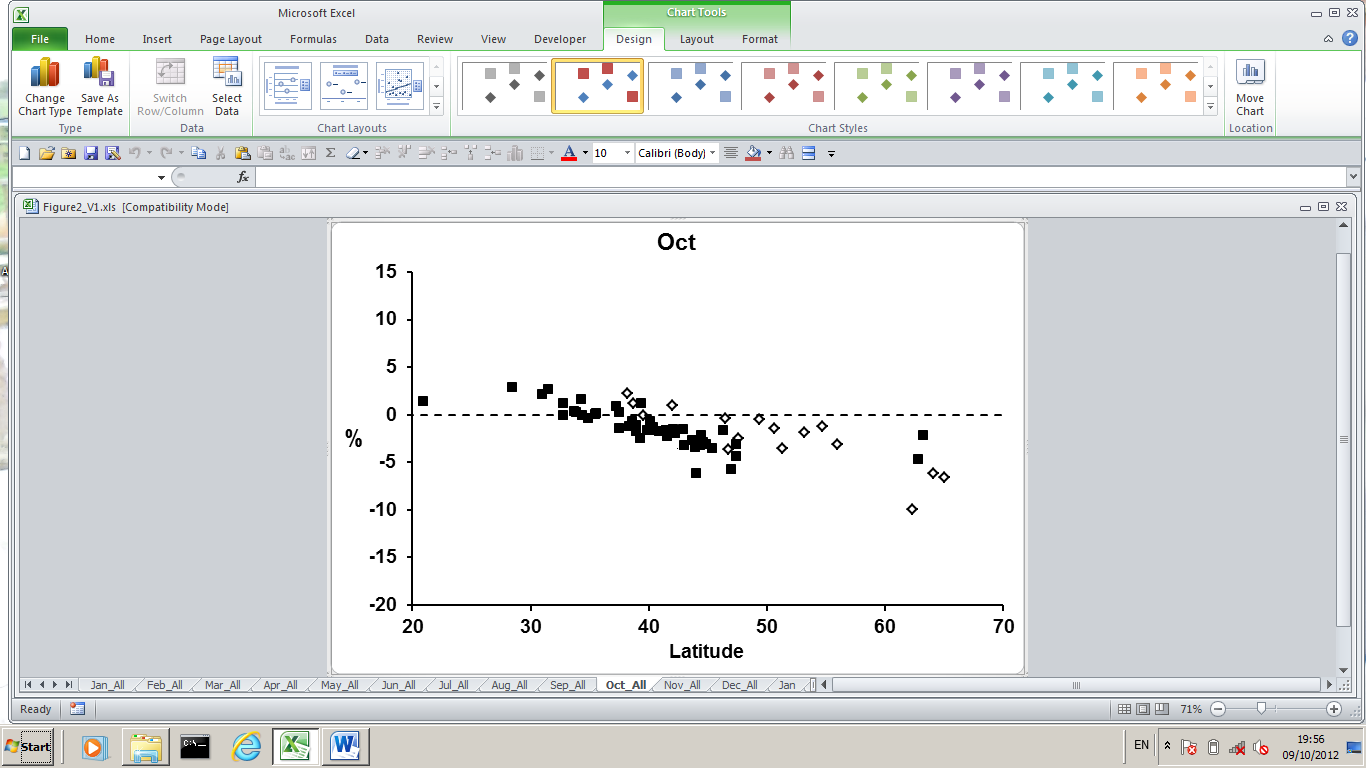


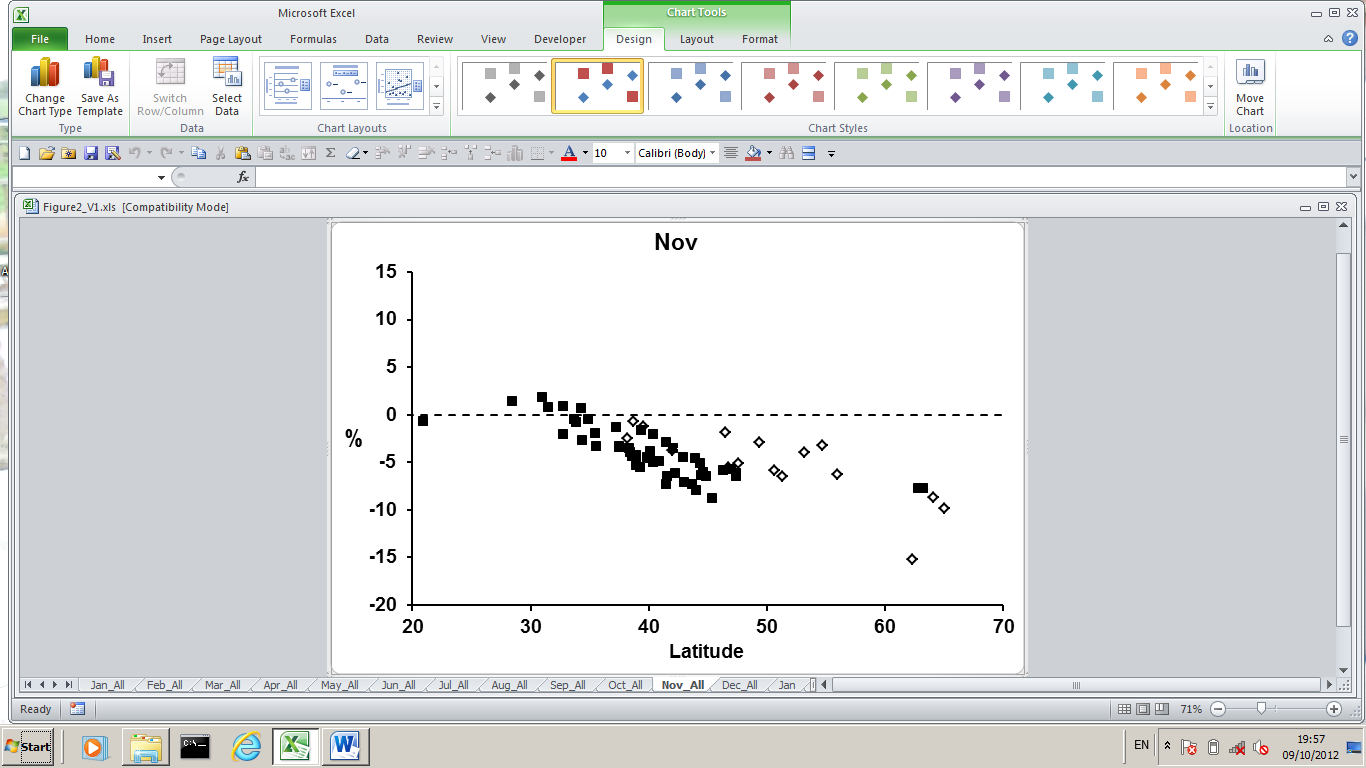


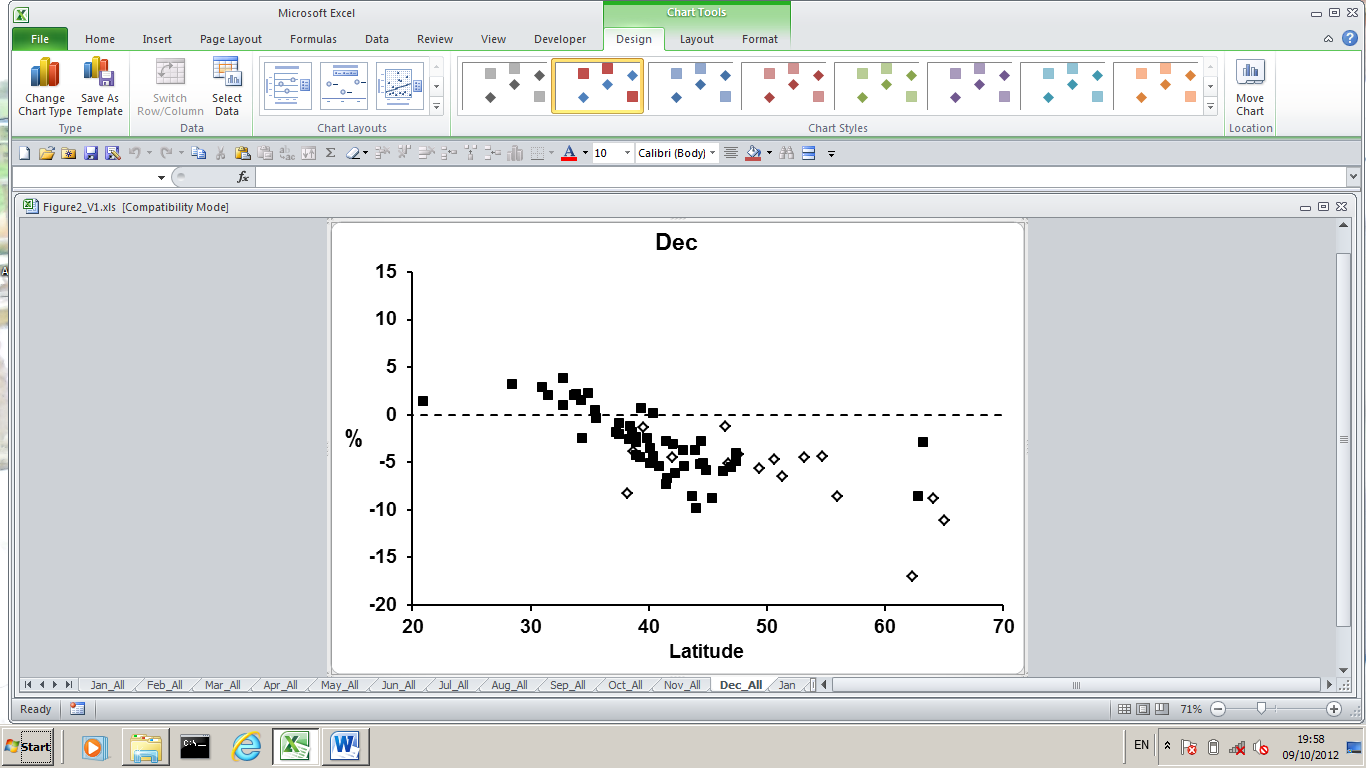


**Figure S5.** Correlation between excess average normalised daily birth rate and latitude in Europe (white rhomboids) and North America (black squares). Values are based on data from the 10 year period 1991-2000. Because the population of the District of Columbia is subject to marked swings in size as people migrate in and out of neighbouring states (Maryland and Virginia) the births in this small state were included in with those for Virginia and are not shown separately. For each state and country the latitude employed is the central point of the main land mass (i.e.mid way between the Northern and Southern borders).


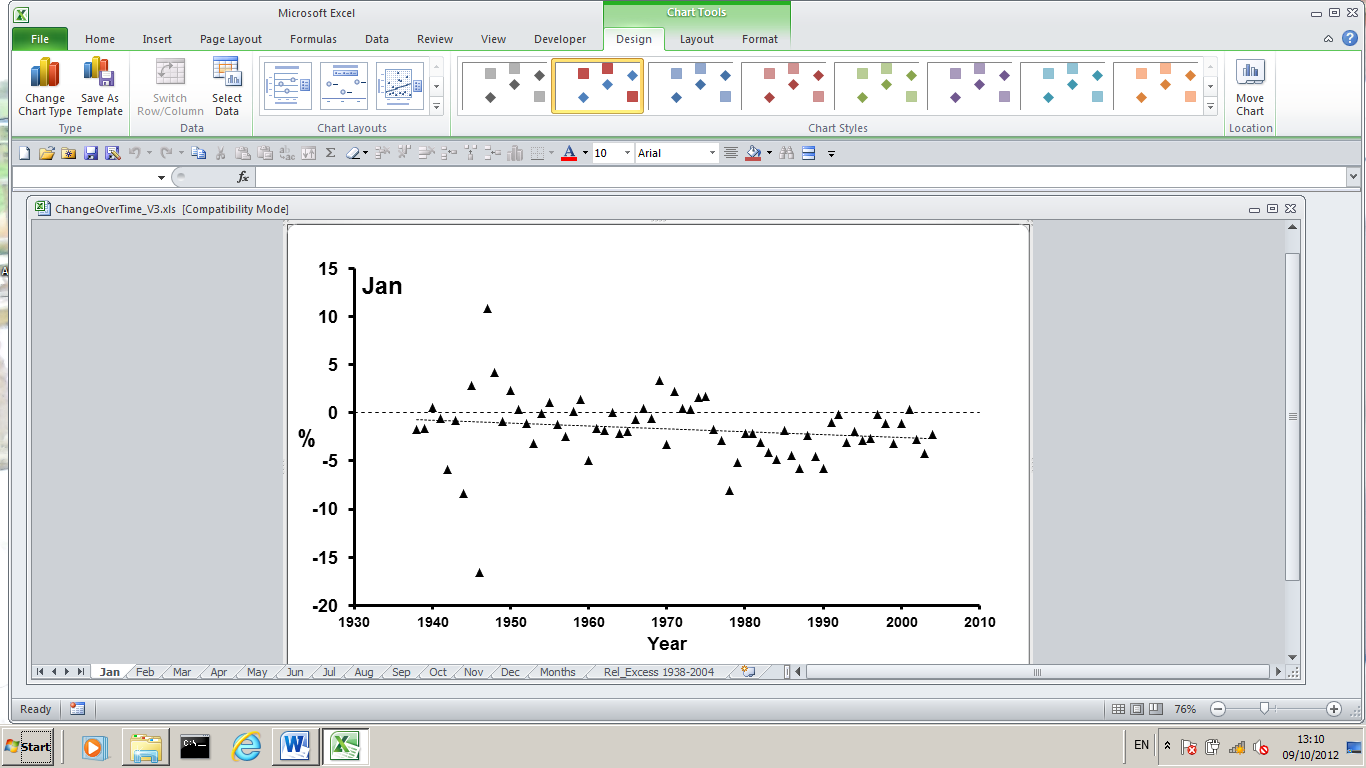


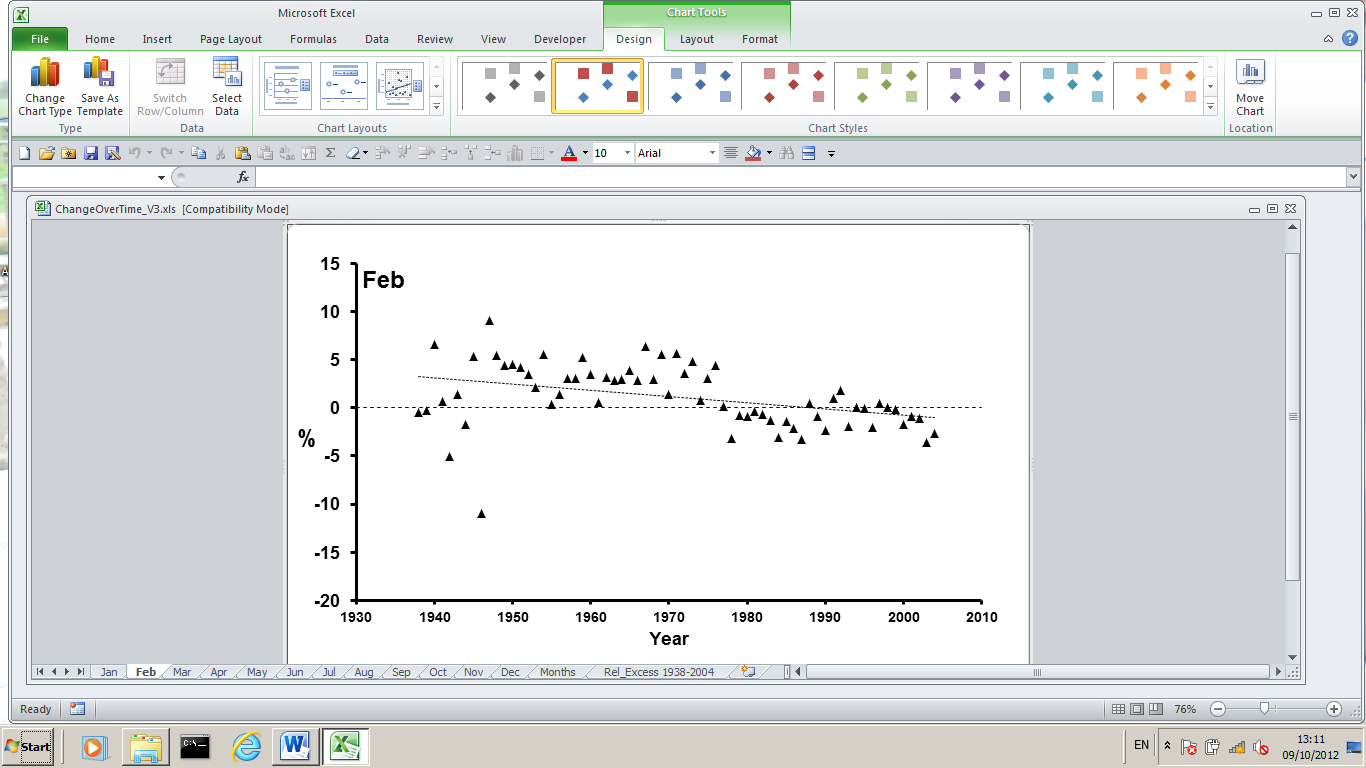


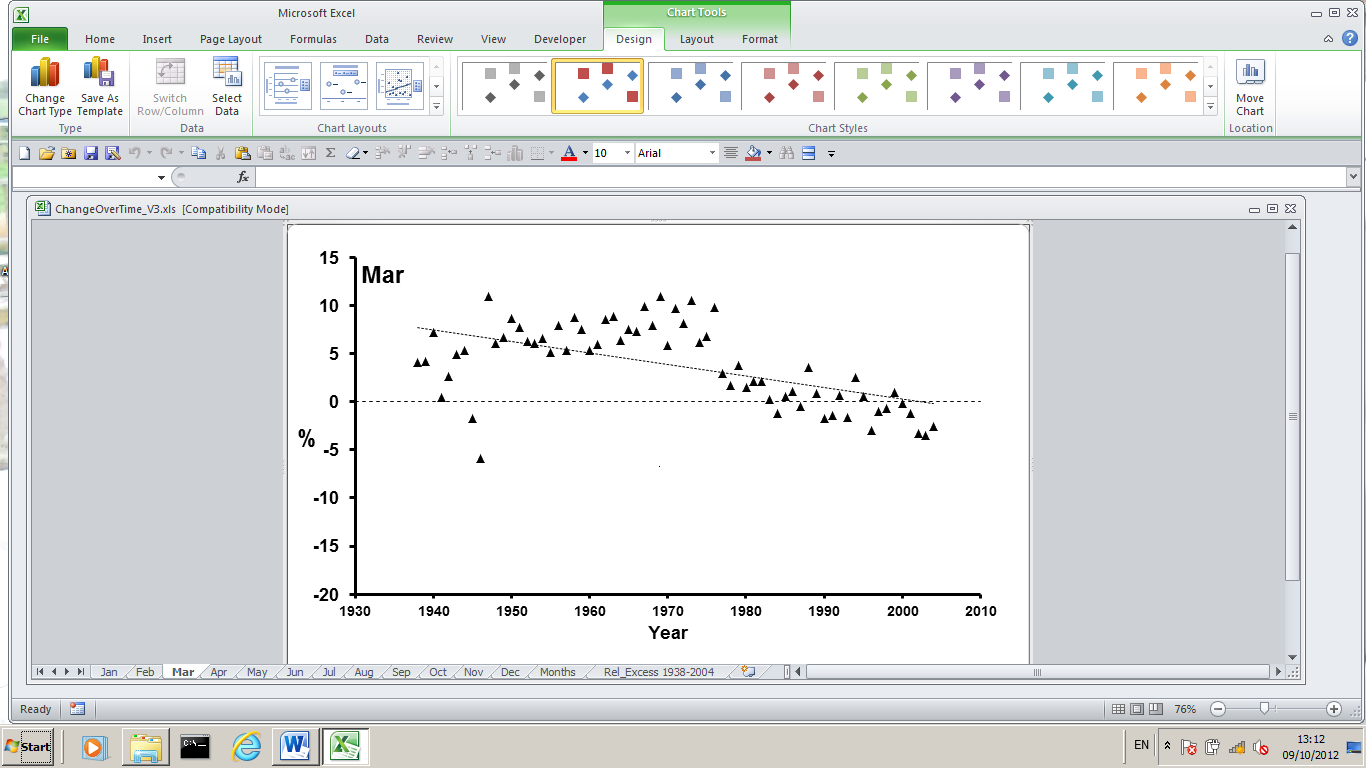


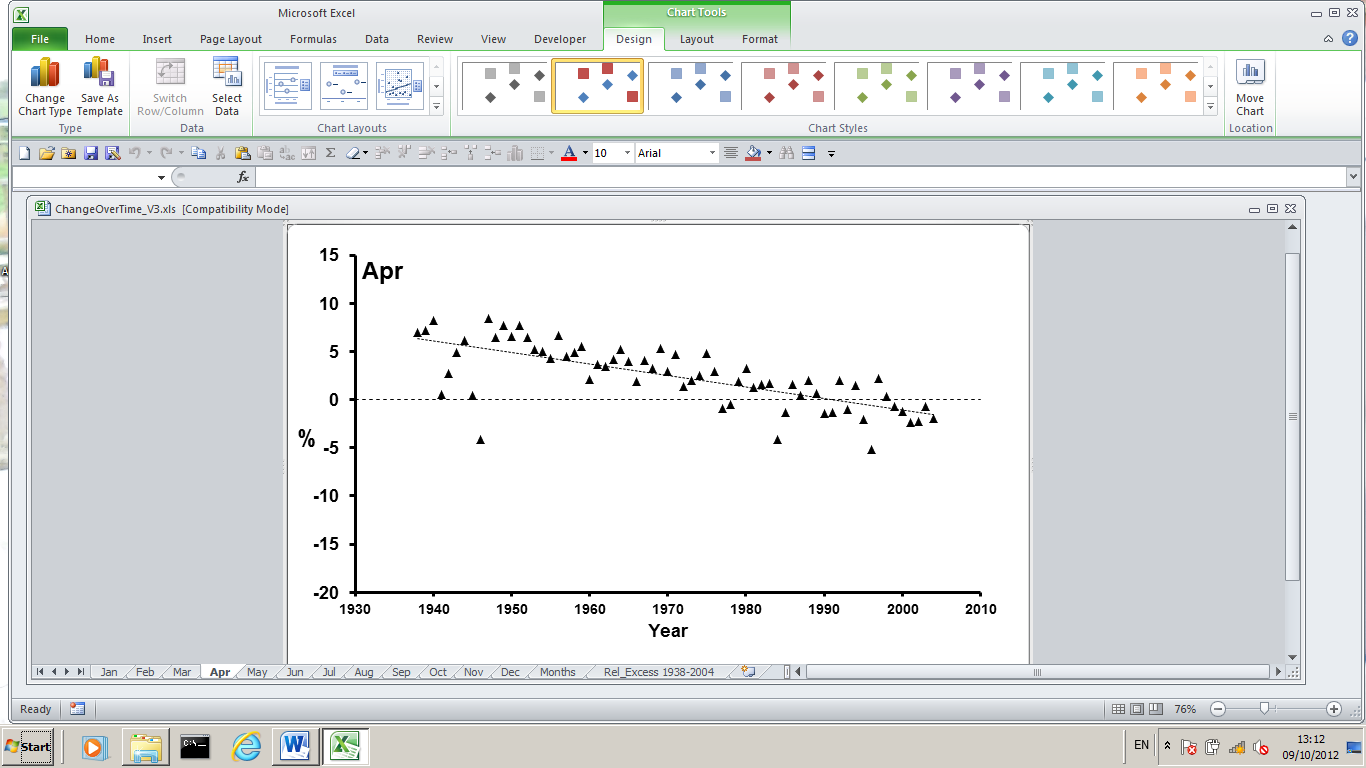


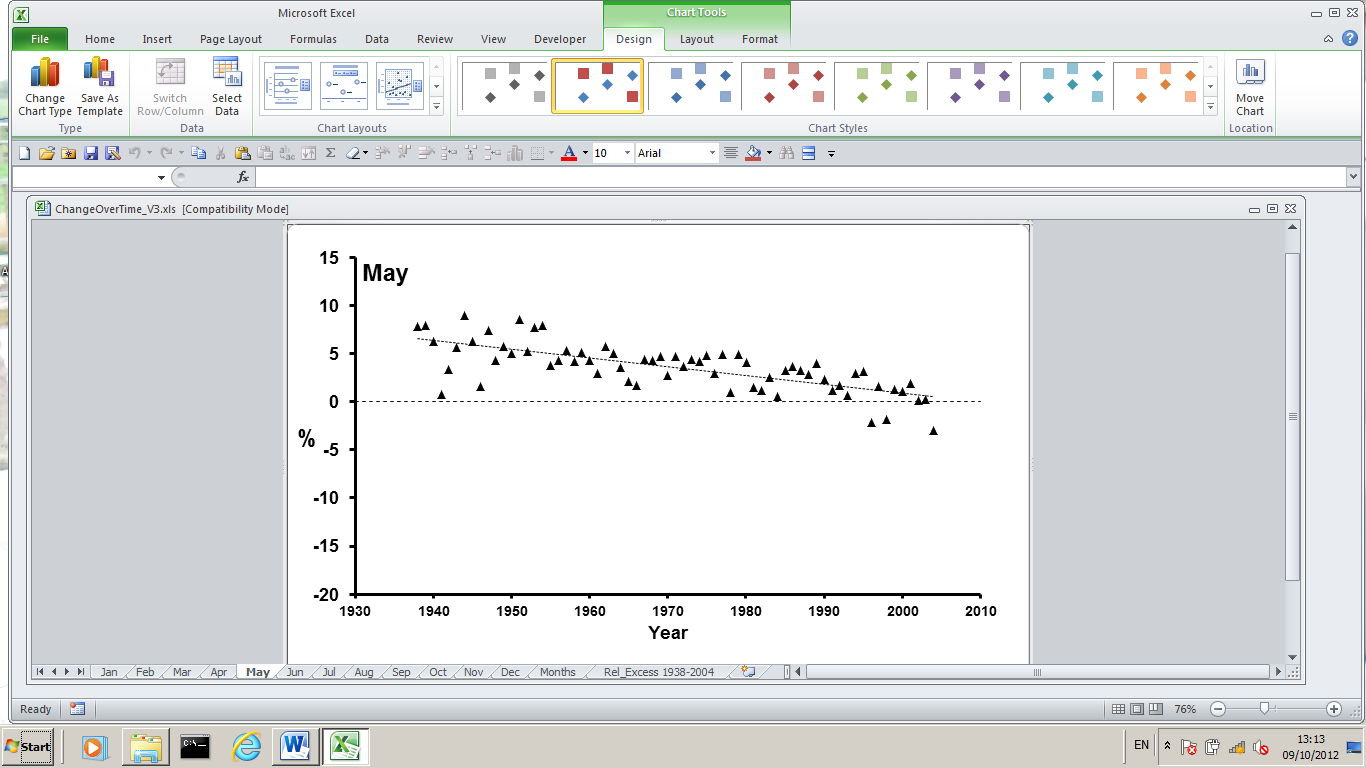


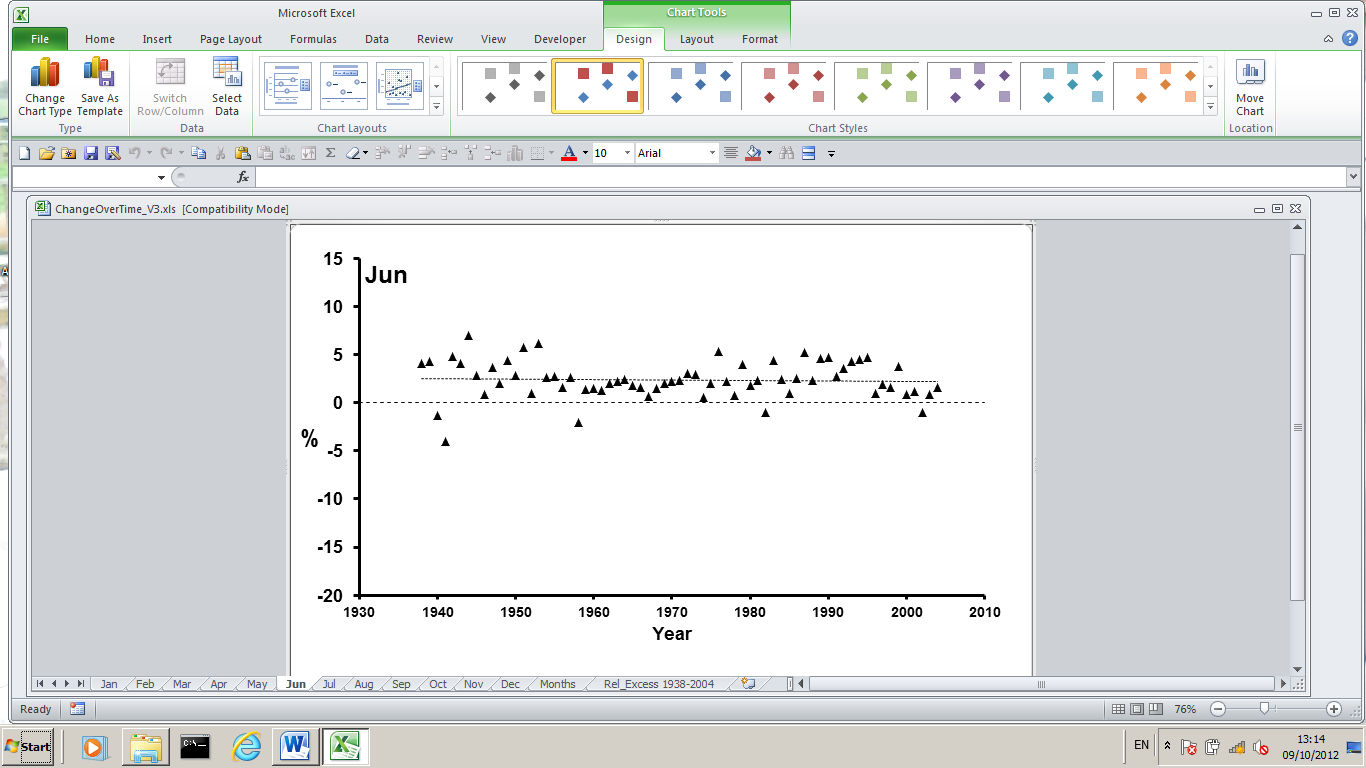


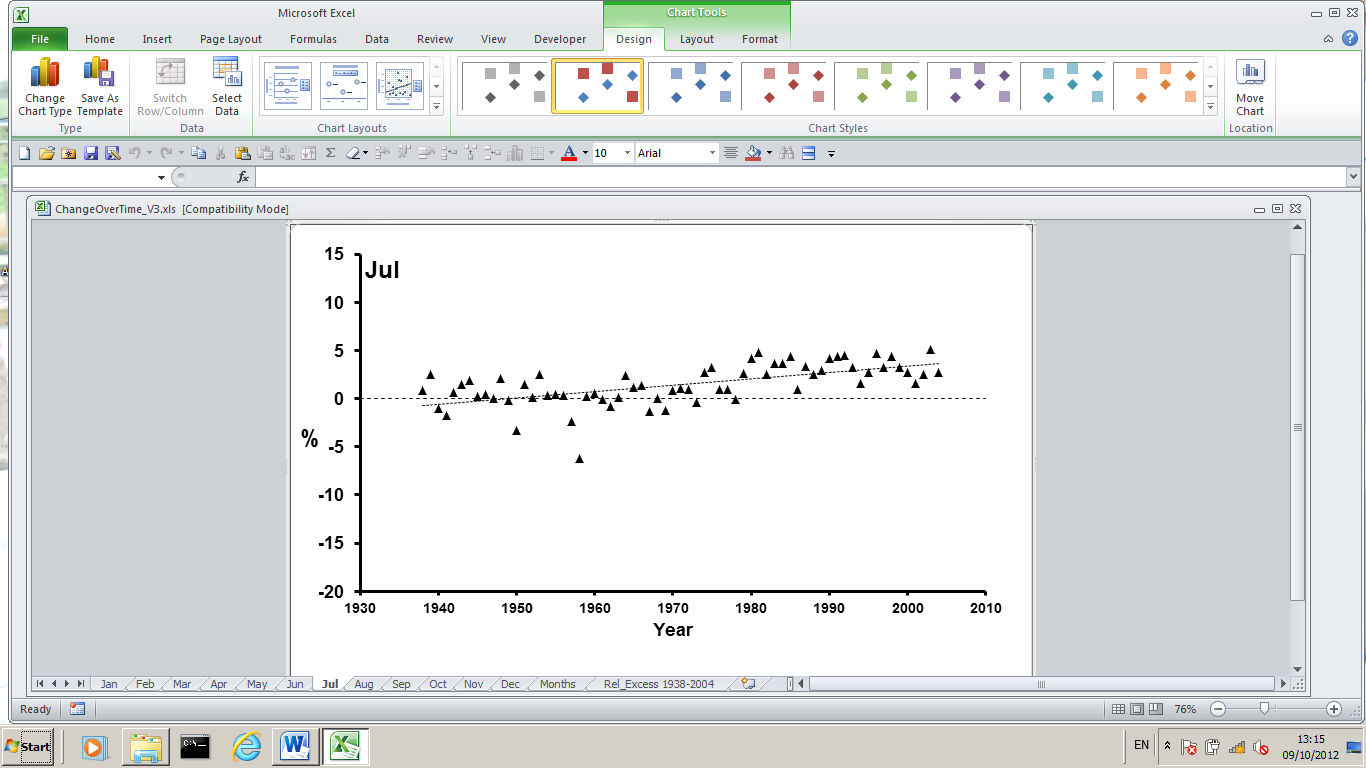


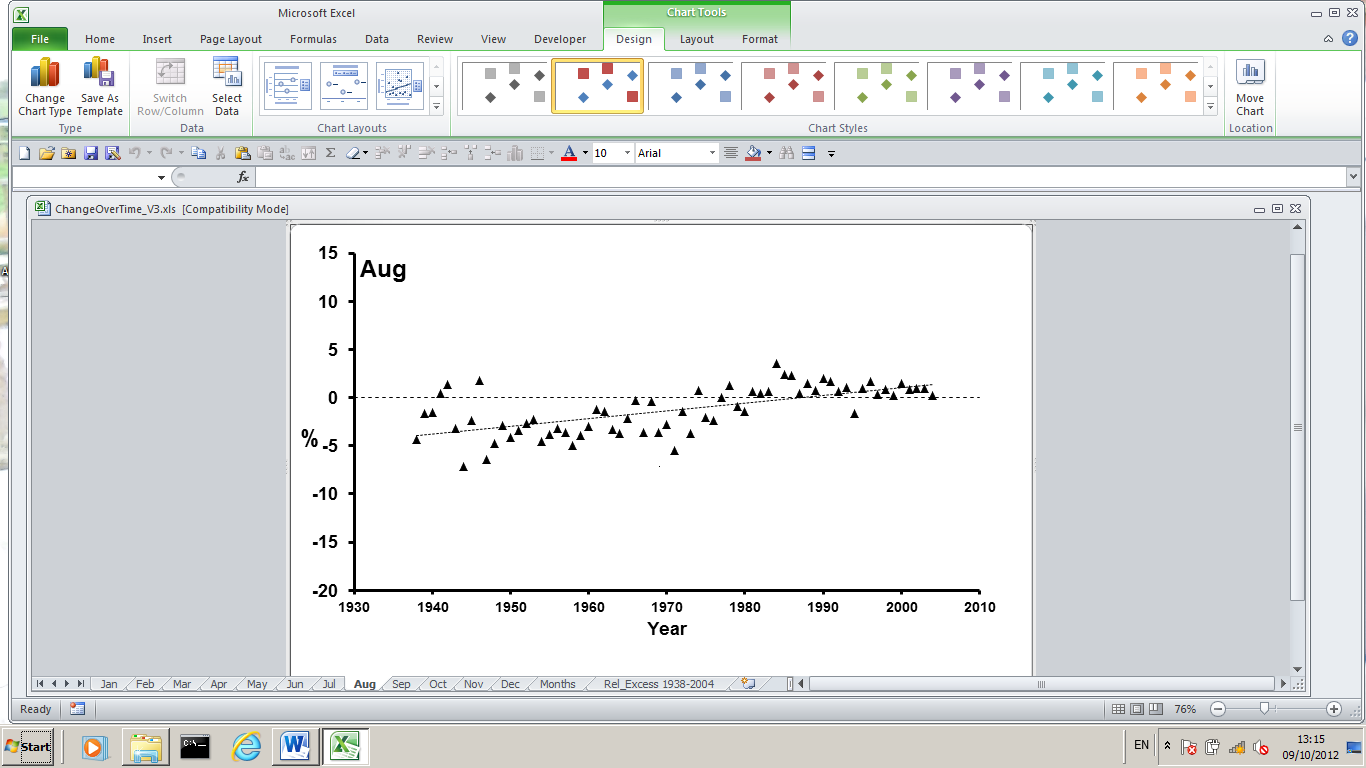


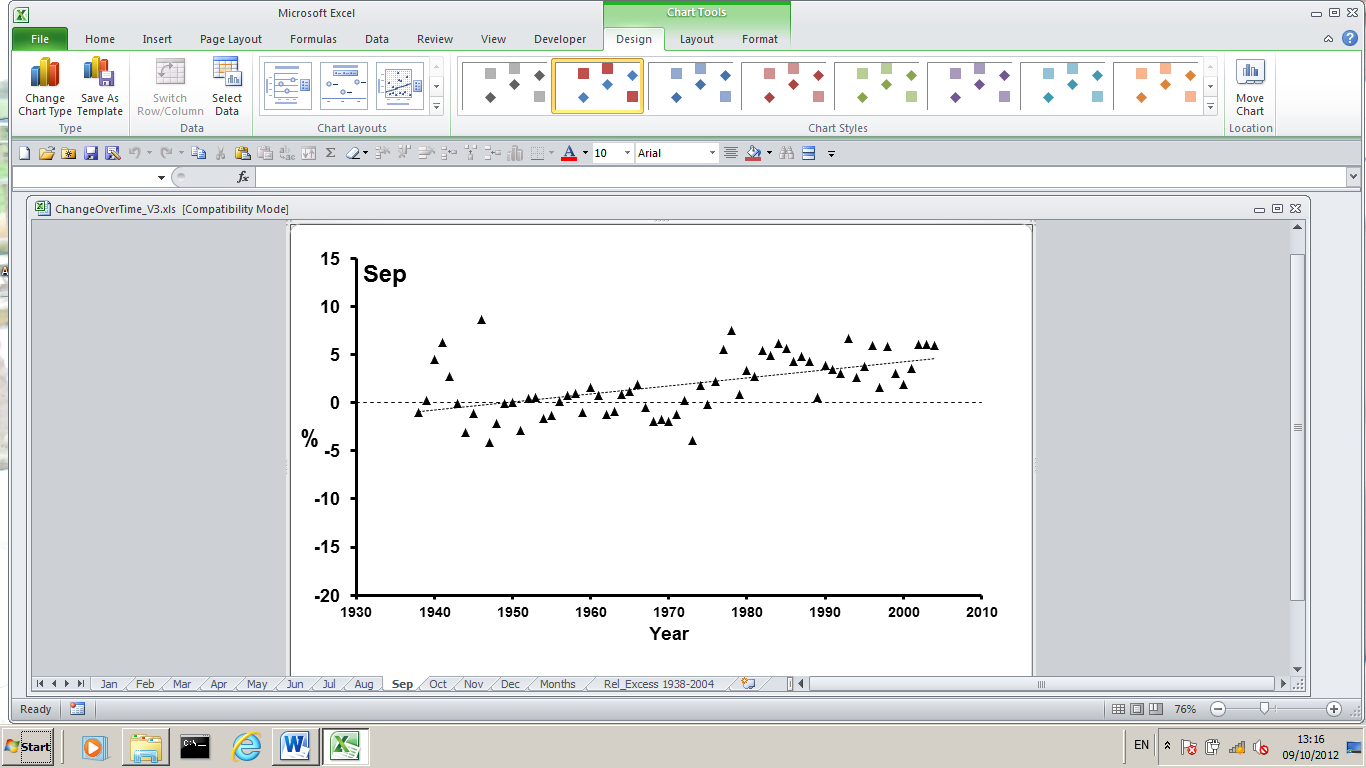


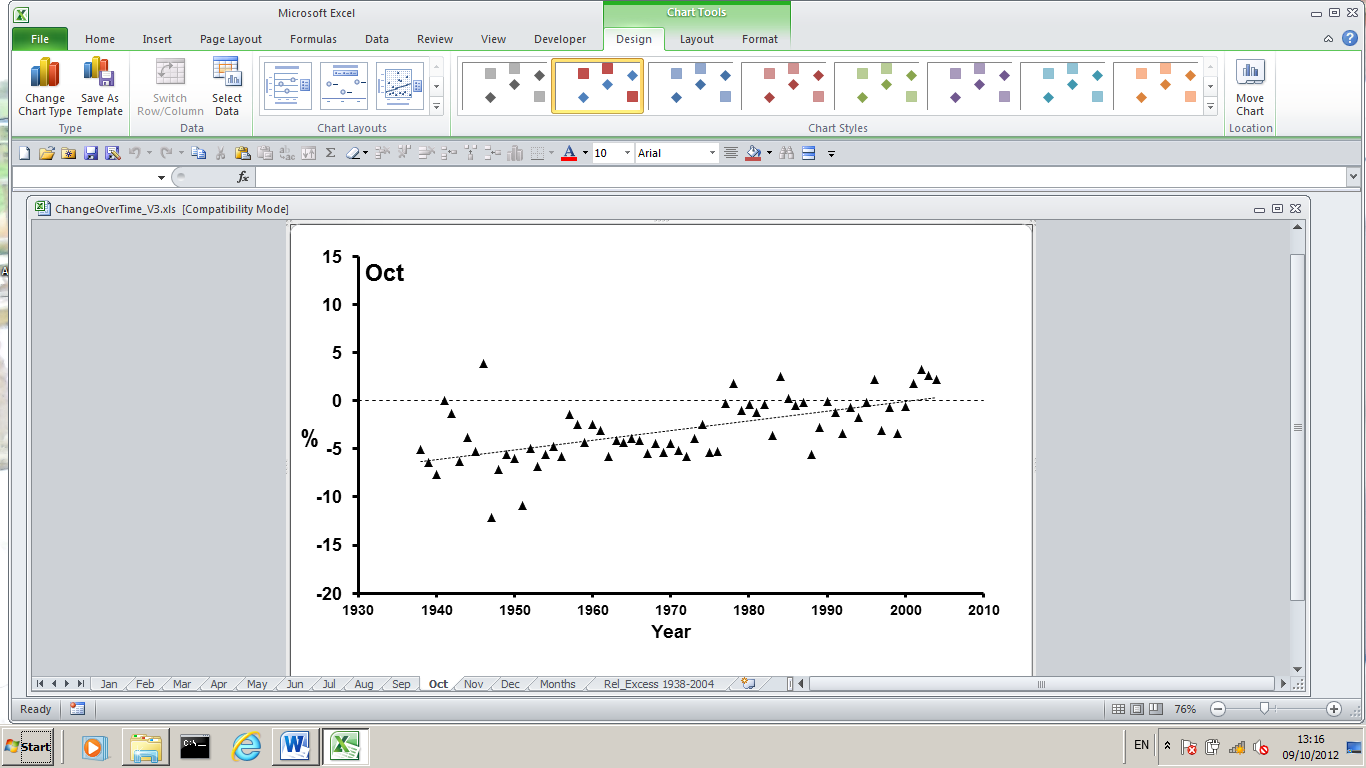


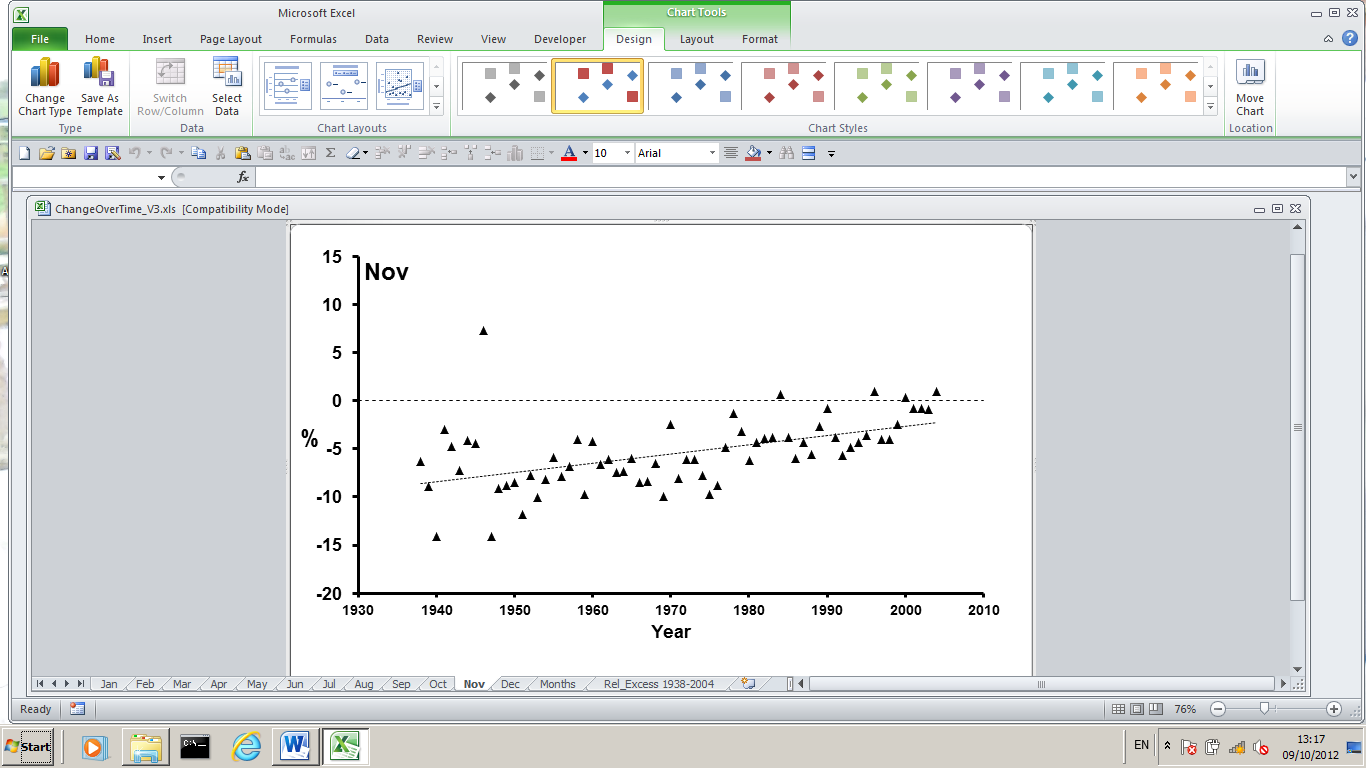


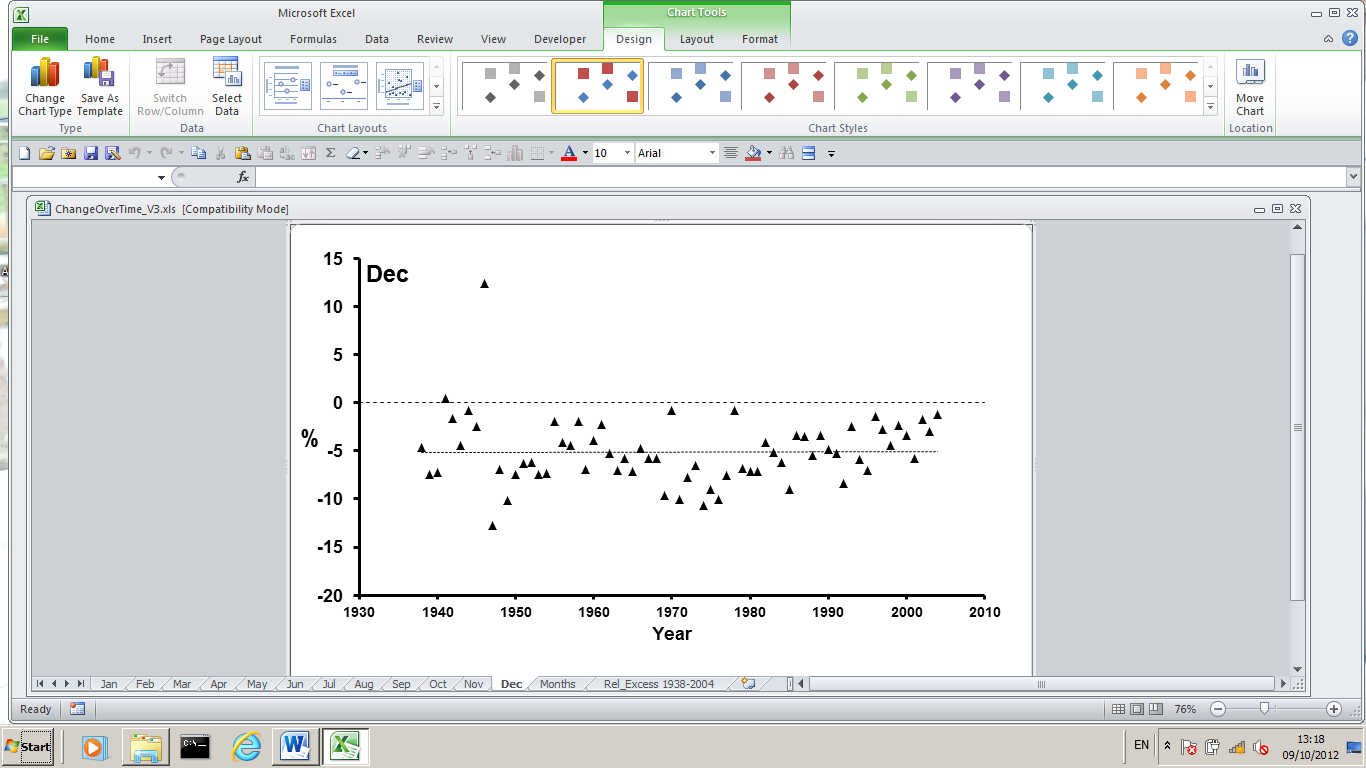


**Figure S6.** Correlation between the excess average normalised daily birth rate and year of birth (YOB) in the UK. A linear trend line is shown in each panel indicating that the tendency to excess births in the spring and reduced births in the winter has decreased over time.

# Multiple sclerosis

Given the established variation in the frequency of multiple sclerosis with respect to geography, age and gender^1, 2^ we used this disease to test the confounding effects of the structure present in population birth rate data. Several groups have considered the role of MOB in multiple sclerosis^3-18^ and most have reported an apparent excess in the spring (March, April and May) and/or a deficit in winter (November, December and January); with only a minority of (published) studies failing to find any evidence for such an association.^5, 6, 13, 14, 16^ Under the presumption of homogeneity each of these investigators compared the distribution of MOB observed in a collection of cases with that seen in publically available national statistics for the corresponding country (such as birth records or death certificates); and each thereby tacitly ignored the differences in the regional origin and age structure of the study groups.

Based on the web appendix provided with the multiple sclerosis prevalence meta-analysis performed by Koch-Henriksen and Sørensen^19^ we calculated a weighted average for the prevalence and latitude in 24 European countries as listed in table S4. As expected there is a significant (p=0.006) positive correlated between latitude and prevalence; see figure S7.

**Table S4.** The latitude and prevalence of 24 European countries

| **Country** | **Latitude / °North** | **Prevalence / 100000** |
| --- | --- | --- |
| Cyprus | 35.0 | 44 |
| Malta | 35.5 | 13 |
| Sicily | 37.4 | 94.4 |
| Spain | 38.8 | 55.4 |
| Portugal | 39.1 | 46 |
| Greece | 39.7 | 74.7 |
| Sardinia | 40.6 | 132 |
| Bulgaria | 42.6 | 39 |
| Italy | 44.2 | 77.5 |
| Switzerland | 47.0 | 110 |
| Austria | 47.3 | 98 |
| France | 48.8 | 120 |
| Belgium | 51.0 | 88 |
| Wales | 51.3 | 128.3 |
| Germany | 51.7 | 65.8 |
| England | 51.7 | 108.3 |
| Ireland | 53.8 | 181.8 |
| Denmark | 56.0 | 123 |
| Scotland | 56.0 | 190.3 |
| Estonia | 58.8 | 51 |
| Norway | 61.4 | 141.7 |
| Finland | 61.9 | 79.2 |
| Sweden | 62.0 | 163.5 |
| Iceland | 64.5 | 100 |


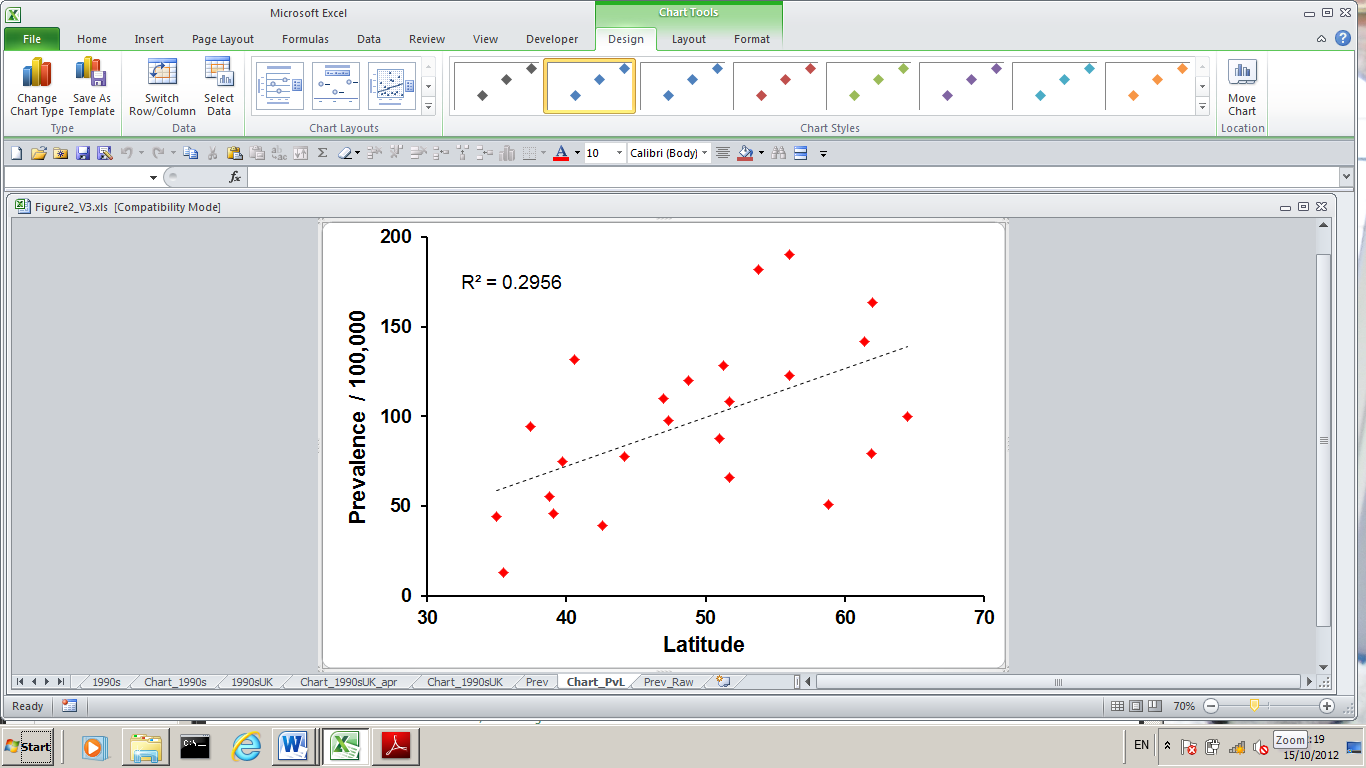


**Figure S7.** The correlation between the prevalence of multiple sclerosis and latitude in European countries; the dotted line shows the linear trend-line.

Given that prevalence and birth rate are both independently correlated with latitude it is unsurprising that these variables are also correlated with each other. Figure S8 shows the relation between the prevalence of multiple sclerosis and the probability of birth in each month in the MOB data sets from the 16 European countries considered above. Prevalence data were taken from table S4 for all countries except the Netherlands (which was not included in the Koch-Henriksen meta-analysis^19^), for this country we took the most recent estimate described in McAlpine’s Multiple Sclerosis (4^th^ Edition); ^1^ i.e. 76 per 100000. In these data there was significant evidence (p<0.05) for positive correlation in April and June, and for negative correlation in September, October and November. Ireland seemed to be an outlier from the main distribution with an unexpectedly high prevalence and the lowest annual birth rate in the countries considered. If Ireland is excluded then there is increased evidence for correlation in the months described above and statistically significant evidence also emerges for positive correlation in February and March, and for negative correlation in December. Evidence for positive correlation in May is borderline with a p-value of 0.07. Only January, July and August don’t show any statistically significant evidence for correlation.

Using the case-control ratios established by Kurtzke^20^ as estimates of state specific prevalence reveals remarkably similar correlations in North America (see Figure S9). These correlations are significant in every month, positive in March, April, May, June and July, and negative in August, September, October, November, December, January and February. These correlations were substantial (R^2^ > 50%) and highly significant (p < 3x10^-9^) for most months (except February, July and August).

The prevalence figures used in the analysis of the lactase SNP analysis presented in the BOX in the main text are the most up to date and reliable we could find.^21-27^


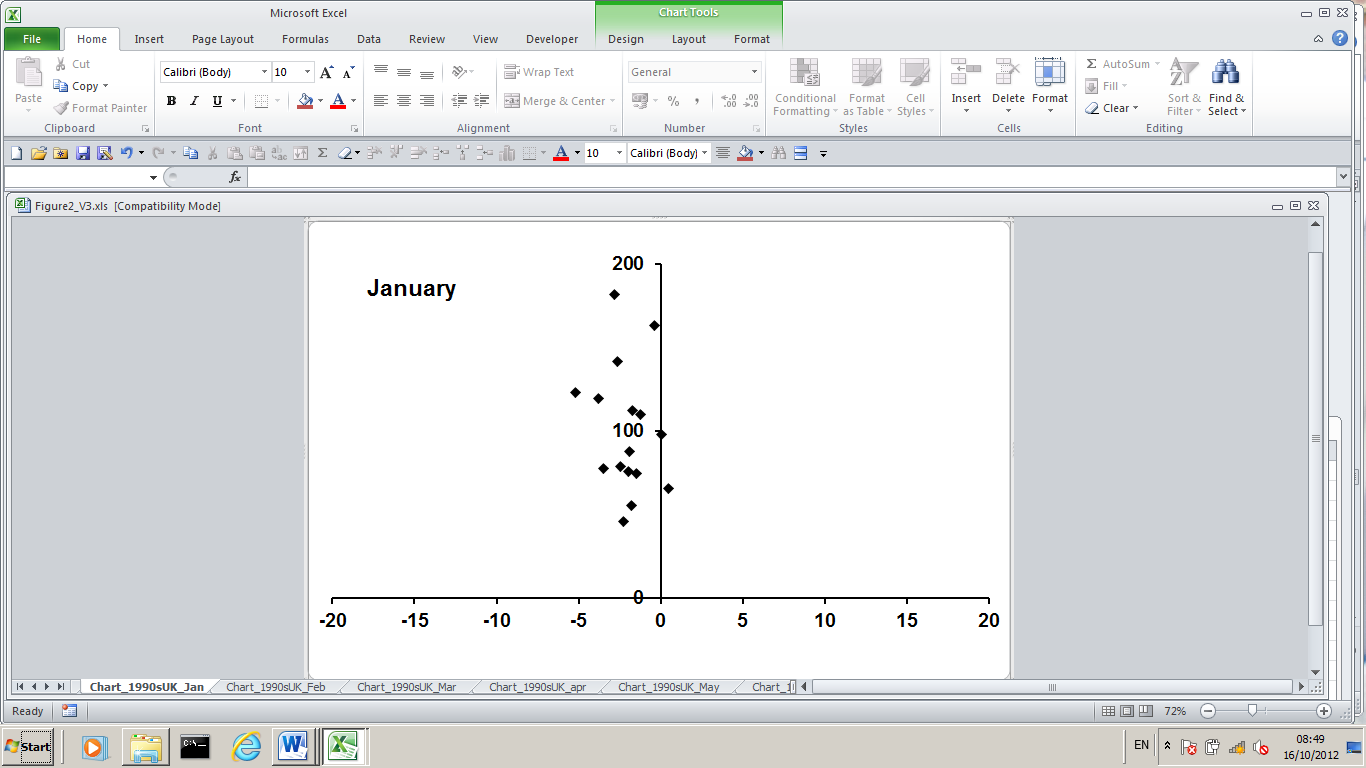


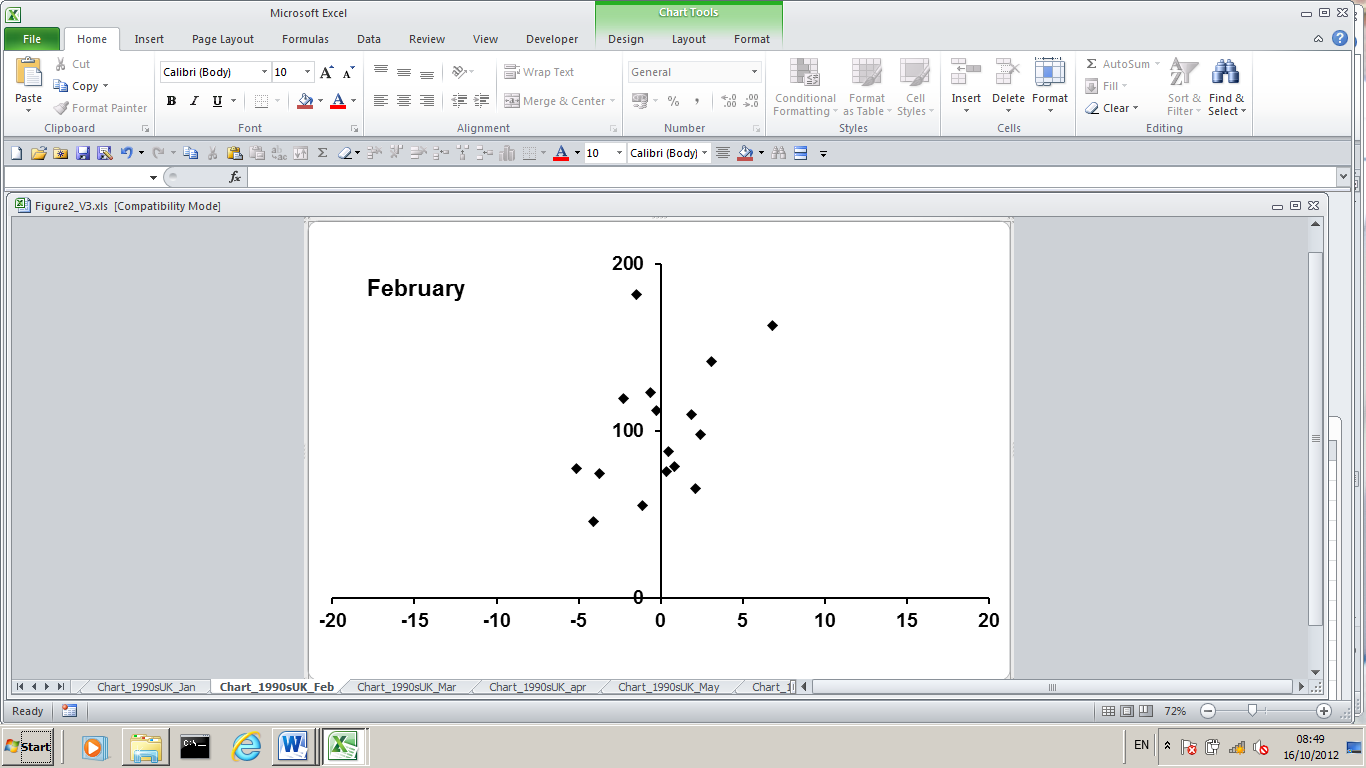


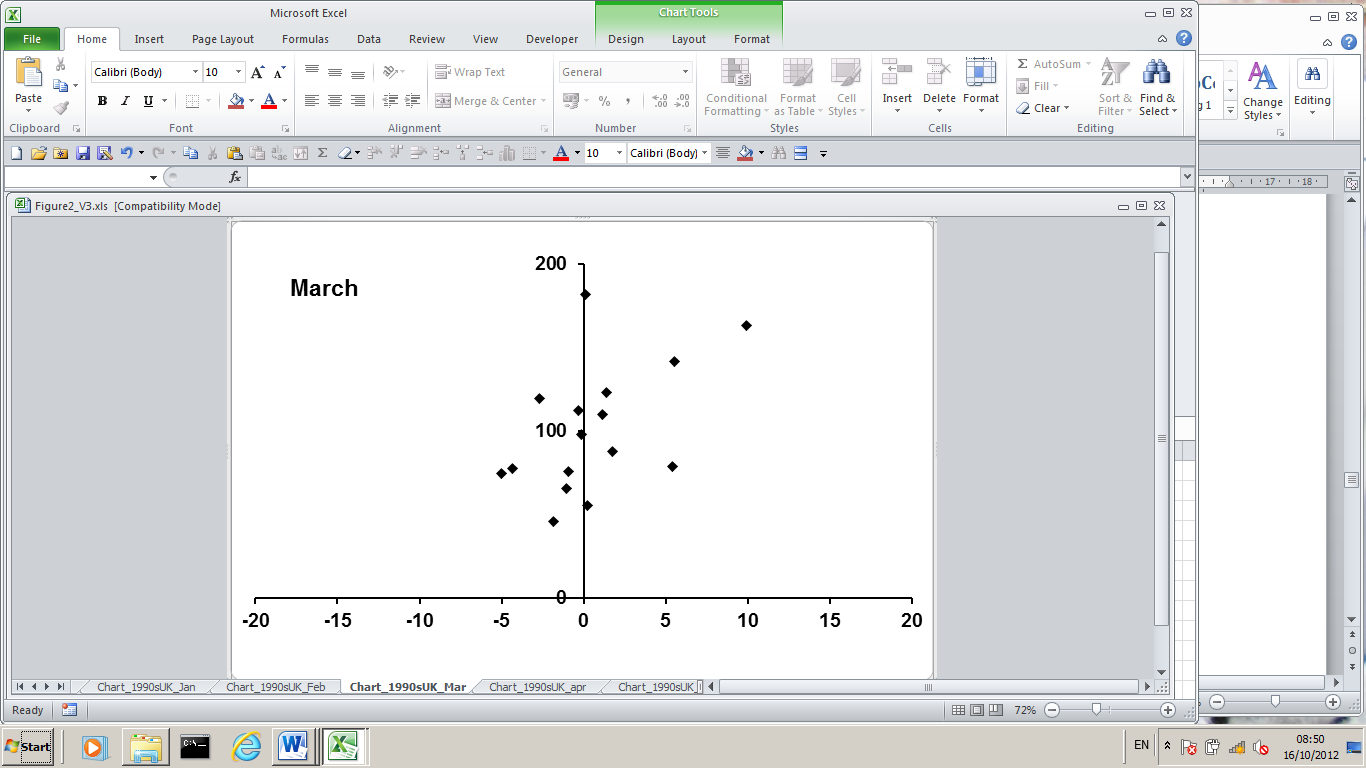


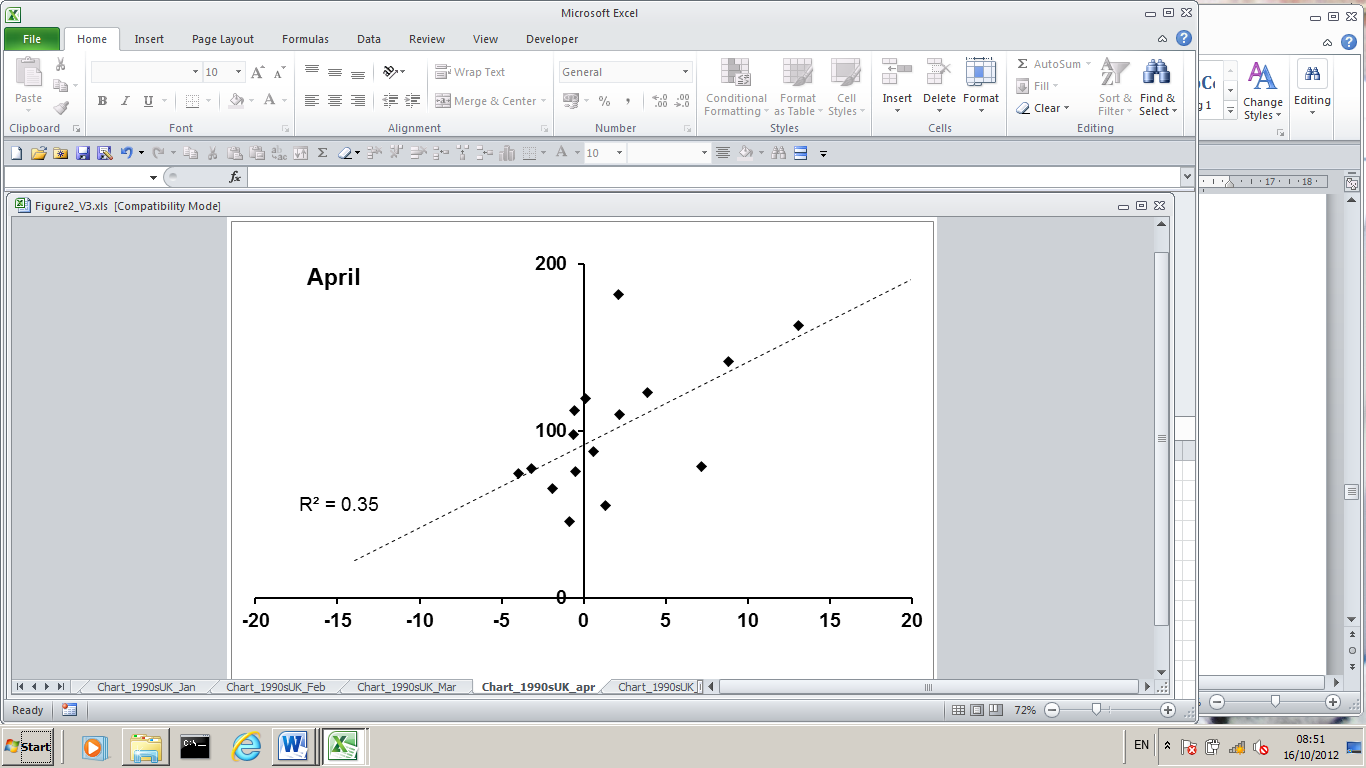


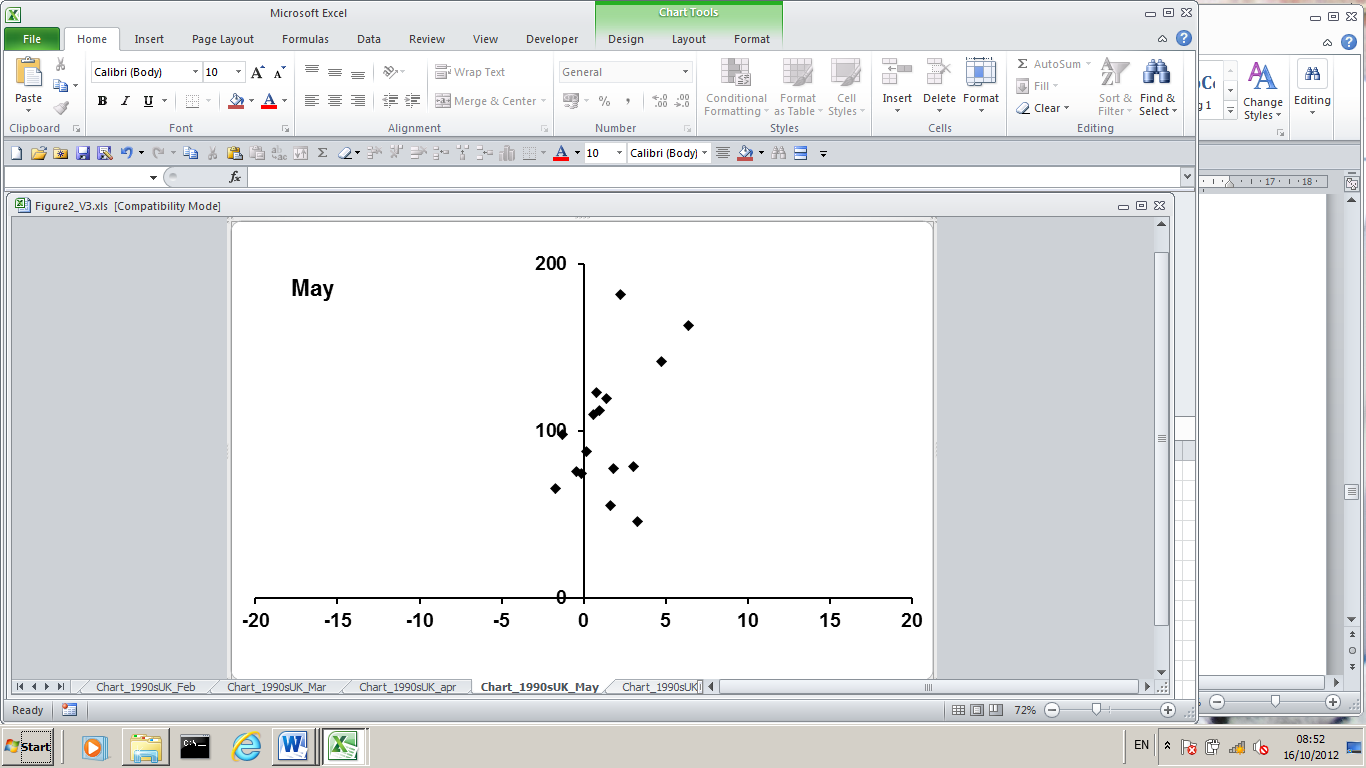


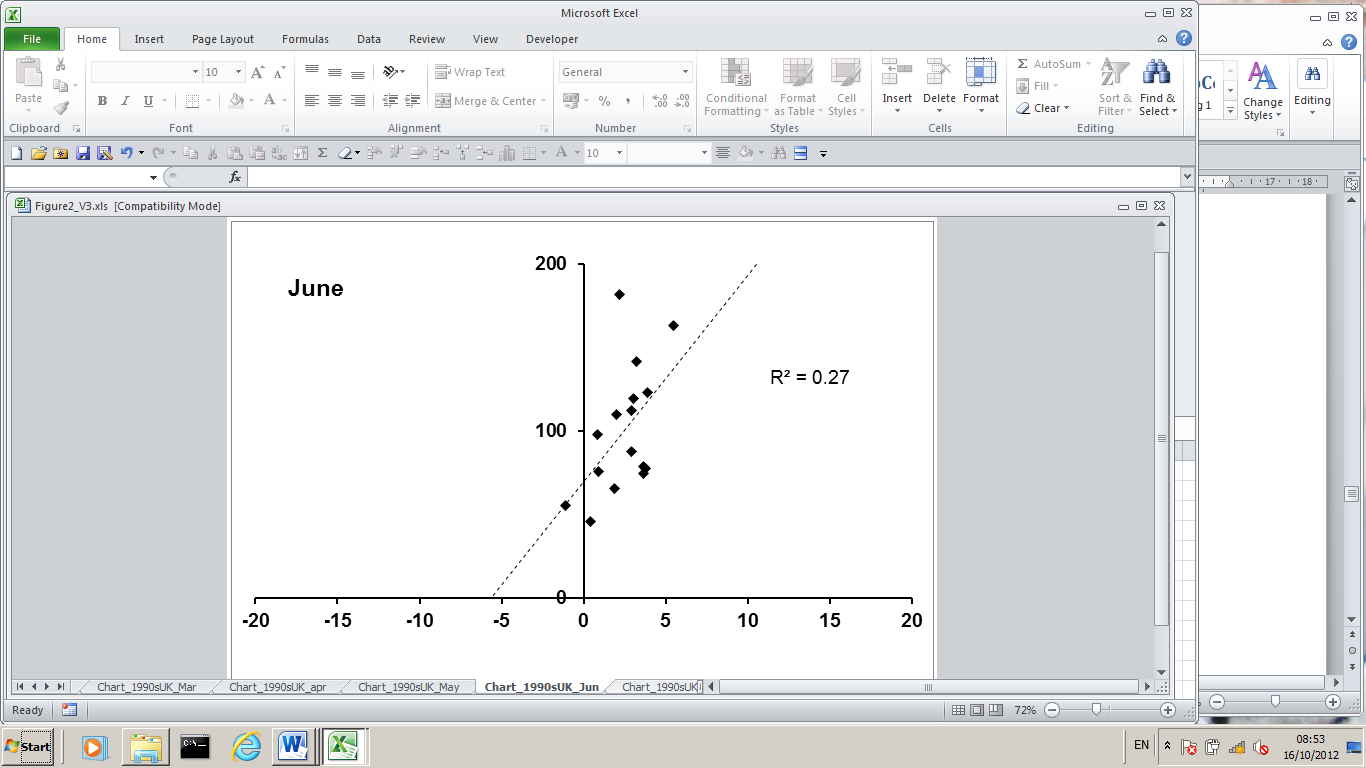


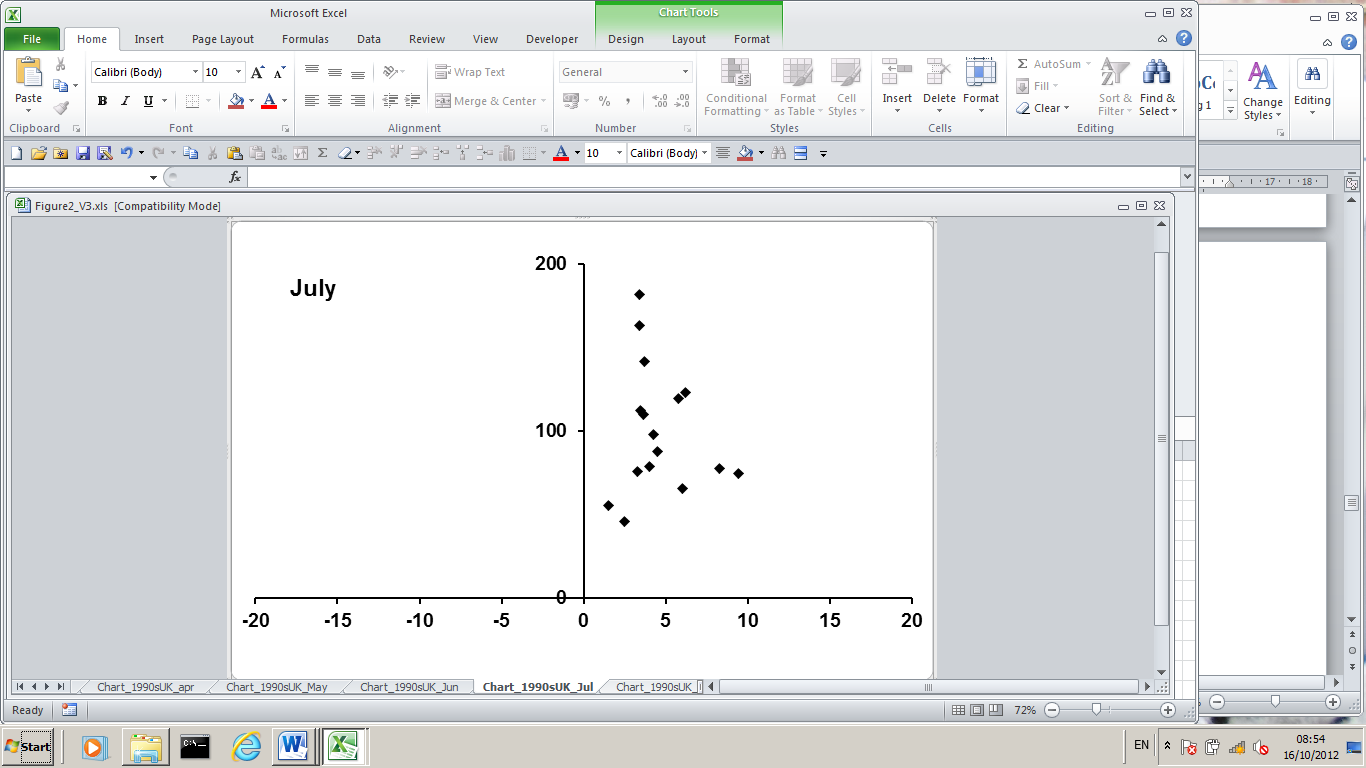


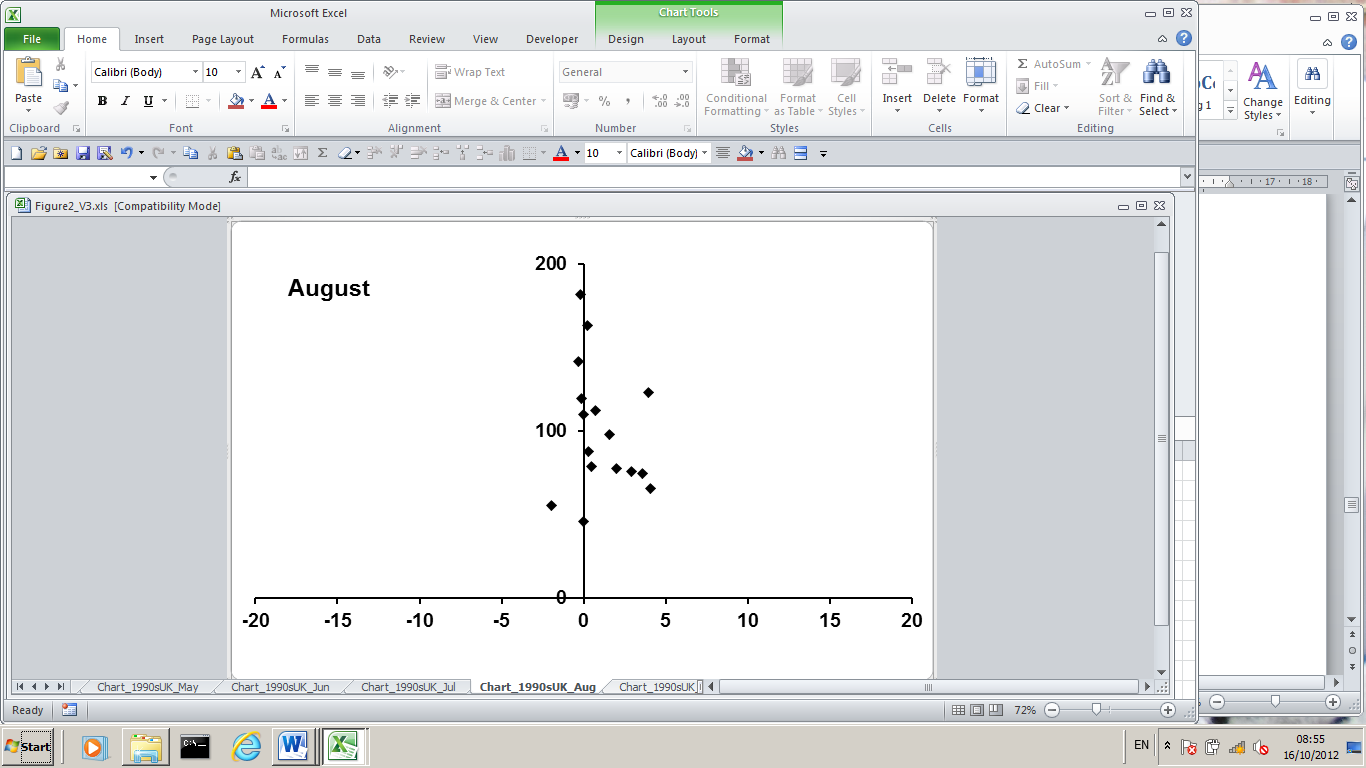


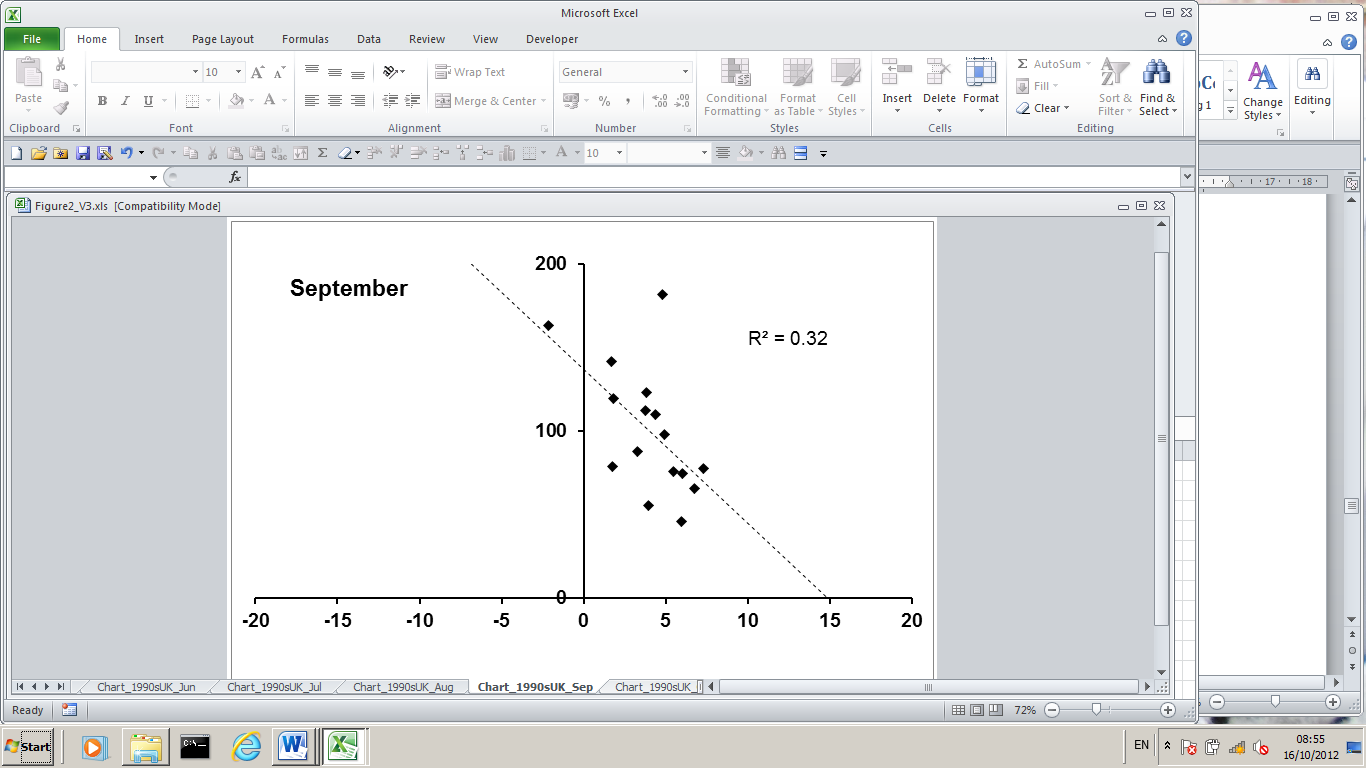


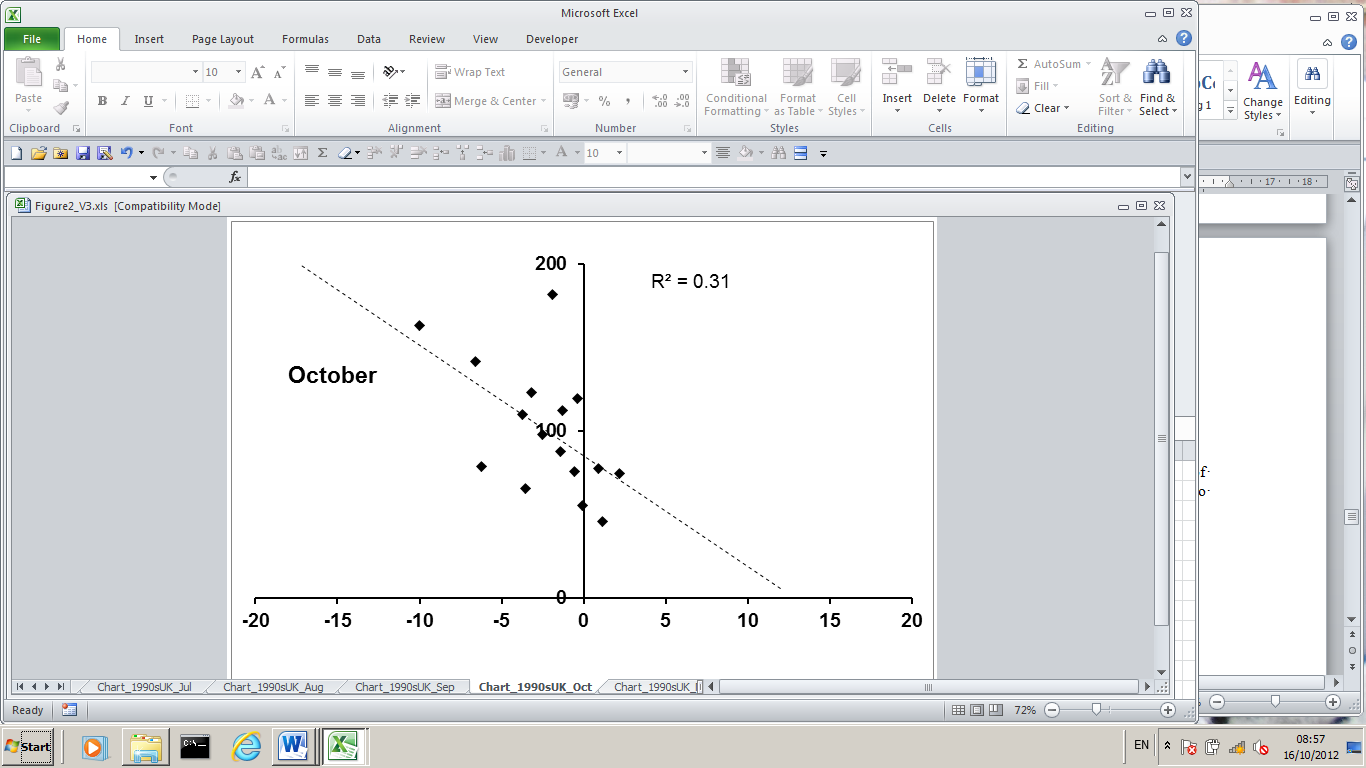


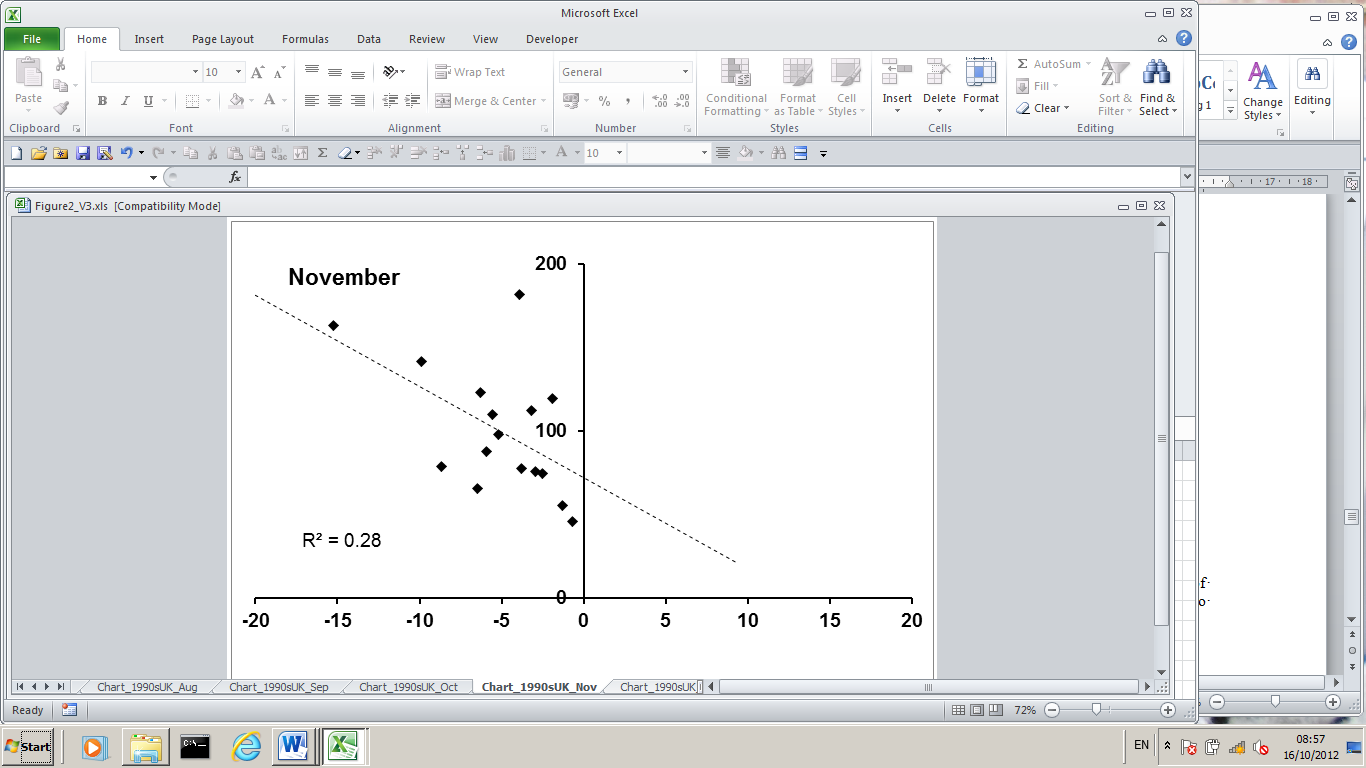


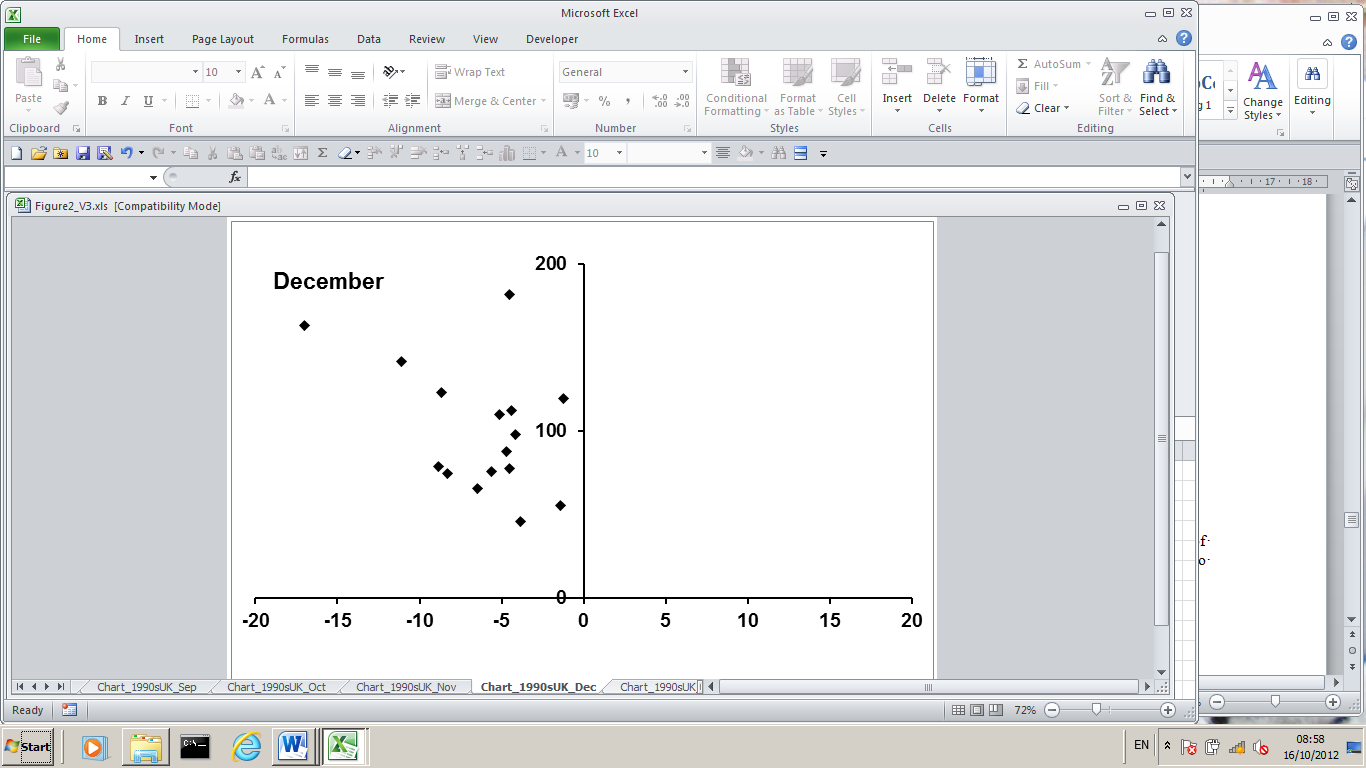


**Figure S8.** Correlation between the excess average normalised daily birth rate (%) in each month and the prevalence of multiple sclerosis in 16 European countries (one panel for each month as indicated). A trend line is shown in each panel for which there is statistically significant evidence of correlation (p<0.05). Birth rates are based on the average of population specific MOB data sets for the 10 year period 1991-2000.


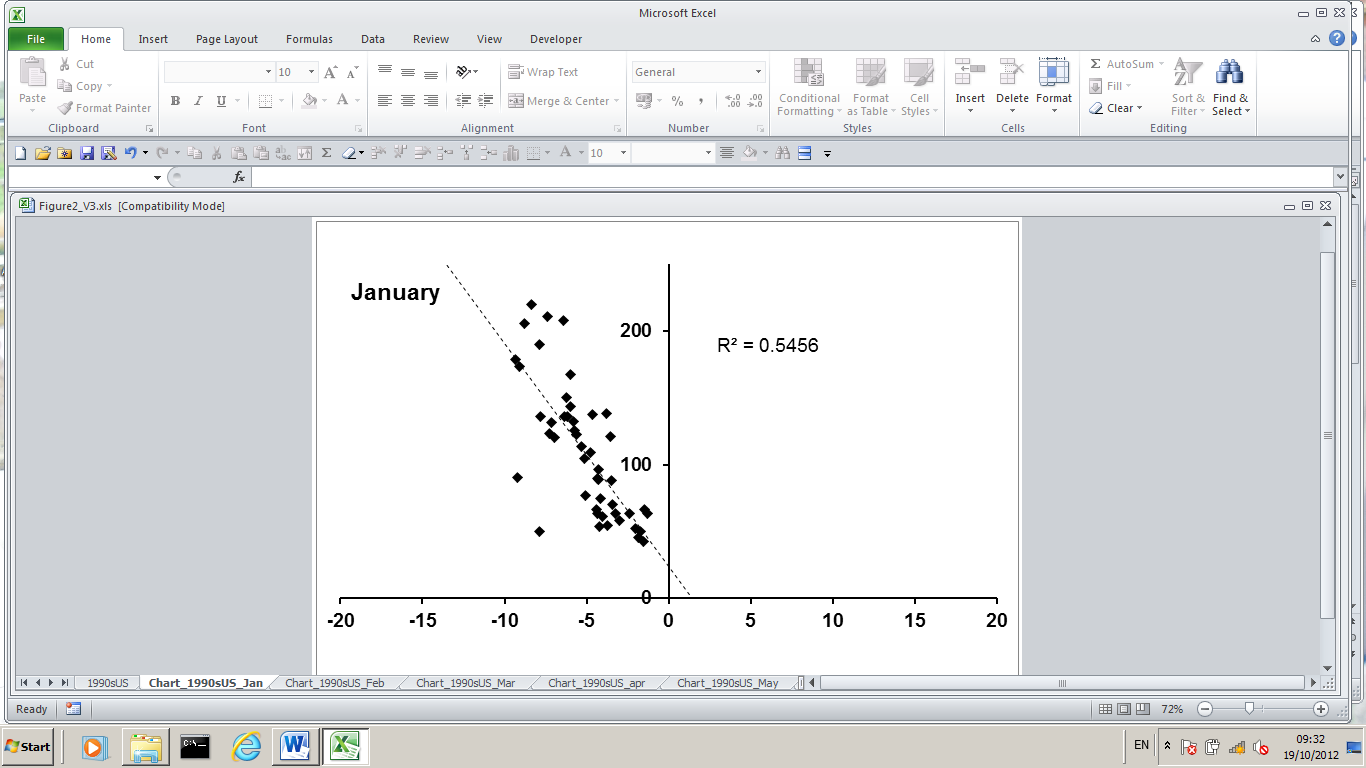


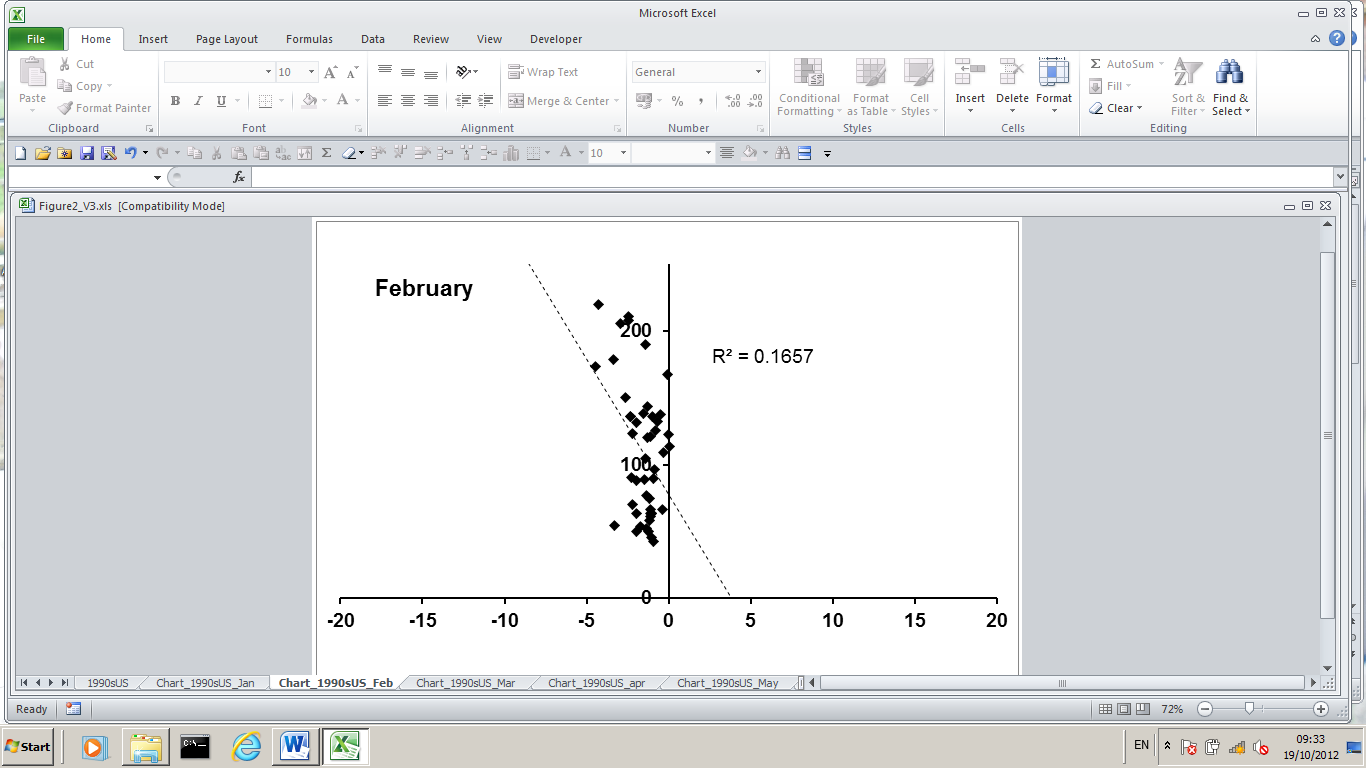


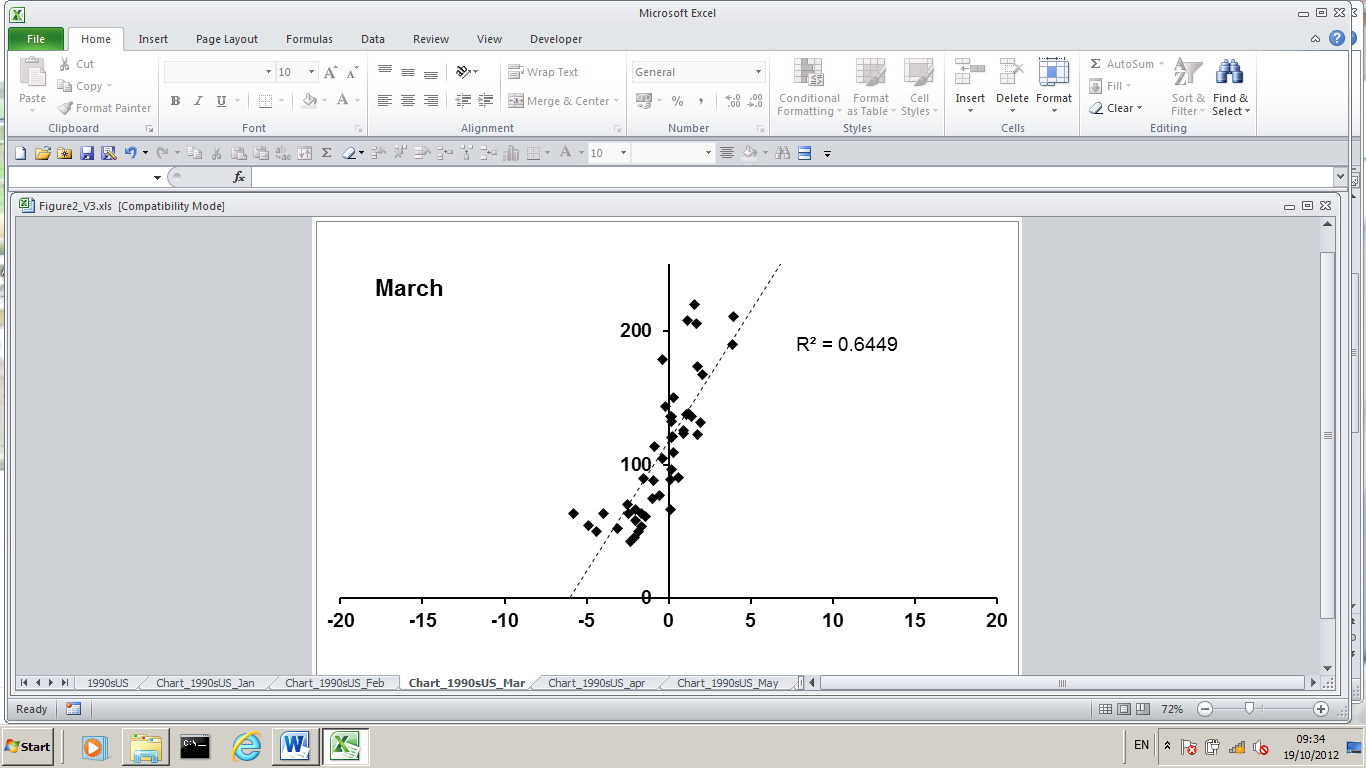


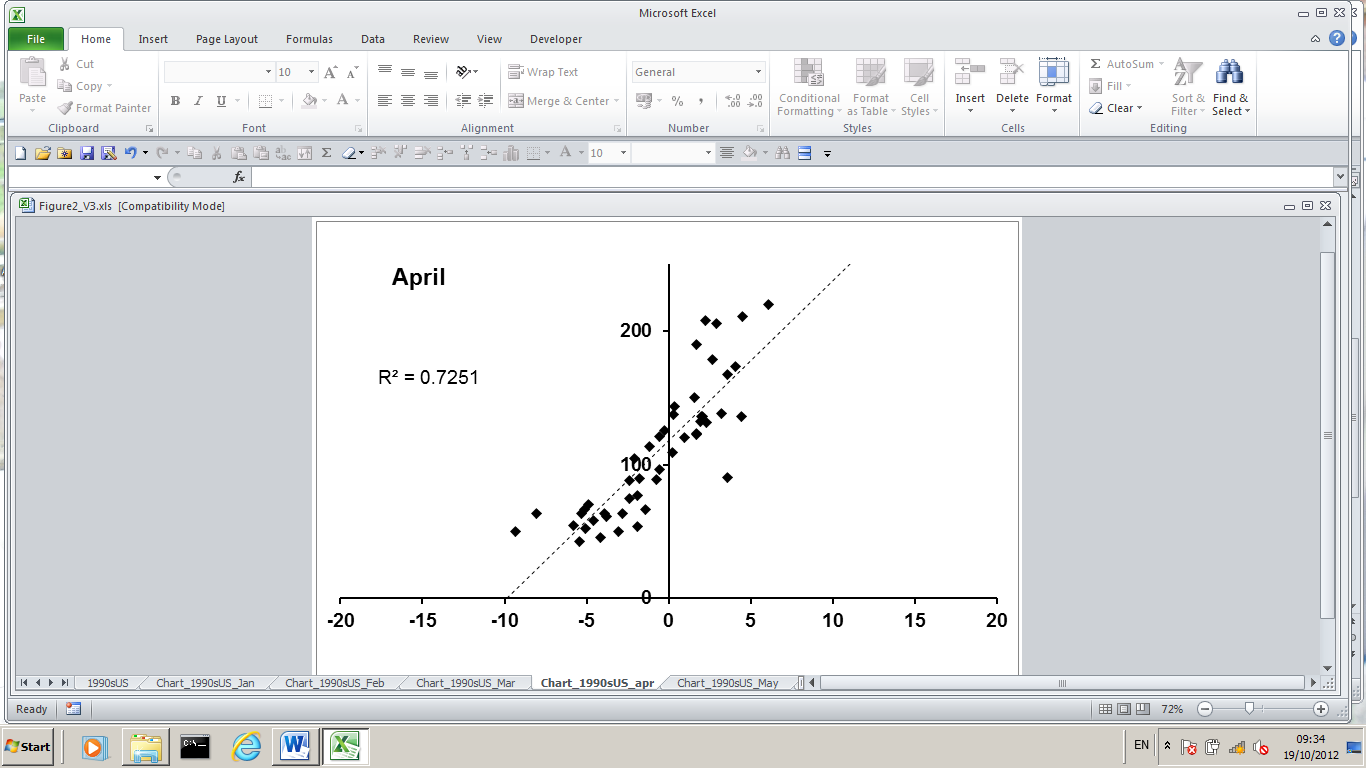


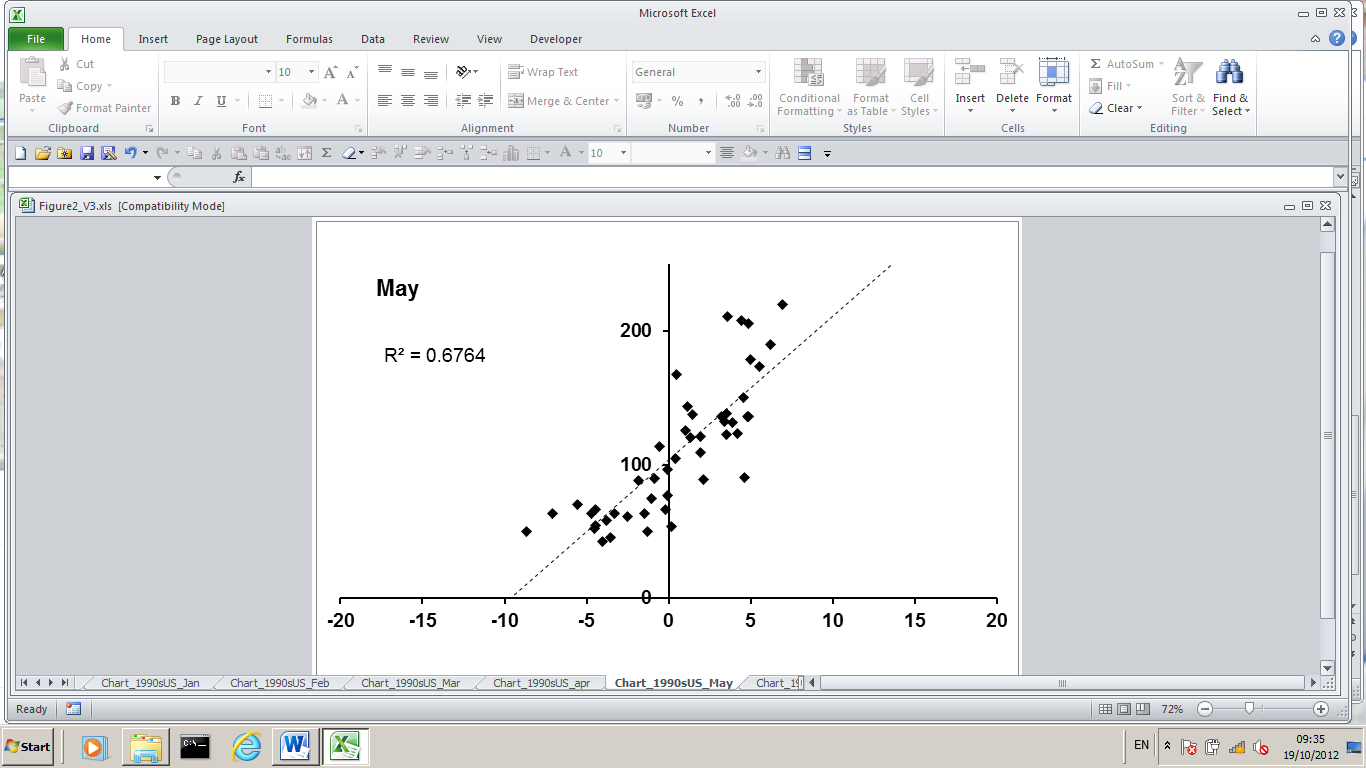


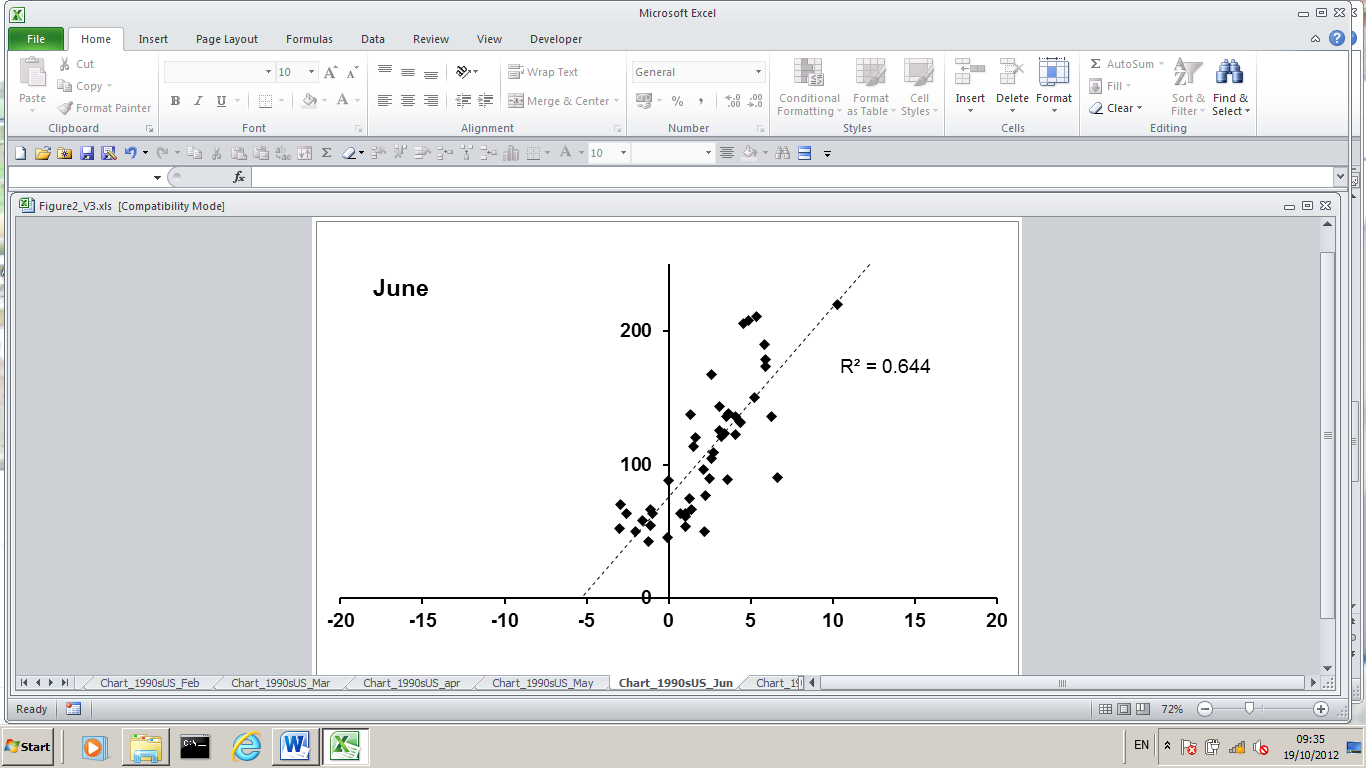


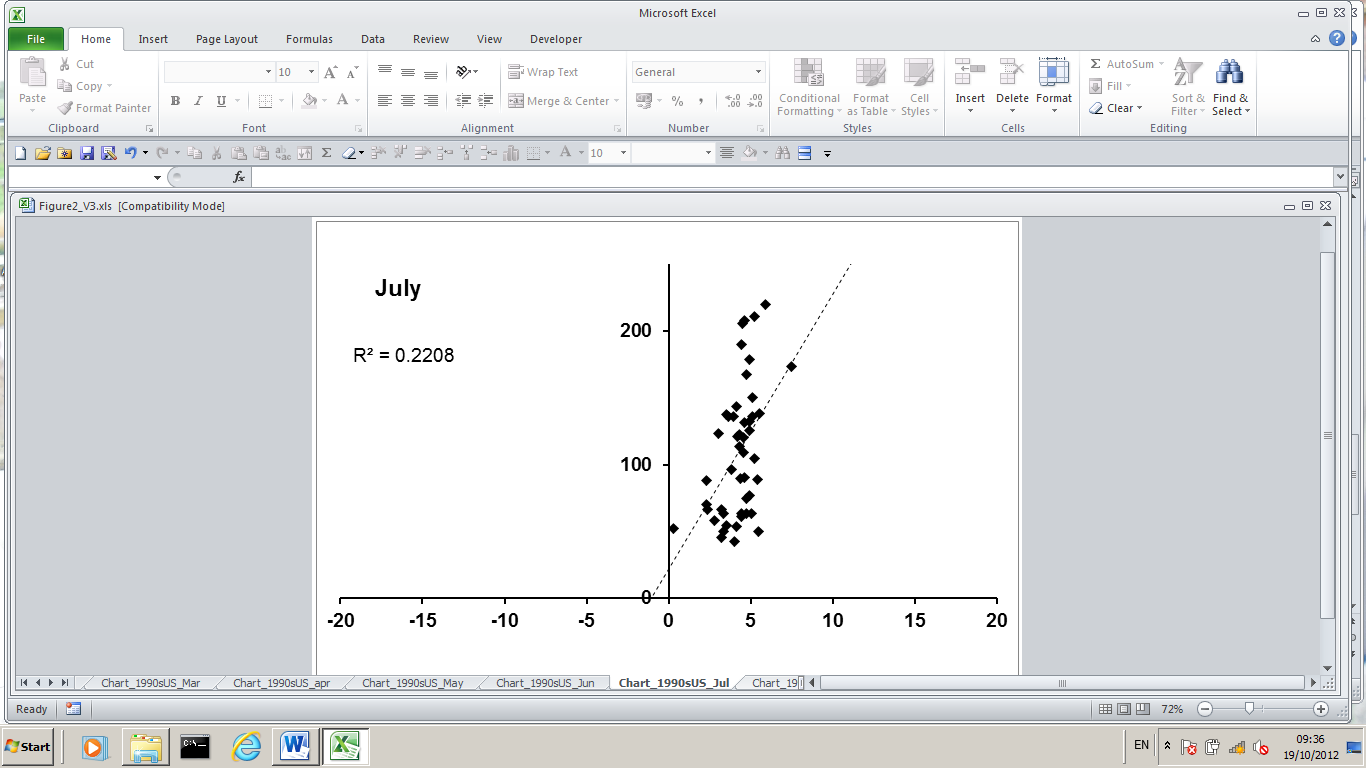


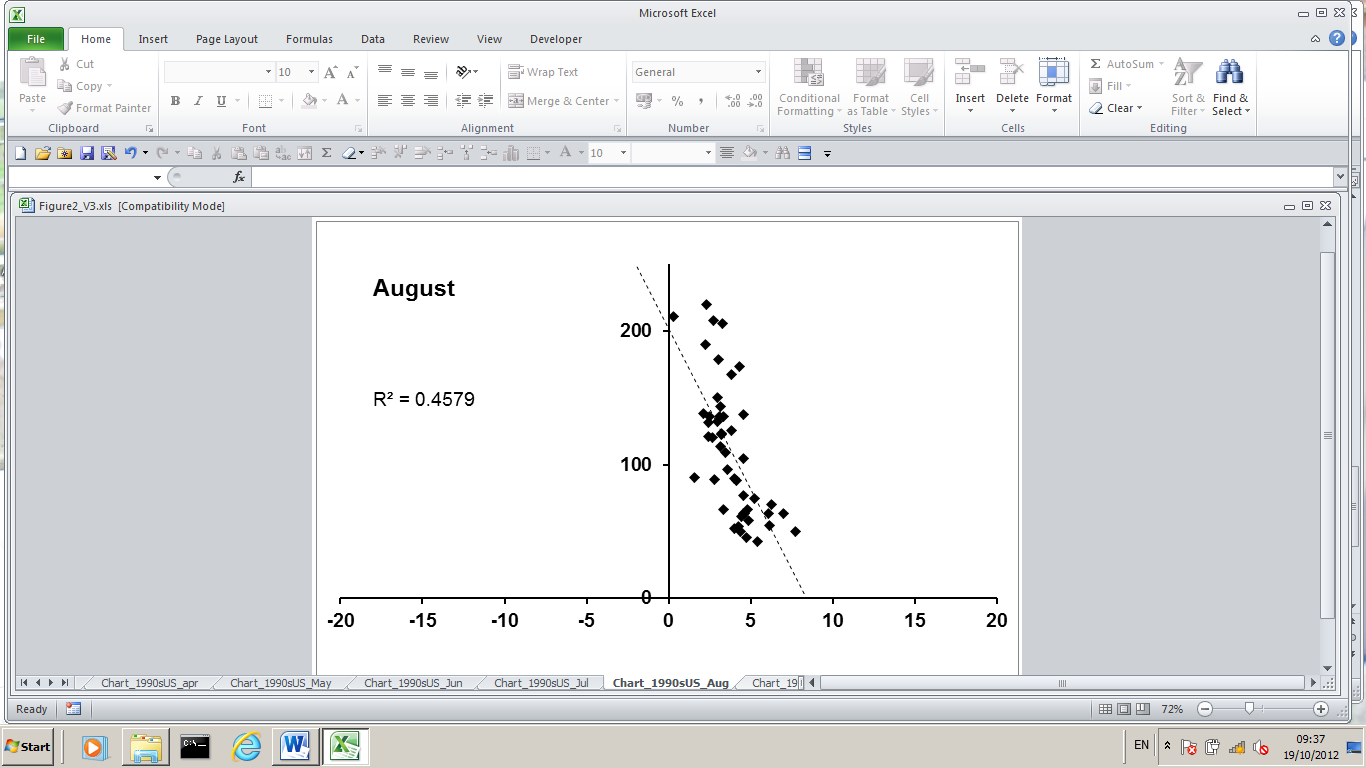


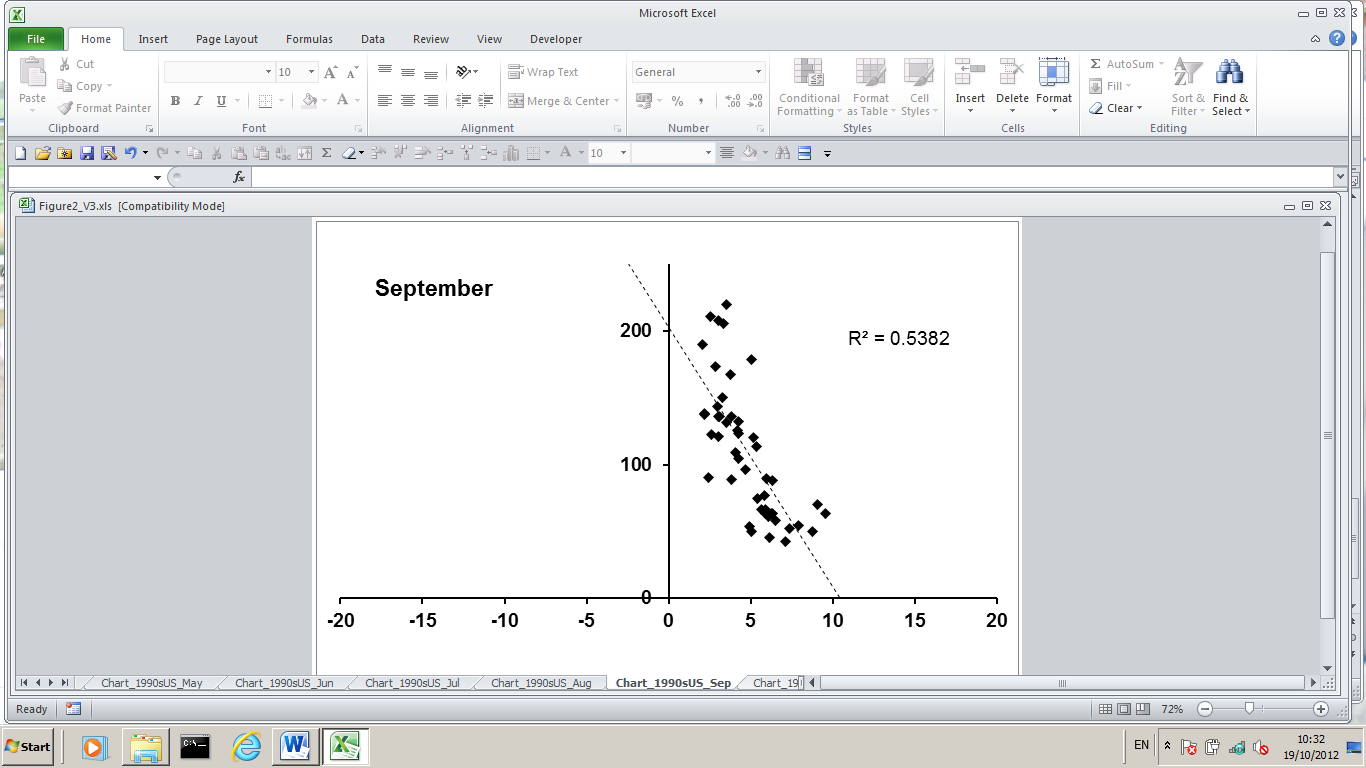


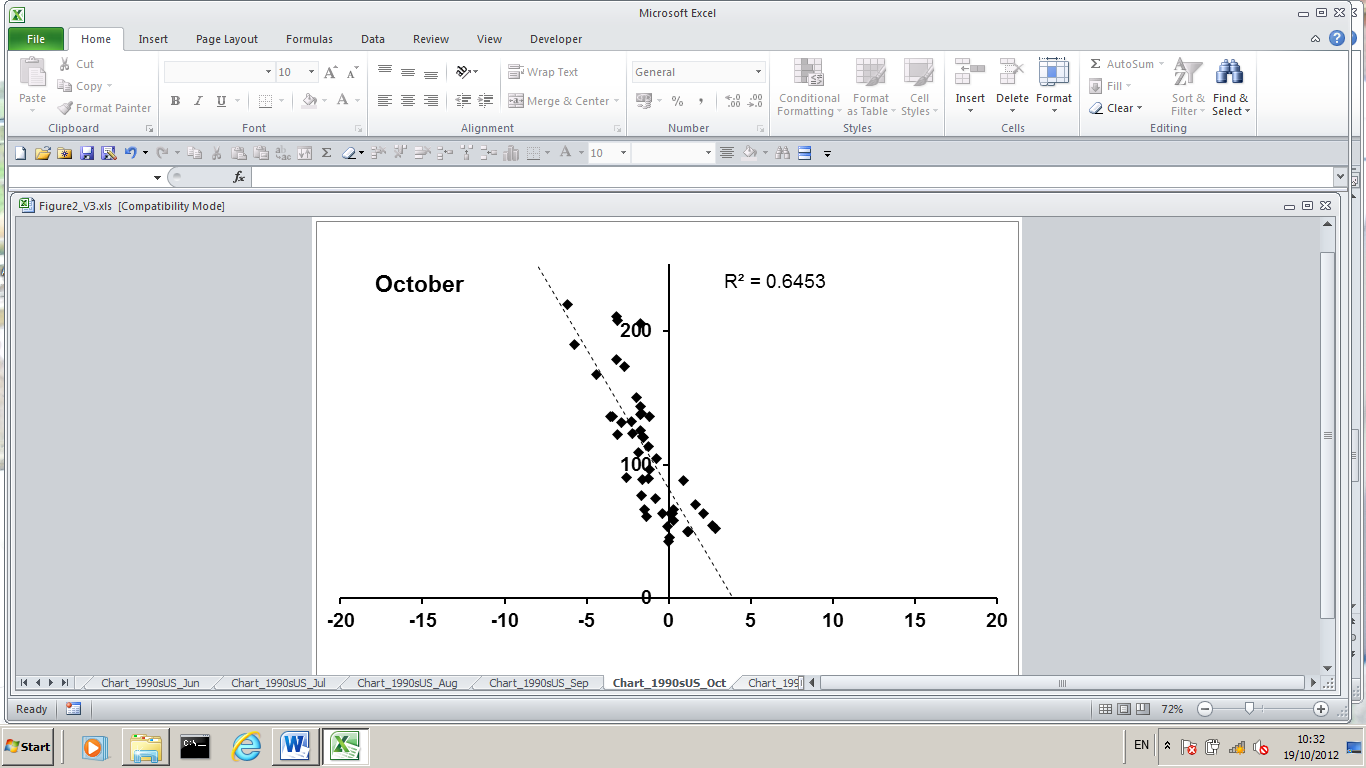


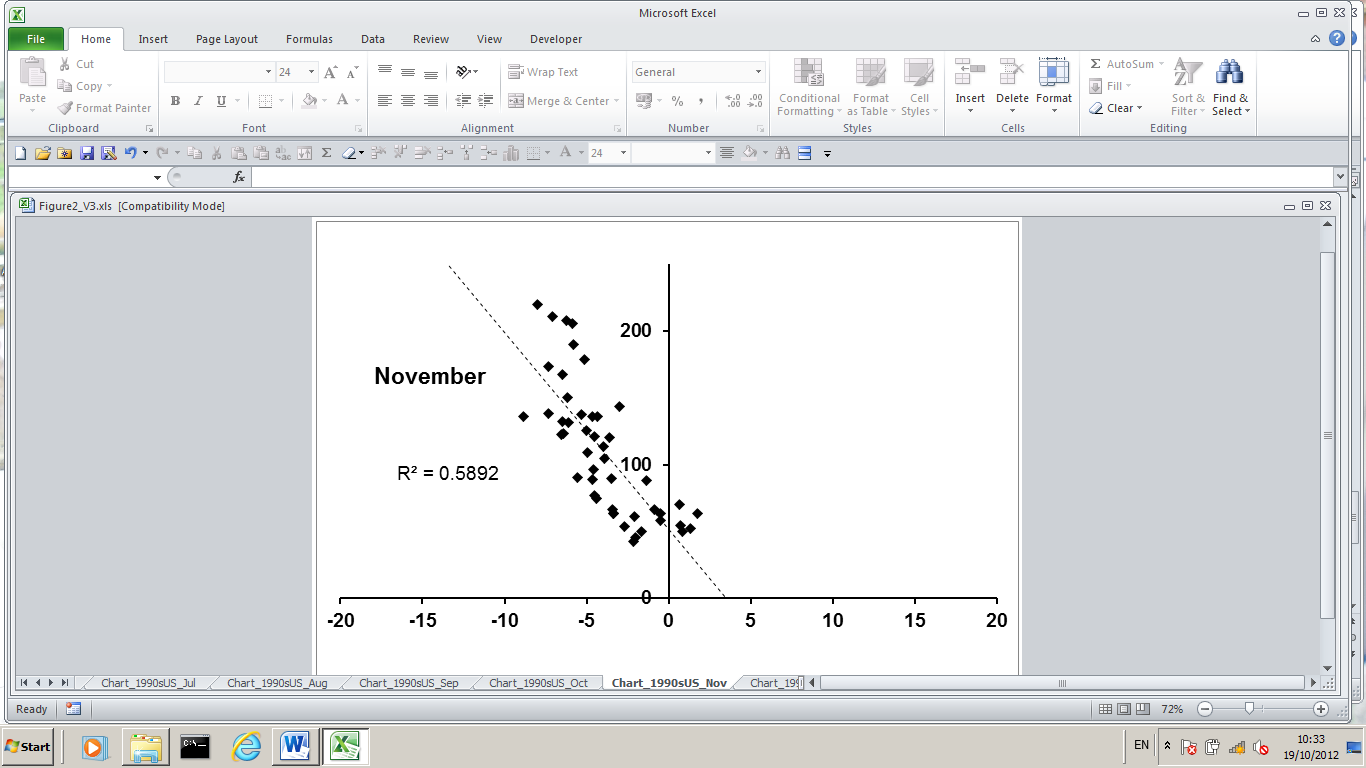


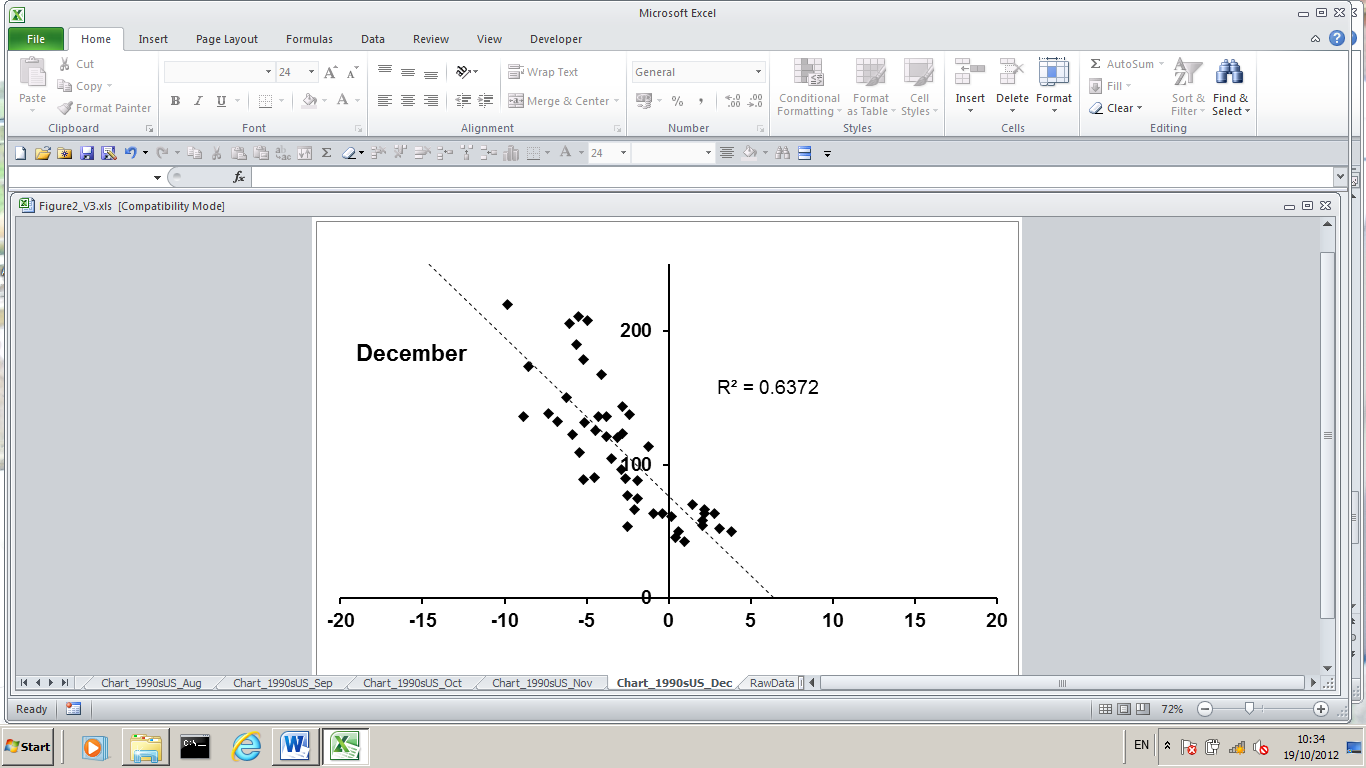


**Figure S9.** Correlation between the excess average normalised daily birth rate (%) in each month and the Kurtzke^20^ multiple sclerosis case-control ratio in 48 American states (one panel for each month as indicated). There was statistically significant evidence of correlation (p <0.004) in every month so a trend line is included on each panel. Birth rates are based on the average of population specific MOB data sets for the 10 year period 1991-2000.

# Multinomial modelling

We used a multinomial logit approach^28^ to model how the probability of an individual being born in each month depends on different covariates.

If p_ij_ represents the probability of individual *i* being born in month *j* (*j*=1 represents January, *j*=12 represents December), then a multinomial logit model fits 11 separate models:

$$\begin{matrix} \log\left(\frac{p_{i2}}{p_{i1}}\right)=\beta_{2}X_{i} \\ \log\left(\frac{p_{i3}}{p_{i1}}\right)=\beta_{3}X_{i} \\ \vdots\\ \log\left(\frac{p_{i12}}{p_{i1}}\right)=\beta_{12}X_{i}, \\ \end{matrix}$$

where X_i_ is the vector of covariates for individual *i*.

The individual probabilities are then:

$$\begin{matrix} p_{i1}=\frac{1}{1+\sum_{k=2}^{12} \exp(\beta_{k}X_{i})} \\ p_{i2}=\frac{\exp(\beta_{2}X_{i})}{1+\sum_{k=2}^{12} \exp(\beta_{k}X_{i})} \\ \vdots\\ p_{i12}=\frac{\exp(\beta_{12}X_{i})}{1+\sum_{k=2}^{12} \exp(\beta_{k}X_{i})}. \end{matrix}$$

To fit this model we used the function multinom in the R package nnet.^29^

# UK Government Office Region level MOB data sets

For administrative and statistical purposes England is divided into 9 “Government Office Regions” (North East, North West, Yorkshire and the Humber, East Midlands, West Midlands, East of England, South East, South West and London), and together with Scotland and Wales these equate to the 11 GOR described in the BOX in the main text. This system was established in the late 1990s prior to which there were only 10 regions as London was considered as part of the South East. The boundaries of these 10 original regions were otherwise broadly similar. So that data would be consistent across the 44 available years (1965-2008 inclusive) we included the data for London within the South East in each year and considered just the 10 regions. In total we therefore had 440 MOB data sets summarising nearly 33 million births.

The proportion of these MOB data sets showing an excess or a deficit in the average normalised daily birth rate in each month is shown in figure S10. For each month the mean value of the average normalised daily birth rate and its standard deviation (over the 44 year period) are shown in table S5.


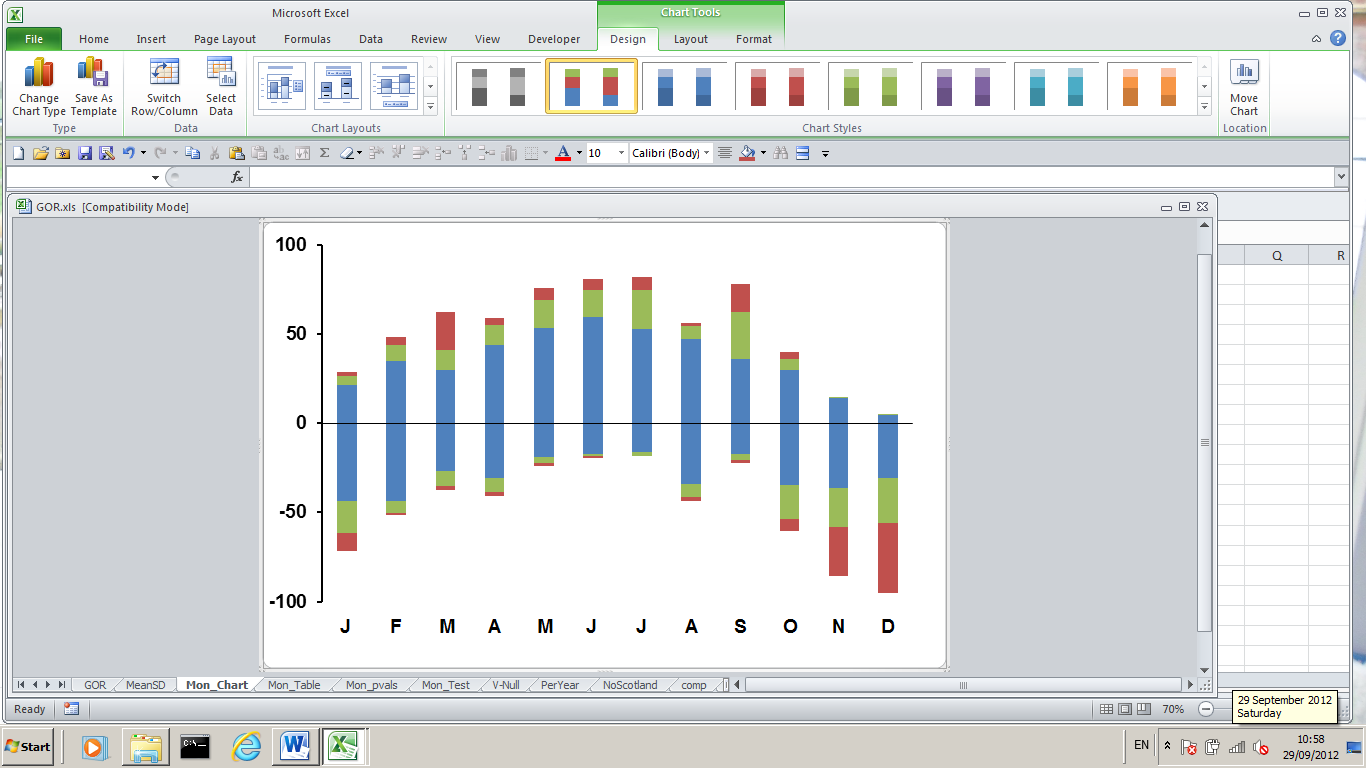


**Figure S10.** The proportion (%) of UK GOR MOB data sets (n=440) showing excess (above) or deficit (below) in the average normalised daily birth rate in each month. The proportion (%) of data sets showing significant difference with p<0.0045 (=0.05/11) is shown in red while the proportion with p<0.05 is shown in green, the proportion that is not statistically significantly different is shown in blue.

**Table S5.**The mean and standard deviation of the average normalised daily birth rate in the 10 UK GOR

| **Region** | **N** | **January** | **February** | **March** | **April** | **May** | **June** |
| --- | --- | --- | --- | --- | --- | --- | --- |
| East | 36 | 0.968 (0.027) | 1.003 (0.031) | 1.036 (0.053) | 1.013 (0.033) | 1.034 (0.030) | 1.031 (0.025) |
| East Midlands | 51 | 0.979 (0.030) | 1.006 (0.033) | 1.028 (0.054) | 1.007 (0.028) | 1.015 (0.023) | 1.020 (0.021) |
| North East | 39 | 0.993 (0.032) | 1.006 (0.032) | 1.012 (0.051) | 0.996 (0.034) | 1.004 (0.027) | 1.006 (0.022) |
| North West | 90 | 0.984 (0.029) | 0.998 (0.027) | 1.015 (0.042) | 0.998 (0.028) | 1.010 (0.024) | 1.013 (0.021) |
| Scotland | 68 | 1.024 (0.036) | 0.995 (0.025) | 1.012 (0.063) | 1.006 (0.046) | 0.995 (0.039) | 1.014 (0.041) |
| South East (+London) | 232 | 0.970 (0.023) | 0.998 (0.029) | 1.021 (0.044) | 1.006 (0.029) | 1.027 (0.022) | 1.031 (0.017) |
| South West | 54 | 0.971 (0.028) | 1.003 (0.031) | 1.031 (0.043) | 1.020 (0.028) | 1.038 (0.028) | 1.025 (0.019) |
| Wales | 43 | 0.989 (0.035) | 1.001 (0.031) | 1.018 (0.042) | 0.999 (0.033) | 1.020 (0.030) | 1.015 (0.021) |
| West Midlands | 72 | 0.986 (0.024) | 1.003 (0.028) | 1.019 (0.045) | 1.005 (0.029) | 1.015 (0.026) | 1.023 (0.021) |
| Yorkshire and the Humber | 66 | 0.983 (0.032) | 1.004 (0.033) | 1.021 (0.046) | 1.003 (0.026) | 1.013 (0.021) | 1.016 (0.018) |
|  |  |  |  |  |  |  |  |
| **Region** | **N** | **July** | **August** | **September** | **October** | **November** | **December** |
| East | 36 | 1.031 (0.022) | 1.002 (0.028) | 1.026 (0.040) | 0.975 (0.036) | 0.947 (0.037) | 0.933 (0.030) |
| East Midlands | 51 | 1.016 (0.025) | 1.001 (0.028) | 1.036 (0.032) | 0.982 (0.030) | 0.960 (0.038) | 0.950 (0.031) |
| North East | 39 | 1.018 (0.026) | 1.003 (0.030) | 1.037 (0.032) | 0.993 (0.031) | 0.971 (0.030) | 0.961 (0.028) |
| North West | 90 | 1.017 (0.021) | 1.006 (0.026) | 1.037 (0.028) | 0.994 (0.031) | 0.970 (0.029) | 0.958 (0.029) |
| Scotland | 68 | 1.018 (0.036) | 1.010 (0.036) | 0.992 (0.036) | 1.044 (0.047) | 0.962 (0.041) | 0.928 (0.042) |
| South East (+London) | 232 | 1.030 (0.017) | 1.003 (0.020) | 1.029 (0.030) | 0.985 (0.030) | 0.958 (0.034) | 0.943 (0.031) |
| South West | 54 | 1.026 (0.019) | 0.999 (0.026) | 1.029 (0.031) | 0.977 (0.030) | 0.949 (0.033) | 0.932 (0.031) |
| Wales | 43 | 1.017 (0.025) | 1.000 (0.025) | 1.036 (0.036) | 0.986 (0.031) | 0.963 (0.037) | 0.957 (0.036) |
| West Midlands | 72 | 1.025 (0.018) | 1.000 (0.025) | 1.025 (0.034) | 0.985 (0.032) | 0.961 (0.032) | 0.954 (0.030) |
| Yorkshire and the Humber | 66 | 1.020 (0.025) | 0.999 (0.025) | 1.033 (0.028) | 0.984 (0.027) | 0.966 (0.031) | 0.959 (0.032) |

N indicates the mean number of births per year in thousands

For each month the mean and (standard deviation) are shown

# MOB data from our own multiple sclerosis database

We have been recruiting UK patients for genetic studies for more than 20 years, initially these efforts were primarily local (in the East of England) but now involve a network of recruiting centres spread out across the UK. On the 4^th^ of September 2012 there were 15765 index affected cases listed on our data base. YOB and MOB data were available from 12198 of these individuals who were therefore considered as a case MOB data set. Figure S11 shows that, as expected, the majority of these individuals were born between 1940 and 1979, while figure S12 shows the anticipated non-uniform distribution of recruitment between Government Office Regions (GOR).


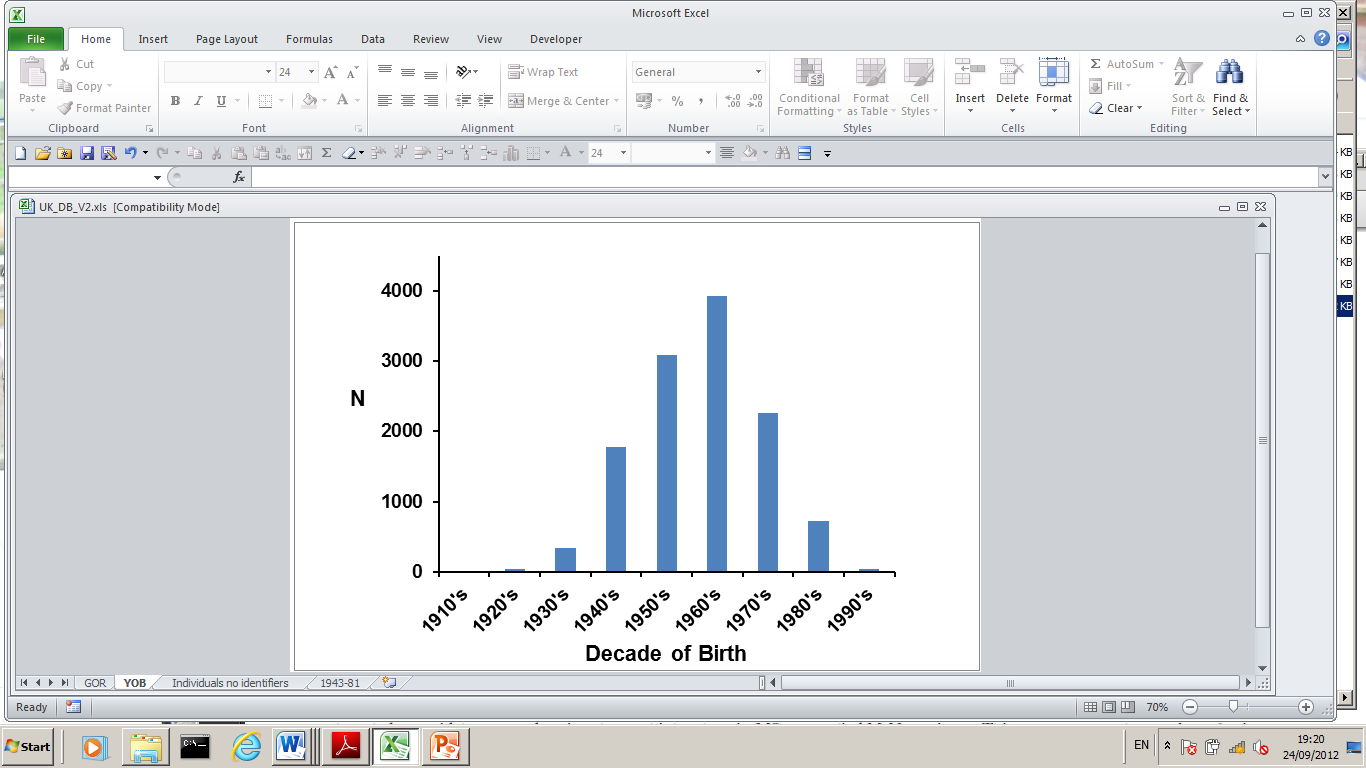


**Figure S11.** The decade of birth in the case MOB data set.


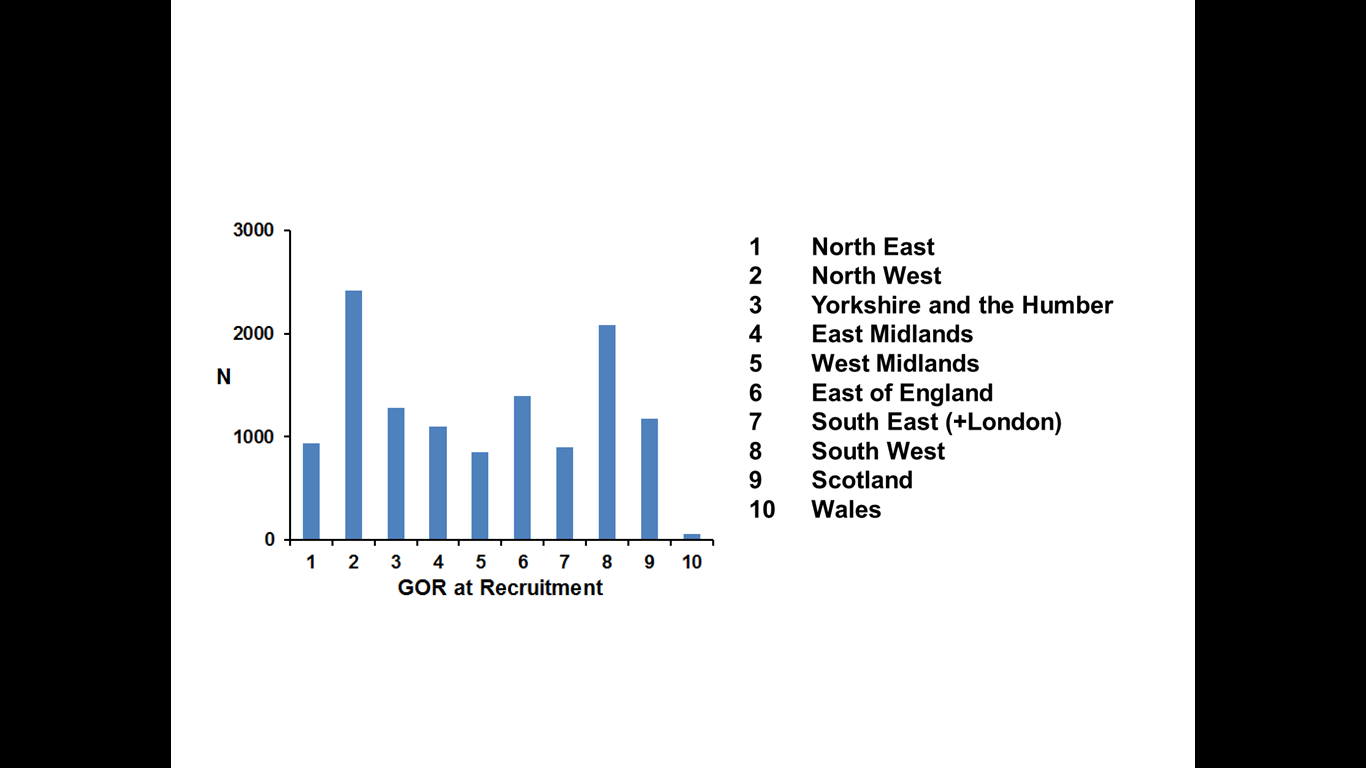


**Figure S12.** Number of cases recruited from each GOR (we don’t have records regarding the place of birth, but can expect a high degree of correlation with the place of recruitment for most).

The number of individuals born in each month in our 12198 cases is shown in table S6 together with the corresponding number of births that would be predicted based on a simple un-weighted average of the data available from the 67 UK MOB data sets; i.e. without any attempt to weight the records or match the distribution of years of birth or regional origin seen in the cases. Analysis of these data gives a nominally significant deficit in December (p=0.03) and a trend towards excess in March with borderline significance (p=0.06). This analysis thus gives the appearance of being consistent with the previously reported MOB effect in multiple sclerosis. However, there are considerable mismatches in both YOB and regional origin between these data sets; some cases (195) have no data for their YOB and some of the included MOB data sets relate to years for which there are no cases (1997-2004). Similarly there is no allowance for the unequal matching of regional origin between the cases and these crude controls.

**Table S6** Number of births in each month observed in our case data set (12198 individuals) with corresponding expected counts calculated by a simple un-weighted average across all 67 UK MOB data sets.

| Month | Cases | Crude Controls |
| --- | --- | --- |
| Jan | 1007 | 1019 |
| Feb | 956 | 955 |
| Mar | 1161 | 1076 |
| Apr | 1071 | 1027 |
| May | 1062 | 1072 |
| Jun | 1037 | 1026 |
| Jul | 1022 | 1050 |
| Aug | 1027 | 1021 |
| Sep | 1042 | 1019 |
| Oct | 1011 | 1004 |
| Nov | 912 | 947 |
| Dec | 890 | 982 |

Total number of cases 12198

Using the GOR specific MOB data sets and restricting the analysis to just those cases for which we have data from the corresponding year reduces the sample size to 5353 but allows a more complete matching. Table S7 shows the number of cases born in each month together with the expected number calculated on the basis of a weighted average across the GOR MOB data sets, weighted according to the number of cases born in each region in each year. These data show no evidence of any significant difference in any month, even after inflating the sample size to 12198 (see fig S13).

**Table S7** Month specific births observed in that sub-group of patients born in or later than 1965 (5353 individuals) with corresponding expected counts calculated by a weighted average across the 440 UK GOR and year specific MOB data sets (weighted according to the number of cases born in each region in each year).

| Month | Cases | Matched Controls |
| --- | --- | --- |
| Jan | 470 | 452 |
| Feb | 422 | 423 |
| Mar | 507 | 484 |
| Apr | 476 | 451 |
| May | 453 | 468 |
| Jun | 474 | 448 |
| Jul | 451 | 459 |
| Aug | 426 | 448 |
| Sep | 451 | 442 |
| Oct | 430 | 441 |
| Nov | 392 | 412 |
| Dec | 401 | 426 |

Total number of cases 5353


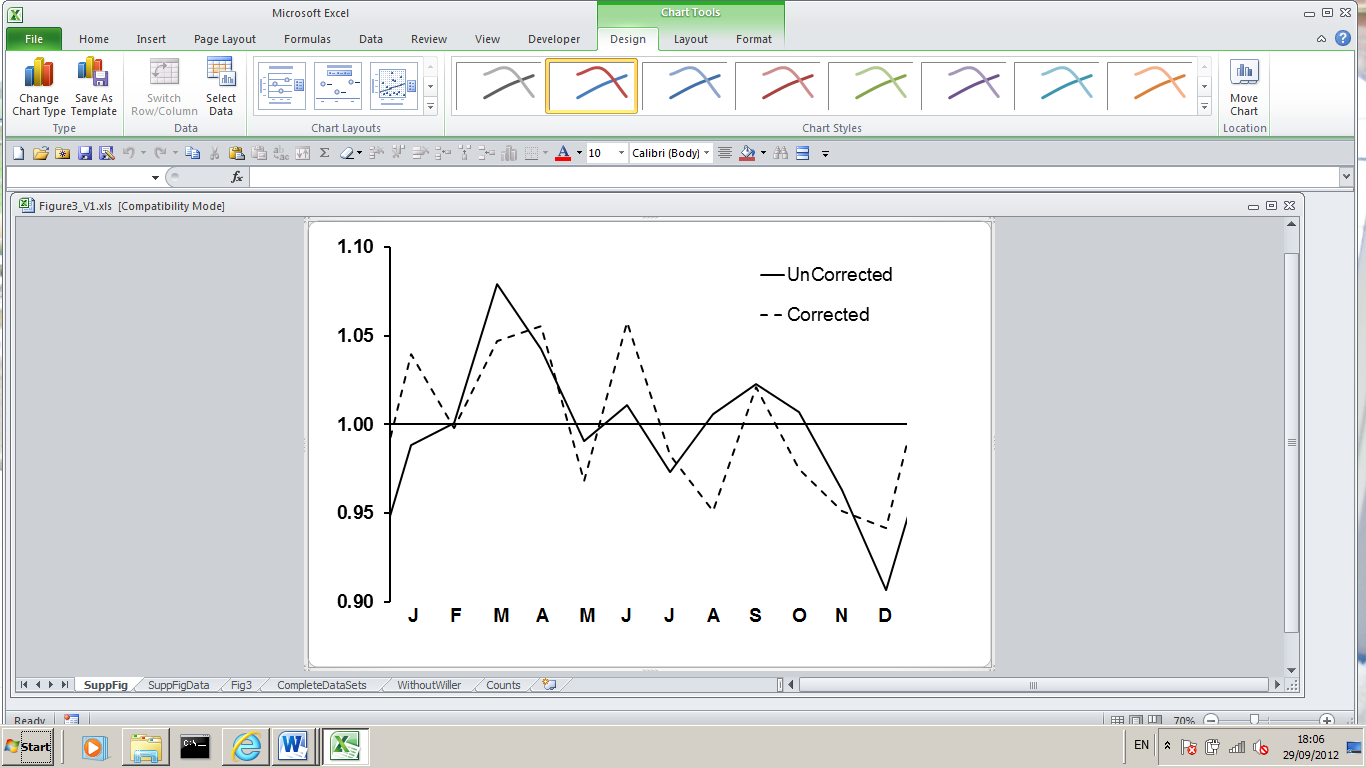


**Figure S13.** The odds ratio for each month based on the full data set of 12198 cases compared to uncorrected controls (solid line) and that sub-group of the cases for which we have region specific data (5353 individuals) as compared with corrected controls (dashed line).

# Testing for the effect of MOB on Mortality

One of the major challenges facing researchers looking for association with environmental risk factors is the difficulty of reliably measuring exposure to the factor of interest. In this regard MOB is a notable exception. Most people know their MOB and since detailed birth records are freely available for most developed countries this variable can be measured with a very high degree of accuracy However, we might question the validity of comparing MOB in prevalent cases with records for the corresponding YOB, where the correct denominator should be the distribution of MOB in those still alive and of the same age as the recruited cases. If MOB influenced mortality then the distribution of MOB present in the population of individuals of any particular age might be distorted with respect to that reflected in records for the corresponding YOB. To explore this possibility we compared the MOB distribution for each YOB between 1938 and 1970 as seen in the UK census for 2001 with the corresponding distribution in the birth records for those years (ignoring the confounding influences of migration). Unsurprisingly we found no evidence for any difference in mortality related to MOB.

# Using unaffected siblings as controls

The use of unaffected siblings (or other unaffected relatives) as controls is expected to reduce confounding related to regional influences on MOB and would therefore seem to be a logical way to attempt to identify MOB effects. However, since unaffected siblings are necessarily unmatched for YOB (in the vast majority of families) and national statistics shows a highly significant variation in MOB patterns between years within any given region the potential for confounding remains. Furthermore since a woman cannot fall pregnant with a second child while she is still pregnant with a first we might expect a degree of negative correlation in MOB between siblings especially amongst those of a similar age; an effect which could inflate any apparent MOB effect found using such controls.^7^

To date three groups have considered unaffected siblings in their analysis of MOB in multiple sclerosis.^7, 8, 18^ The first considered MOB in 4232 unaffected siblings and reported a deficit of November births amongst their matched cases (273/4232 in the cases and 343/4232 in the unaffected siblings).^7^ However, based on the proportion of births occurring in November that these authors reported for the general Canadian population (1373/17874)^7^ the expected number of November births in each group would have been approximately 325/4232; which is only marginally different from that seen in the cases (p=0.03) and not significantly different from that seen in the unaffected siblings (p=0.47). No data on other months was provided in the paper so presumably this was the only month showing an apparent difference between cases and their unaffected siblings.^7^ The second group identified 1069 unaffected siblings for 381 cases and reported a marginally significant excess of case births in spring, but found no significant evidence for a deficit in November.^8^ The third group considered 8956 unaffected siblings and 5711 unaffected mothers for their 6649 cases.^18^ This group did not compare their cases with these unaffected cohorts but instead confirmed that neither of these cohorts showed any statistically significant evidence for deviation from the MOB seen in the uncorrected Norwegian population control data. Based on the data these authors provided in their supplementary file^18^ we found no evidence for any statistically significant deficit in November births amongst their cases when compared with either unaffected siblings or unaffected mothers (marginally significant evidence for excess in April is seen, especially when compared with mothers).^18^ Given the modest numbers of unaffected relatives available it is unsurprising that none of the three groups found any evidence for a MOB effect amongst their unaffected relative cohorts (see figure 4 in the main paper). Furthermore the lack of any substantial consistency between these studies is in keeping with the notion that any apparent differences between cases and their unaffected relatives are more likely to have arisen from uncorrected structure than any genuine MOB effect.

# Heat map (choropleth) representations of seasonal excess birth rate data

Figures S14 and S15, reproduce the data presented in Figure S5 in the form of heat maps, S14 for North America and S15 for Europe.

| **January** | **February** | **March** |
| --- | --- | --- |
| **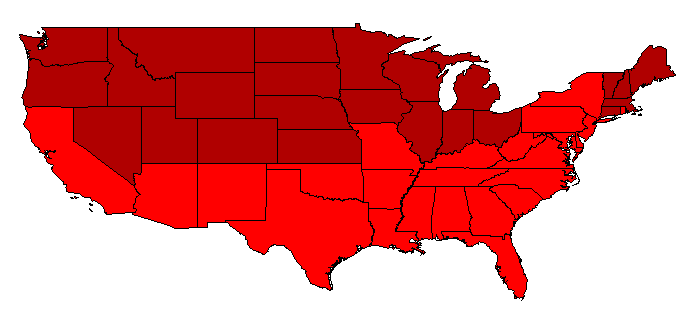** | **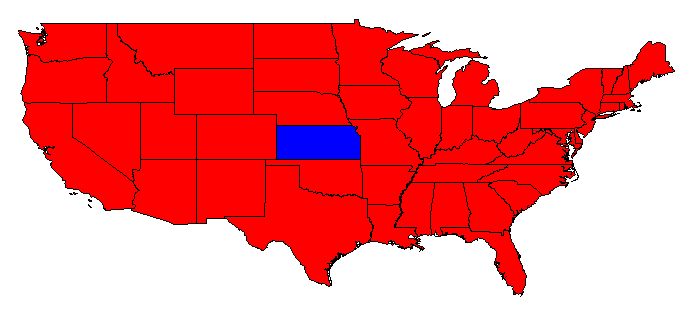** | **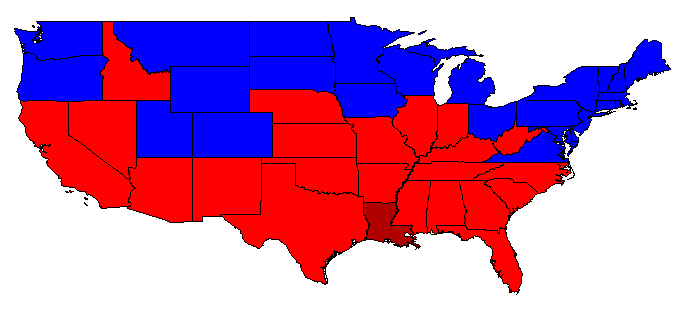** |
| **April** | **May** | **June** |
| **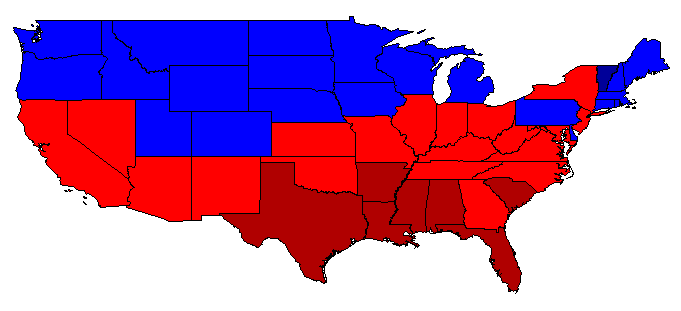** | **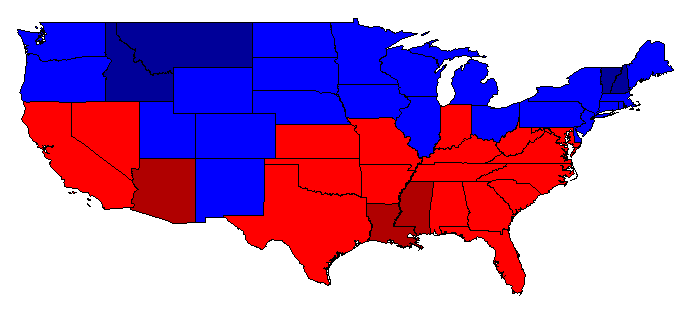** | **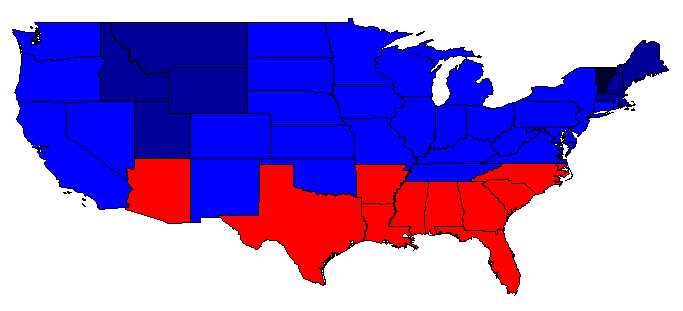** |
| **July** | **August** | **September** |
| **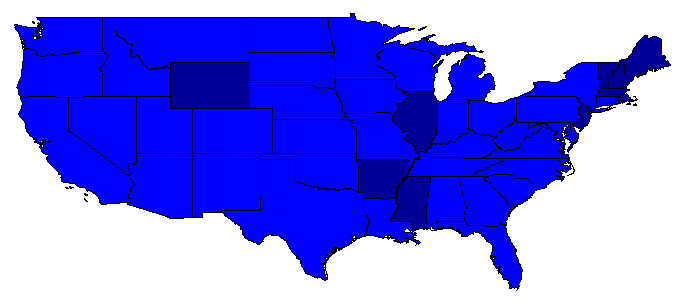** | **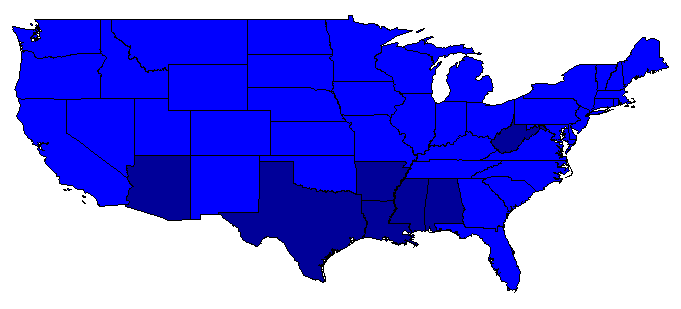** | **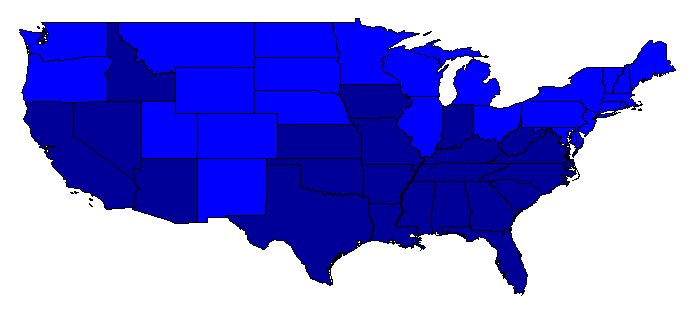** |
| **October** | **November** | **December** |
| **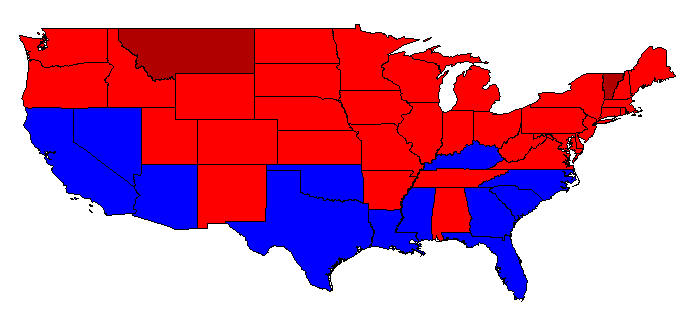** | **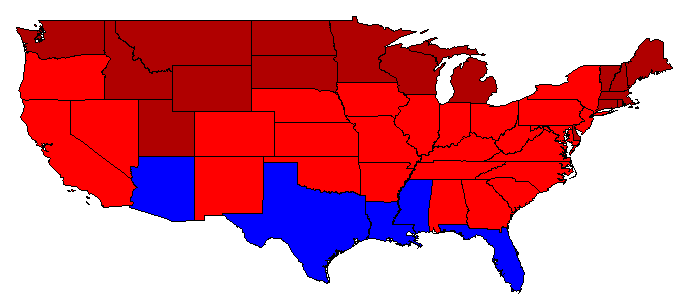** | **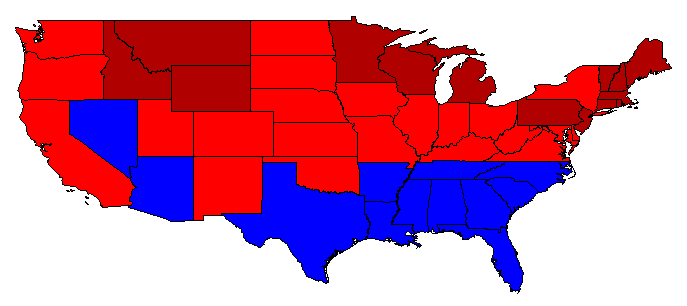** |

|  | -10% to -5% |
| --- | --- |
|  | -5% to 0% |
|  | 0% to 5% |
|  | 5% to 10% |

**Figure S14**. Excess average normalised daily birth rate in USA states plotted as a heat map as per the legend opposite. Values are based on data from the 10 year period 1991-2000. Maps created using the public domain software Epi Info™ version 3.5.4.

| **January** | **February** | **March** |
| --- | --- | --- |
| **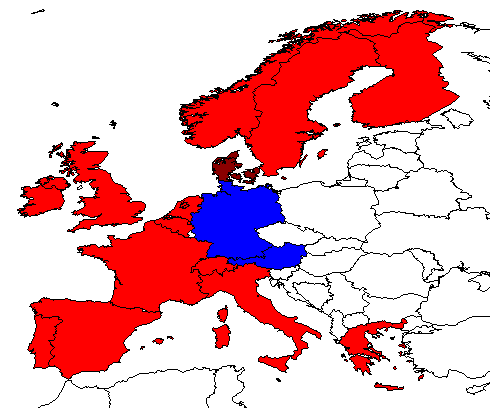** | **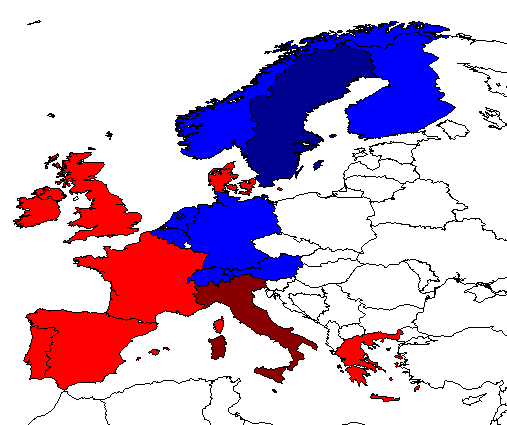** | **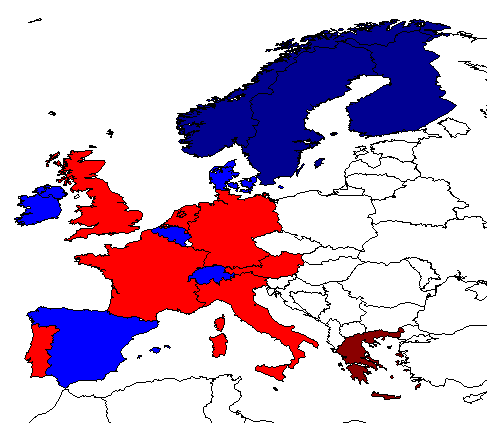** |
| **April** | **May** | **June** |
| **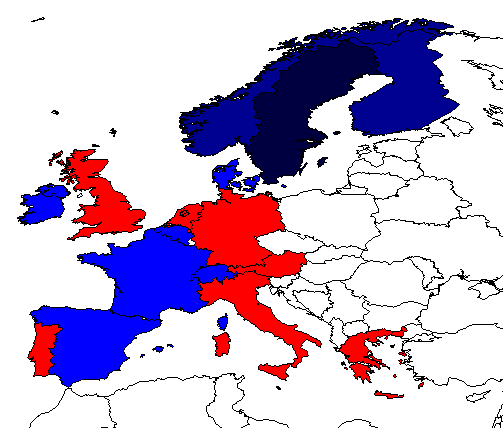** | **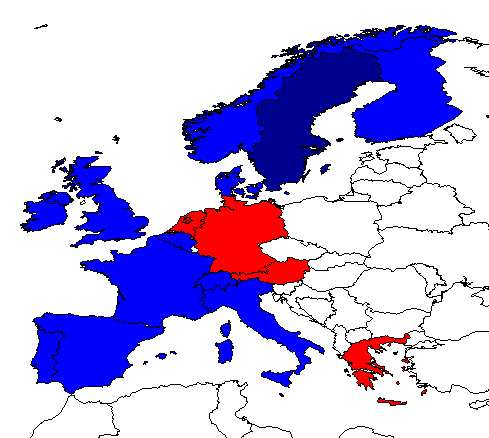** | **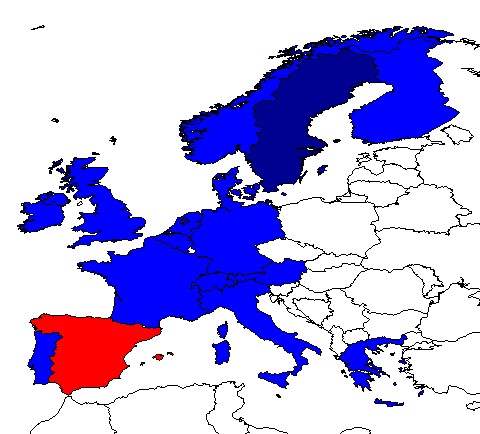** |
| **July** | **August** | **September** |
| **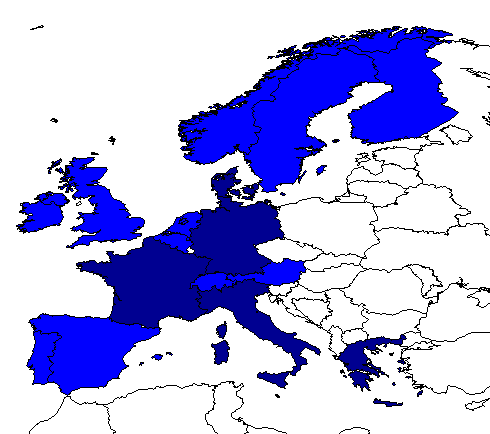** | **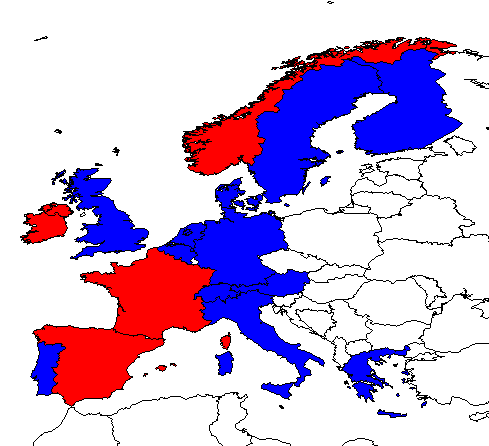** | **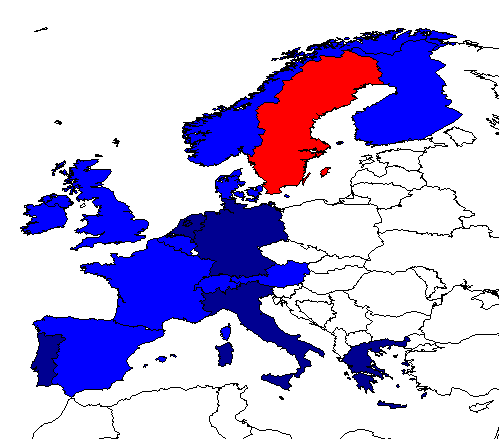** |
| **October** | **November** | **December** |
| **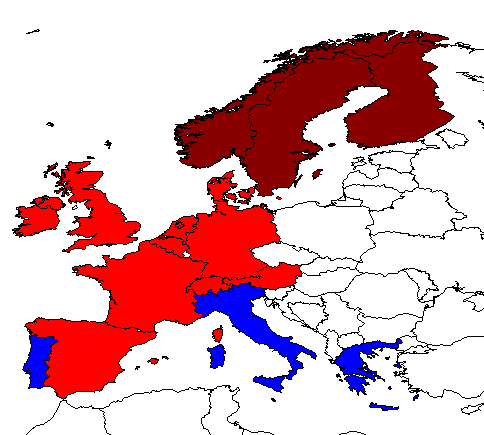** | **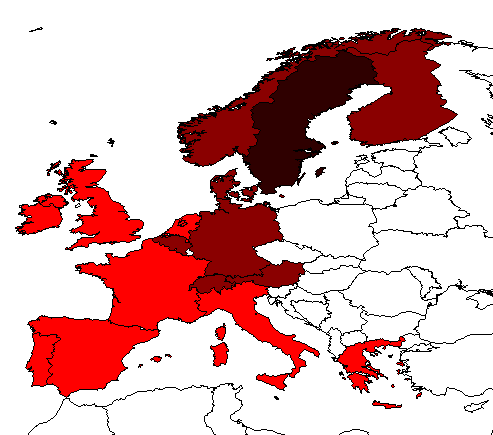** | **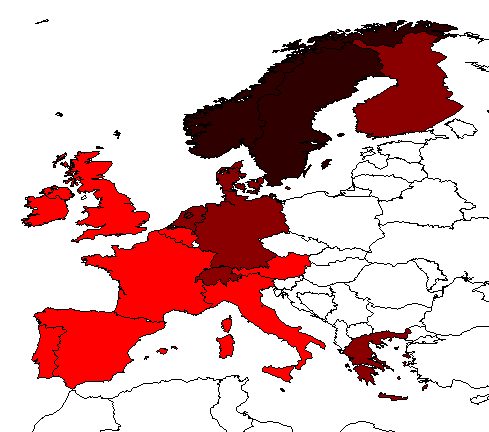** |

|  | -20% to -10% |
| --- | --- |
|  | -10% to -5% |
|  | -5% to 0% |
|  | 0% to 5% |
|  | 5% to 10% |
|  | 10% to 20% |

**Figure S15**. Excess average normalised daily birth rate in 16 European countries plotted as a heat map as per the legend opposite. Values are based on data from the 10 year period 1991-2000. Map created using the public domain software Epi Info™ version 3.5.4 (note Northern Ireland and Eire incorrectly shown as one country).

# References

1. Compston A, Confavreux C, Lassmann H, et al. McAlpine's Multiple Sclerosis. Fourth ed. London: Churchill Livingstone; 2006.

2. Ahlgren C, Oden A, Lycke J. High nationwide prevalence of multiple sclerosis in Sweden. Mult Scler. 2011;17:901-8.

3. Templer DI, Trent NH, Spencer DA, et al. Season of birth in multiple sclerosis. Acta Neurol Scand. 1992;85:107-9.

4. Wiberg MA, Templer DI. Season of birth in multiple sclerosis in Sweden: Replication of Denmark Findings. J Orthomol Med. 1994;9:71-4.

5. Bharanidharan P. Monthly distribution of multiple sclerosis patients' births. Int J Biometeorol. 1997;40:117-8.

6. Salemi G, Ragonese P, Aridon P, et al. Is season of birth associated with multiple sclerosis? Acta Neurol Scand. 2000;101:381-3.

7. Willer CJ, Dyment DA, Sadovnick AD, Rothwell PM, Murray TJ, Ebers GC. Timing of birth and risk of multiple sclerosis: population based study. BMJ. 2005;330:120.

8. Sotgiu S, Pugliatti M, Sotgiu MA, et al. Seasonal fluctuation of multiple sclerosis births in Sardinia. J Neurol. 2006;253:38-44.

9. Fernandes de Abreu DA, Babron MC, Rebeix I, et al. Season of birth and not vitamin D receptor promoter polymorphisms is a risk factor for multiple sclerosis. Mult Scler. 2009;15:1146-52.

10. Salzer J, Svenningsson A, Sundstrom P. Season of birth and multiple sclerosis in Sweden. Acta Neurol Scand. 2010;121:20-3.

11. Bayes HK, Weir CJ, O'Leary C. Timing of birth and risk of multiple sclerosis in the Scottish population. Eur Neurol. 2010;63:36-40.

12. Staples J, Ponsonby AL, Lim L. Low maternal exposure to ultraviolet radiation in pregnancy, month of birth, and risk of multiple sclerosis in offspring: longitudinal analysis. Bmj. 2010;340:c1640.

13. Fragoso YD, Shearer KD, Adoni T, et al. Month of Birth Does Not Seem to Interfere with the Development of Multiple Sclerosis Later in Life in Brazilian Patients. Neuroepidemiology. 2012;39:70-1.

14. Givon U, Zeilig G, Dolev M, Achiron A. The month of birth and the incidence of multiple sclerosis in the Israeli population. Neuroepidemiology. 2012;38:64-8.

15. Menni C, Lowell WE, Bentzen J, et al. Short and long term variation in ultraviolet radiation and multiple sclerosis. Int J Environ Res Public Health. 2012;9:685-97.

16. Saastamoinen KP, Auvinen MK, Tienari PJ. Month of birth is associated with multiple sclerosis but not with HLA-DR15 in Finland. Mult Scler. 2012;18:563-8.

17. Verheul F, Smolders J, Trojano M, et al. Fluctuations of MS births and UV-light exposure. Acta Neurol Scand. 2012.

18. Grytten N, Torkildsen O, Aarseth JH, et al. Month of birth as a latitude-dependent risk factor for multiple sclerosis in Norway. Mult Scler. 2012.

19. Koch-Henriksen N, Sorensen PS. The changing demographic pattern of multiple sclerosis epidemiology. Lancet Neurol. 2010;9:520-32.

20. Kurtzke JF, Beebe GW, Norman JE, Jr. Epidemiology of multiple sclerosis in U.S. veterans: 1. Race, sex, and geographic distribution. Neurology. 1979;29:1228-35.

21. Ford HL, Gerry E, Johnson M, Williams R. A prospective study of the incidence, prevalence and mortality of multiple sclerosis in Leeds. J Neurol. 2002;249:260-5.

22. Hirst C, Ingram G, Pickersgill T, Swingler R, Compston DA, Robertson NP. Increasing prevalence and incidence of multiple sclerosis in South East Wales. J Neurol Neurosurg Psychiatry. 2009;80:386-91.

23. Robertson N, Deans J, Fraser M, Compston DA. Multiple sclerosis in south Cambridgeshire: incidence and prevalence based on a district register. J Epidemiol Community Health. 1996;50:274-9.

24. Williams ES, McKeran RO. Prevalence of multiple sclerosis in a south London borough. Br Med J (Clin Res Ed). 1986;293:237-9.

25. Rice-Oxley M, Williams ES, Rees JE. A prevalence survey of multiple sclerosis in Sussex. J Neurol Neurosurg Psychiatry. 1995;58:27-30.

26. Zajicek JP, Ingram WM, Vickery J, Creanor S, Wright DE, Hobart JC. Patient-orientated longitudinal study of multiple sclerosis in south west England (The South West Impact of Multiple Sclerosis Project, SWIMS) 1: protocol and baseline characteristics of cohort. BMC Neurol. 2010;10:88.

27. Visser EM, Wilde K, Wilson JF, Yong KK, Counsell CE. A new prevalence study of multiple sclerosis in Orkney, Shetland and Aberdeen city. J Neurol Neurosurg Psychiatry. 2012;83:719-24.

28. Agresti A. An introduction to Categorical Data Analysis: John Wiley and Sons; 2007.

29. Venables WN, Ripley BD. Modern Applied Statistics. Fourth ed. New York: Springer; 2002.
